# On‐DNA Transfer Hydrogenolysis and Hydrogenation for the Synthesis of DNA‐Encoded Chemical Libraries

**DOI:** 10.1002/anie.202111927

**Published:** 2021-11-27

**Authors:** Harriet A. Stanway‐Gordon, Jessica S. Graham, Michael J. Waring

**Affiliations:** ^1^ Cancer Research UK Newcastle Drug Discovery Unit Chemistry School of Natural and Environmental Sciences Newcastle University Bedson Building Newcastle upon Tyne NE1 7RU UK

**Keywords:** DNA encoded libraries, Hydrogenation, Hydrogenolysis, Micellar catalysis

## Abstract

DNA‐encoded libraries (DELs) are an increasingly popular approach to finding small molecule ligands for proteins. Many DEL synthesis protocols hinge on sequential additions of monomers using split‐pool combinatorial methods. Therefore, compatible protecting group strategies that allow the unmasking of reactive functionality (e. g. amines and alcohols) prior to monomer coupling, or the removal of less desirable functionality (e. g., alkenes and alkynes) are highly desirable. Hydrogenation/hydrogenolysis procedures would achieve these ends but have not been amenable to DEL chemistry. We report a catalytic hydrogen transfer reaction using Pd/C, HCONH_4_ and the micelle‐forming surfactant, TPGS‐750‐M, which gives highly efficient conversions for hydrogenolysis of Cbz‐protected amines and benzyl protected alcohols and hydrogenation of nitros, halides, nitriles, aldehydes, alkenes and alkynes. Application to multicycle synthesis of an encoded compound was fully compatible with DNA‐amplification and sequencing, demonstrating its applicability to DEL synthesis. This method will enable synthetic DEL sequences using orthogonal protecting groups.

## Graphical abstract

A catalytic hydrogen transfer reaction using Pd/C, HCONH_4_ and the micelle‐forming surfactant, TPGS‐750‐M for hydrogenolysis of Cbz‐protected amines and benzyl protected alcohols and hydrogenation of alkenes, alkynes, nitros, nitriles, halides and aldehydes of DNA‐conjugated substrates is described. The methodology is fully compatible with DNA‐amplification and sequencing, demonstrating its applicability to DEL synthesis. This method will enable synthetic DEL sequences using orthogonal protecting groups.

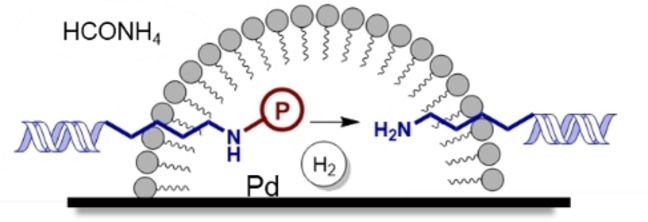

DNA encoded libraries (DELs) are a highly efficient approach to hit finding in medicinal chemistry and chemical biology.[^1^, ^2^, ^3^, ^4^] In perhaps their most widely used application, libraries of compounds are synthesised by split and pool combinatorial chemistry, starting with a DNA conjugated organic substrate, commonly referred to as a *headpiece*. Sequential monomers are added to the headpiece, with each synthetic step accompanied by a ligation of a codon to the DNA tag uniquely corresponding to each monomer. By repeating this operation over multiple cycles, libraries containing huge (potentially billions) of compounds can be prepared. The resulting libraries can be screened for binding to protein targets by affinity selection and active compounds identified by PCR amplification and DNA sequencing. The use of DNA tags removes the need for complex sample storage and processing facilities associated with traditional compound libraries and provides one of the most efficient means of screening synthetic compounds for biological activity.[^5^, ^6^, ^7^, ^8^, ^9^, ^10^]

The success of the approach depends critically on the efficiency of the chemistry that is used to construct the DEL. “On‐DNA” chemistry is usually carried out in water, due to the insolubility of DNA‐conjugates in organic solvents, and the presence of DNA obviates the use of many commonly employed reagents, including acids, oxidising agents and strong bases.[^11^, ^12^] Because limited purification is carried out at each synthesis step, typically a solvent wash followed by ethanol precipitation of the DNA conjugates from the aqueous reaction mixture, it is essential for the fidelity of the library that the reactions used in each stage proceed cleanly with high conversions across a range of substrates. Examples of methods that fulfill these criteria are highly limited. We,[^13^, ^14^] and others,^[15]^ have recently devepoled micellar catalysis to DEL synthesis as a means of increasing the scope and efficiency of on‐DNA chemistry and applied it to the multicycle synthesis of DELs. Our methods thus far have used the commercially available surfactant TPGS‐750‐M,^[16]^ providing operationally simple procedures.

Multicycle libraries, in which a series of monomers are added sequentially to the headpiece (Figure 1a) require orthogonally compatible chemistries for each step to allow reliable production of the intended final products. A highly versatile way to achieve this control is to use orthogonal protecting groups, which allow specific reactive groups to be sequentially unmasked, particularly amines from Cbz‐protected amines or nitro species (Figure 1b). Many protecting groups commonly employed in traditional organic chemistry are not applicable to DELs due to the incompatibility of conditions required for their removal. For example, Boc protection of amines is typically not possible due to the need for acid mediated deprotection.^[11]^ Thus, Fmoc has been most commonly used for amine protection, which has disadvantages due to its lability and the need to synthesise diverse Fmoc protected monomers.

**Figure 1:**
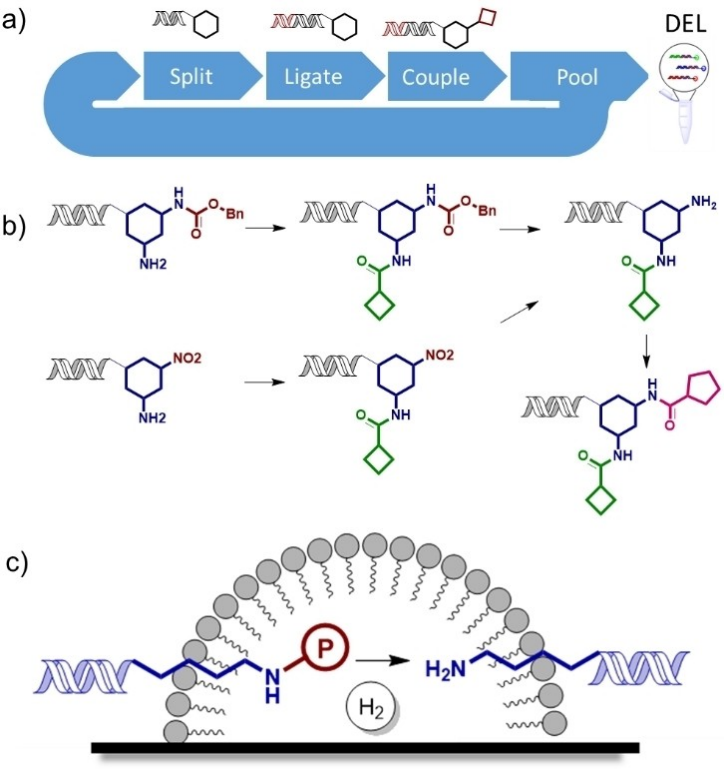
a) Schematic representation of multicycle DEL synthesis; b) Example multicycle DEL involving an amide coupling, Cbz‐protected amine deprotection/nitro reduction, second amide coupling sequence; c) Postulated on‐DNA transfer hydrogenation by association of micelles with DNA‐tagged substrates on the catalyst surface.

The development of conditions for on‐DNA hydrogenation/hydrogenolysis would be of great utility in the synthesis of DELs. It would allow the deprotection of multiple oxygen and nitrogen protecting groups (benzyl ethers and carbamates, for example), unmasking of reactive functionality by reduction (nitro or nitrile groups to amines) as well as reduction of other moieties. Manipulation of hydrogen gas using parallel reaction arrays used in DEL synthesis is not practical.^[11]^ We therefore investigated the application of micellar methodology to on‐DNA hydrogenation using catalytic hydrogen transfer. Encouragingly, catalytic hydrogenation using micelle forming surfactants with hydrogen gas has been recently reported.[^17^, ^18^, ^19^] It has been shown that TPGS‐750‐M derived micelles can associate with metal surfaces in aqueous systems^[20]^ and that the surfactant MC‐1 associates with palladium on carbon.^[21]^ We hypothesised that this system could promote on‐DNA hydrogenation by localistion of the substrate bearing portion of the DNA conjugate to within a catalyst associated micelle structure in a manner analogous to that which is believed to occur in solution phase on‐DNA micellar reactions (Figure 1c). If this could be combined with the palladium on carbon/ammonium formate catalytic hydrogen transfer system, it would provide a practical method for hydrogenation and hydrogenolysis of on‐DNA substrates.

Initially, the hydrogenolysis of Cbz‐protected amines and benzyl‐protected alcohols was investigated. Ten carboxylic acids containing a range of benzyl carbamates (**2**–**8**, Table 1), benzylethers (**9**–**11**, Table 1), aryl nitro‐ and halo‐derivatives (**12**–**20**, Table 2) as well as alkenes, alkynes, nitriles and aldehydes (**21**–**25**, Table 2) were coupled to the headpiece amino‐PEG4‐hexylamido‐DNA **1** (see SI) using established conditions.^[14]^ This afforded a range of substrates for the initial investigation. Treatment of these substrates with palladium on carbon and ammonium formate in the presence of TPGS‐750‐M (either 2 or 3 % in water), proceeded very well without the need for extensive optimisation (Table 1).

**Table 1:** Scope of the on‐DNA Cbz and benzyl ether hydrogenolysis. 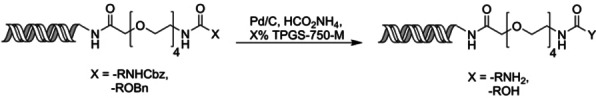

−X	−Y	Conversion [%]^[a]^	Conversion [%]^[b]^
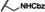	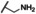	100	100
**2**	**27**		
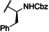	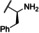	100^[c]^	100
**3**	**28**		
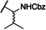	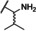	100^[c]^	100
**4**	**29**		
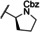	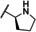	100^[c]^	100
**5**	**30**		
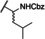	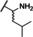	100	100
**6**	**31**		
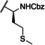	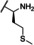	100 (90)	100 (94)
**7**	**32**		
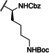	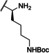	100	100
**8**	**33**		
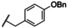	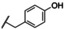	100	100^[c]^
**9**	**34**		
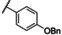	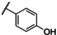	100	100^[c]^
**10**	**35**		
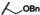	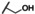	18	100
**11**	**36**		

Conditions: DNA conjugated X (1–5 nmol), 6.25 mM 10 wt % Pd/C, 0.5 M HCO_2_NH_4_, TPGS‐750‐M, rt, 1200 rpm, 1 hr. Figures in parentheses show the total percentage of desired product formed relative to by‐products where this was not 100 %. [a] 2 % TPGS‐750‐M. [b] 3 % TPGS‐750‐M. [c] 2 hours.

**Table 2:** On‐DNA transfer hydrogenation of nitro groups, halides, alkenes, nitriles and aldehydes. 
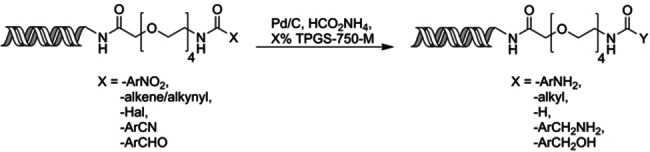

−X	−Y	Conversion [%]^[a]^	Conversion [%]^[b]^
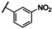	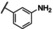	100	100
**12**	**37**		
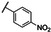	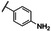	100	100
**13**	**38**		
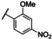	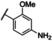	100	100
**14**	**39**		
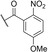	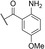	100	100
**15**	**40**		
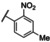	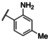	100^[c]^	100
**16**	**41**		
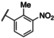	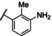	100	100
**17**	**42**		
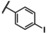	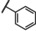	100	100
**18**	**43**		
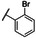	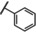	60	100
**19**	**43**		
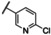	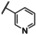	0	100 (95)
**20**	**44**		
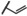	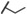	100	100
**21**	**45**		
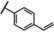	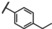	100	100
**22**	**46**		
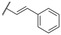	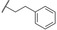	0	100
**23**	**47**		
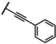	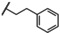	100	100
**24**	**47**		
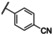	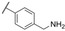	48 (39)	97 (67)
**25**	**48**		
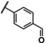	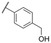	100 (91)	100 (94)
**26**	**49**		

Conditions: DNA conjugated X (1–5 nmol), 6.25 mM 10 wt % Pd/C, 0.5 M HCO_2_NH_4_, TPGS‐750‐M, rt, 1200 rpm, 1 hr. Figures in parentheses show the total percentage of desired product formed relative to by‐products where this was not 100 %. [a] 2 % TPGS‐750‐M. [b] 3 % TPGS‐750‐M. [c] 2 hours.

The hydrogenolysis of Cbz‐protected glycine **2** proceeded smoothly to reveal glycine **27** with 100 % conversion (Table 1). Substituted Cbz‐protected aminoacids phenyl alanine **3**, valine **4**, proline **5** and leucine **6** also gave 100 % conversion to amines **28–31**. In the presence of 2 % surfactant, **4** and **6** required a slightly longer reaction time (2 hours) to achieve full conversion. Methionine **7** gave 100 % conversion, thus showing that the reaction proceeds in the presence of the thioether, a potential catalyst poison. In the presence of 2 % surfactant, a small amount (7 %) of C−S hydrogenolysis (loss of ‐SMe) was observed, which was not seen with 3 % surfactant. In both cases there were also traces of uncharacterised byproducts formed. ϵ‐Boc,α‐Cbz‐lysine **8** underwent hydrogenolysis of the Cbz group with 100 % conversion to monoamine **33** with the Boc group fully intact, showing that orthogonal amine deprotections can be readily achieved.

4‐Benzyloxy‐ phenylacetamide **9** and benzamide **10** underwent benzyl ether cleavage cleanly with full conversion to phenols **34** and **35**. Interestingly, the aliphatic ether benzyloxyacetamide **11** required 3 % TPGS‐705‐M to achieve full conversion. These results show that *N*‐Cbz or *O*‐benzyl protecting groups can be quantitatively removed by hydrogenolysis under these conditions on a wide variety of relevant substrates.

Encouraged by the results of the hydrogenolysis, hydrogenation reactions of a range of functional groups were investigated under the same conditions (Table 2). Aromatic nitro groups (compounds **12**–**17**) reduced quantitatively to the corresponding anilines **37**–**42**, showing that a range of *o*‐, *m*‐, *p*‐ substituents including methyl‐ and methoxy‐ are tolerated. The 2‐nitro‐4‐methyl substrate **16** required longer reaction time with 2 % surfactant.

Aryl halides **18**–**20** also underwent reduction under the same conditions, although *o*‐bromophenyl **19** required 3 % TPGS to achieve full conversion. The 2‐chloropyridine **20** gave no reaction with 2 % but proceeded to 100 % conversion with 3 % surfactant. In this case 5 % hydrolysis to the pyridone was also observed.

Alkene (**22** and **23**) and alkyne (**24**) containing substrates underwent quantitative hydrogenation to the corresponding alkanes **46** and **47**, with the *trans*‐disubstituted alkene **23** requiring 3 % surfactant for the reaction to proceed. The benzonitrile **25** reduced less well but still gave appreciable amounts of the desired benzylamine **48**, especially with 3 % surfactant (97 % conversion, 67 % product). The *p*‐benzaldehyde **26** reduced to benzyl alcohol **49** efficiently, with slightly cleaner reaction at the higher surfactant concentration (94 % vs. 91 %). The byproduct of the nitrile and aldehyde reductions corresponded to the methyl derivative in both cases (HRMS), presumably arising from hydrogenolysis of **48** and **49**.

Reduction of alkenes and alkynes is potentially useful in DEL synthesis since these functionalities are sometimes undesirable in screening compounds—being metabolically vulnerable, for example. They could, however, be introduced in library synthesis protocols using, for example, palladium‐mediated couplings. The reduction would be useful in removing this functionality and introducing greater sp^3^ character into the library products.

Comparative experiments established the necessity of agitation and the addition of the surfactant. Carrying out the hydrogenolysis of the *N*‐Cbz‐protected phenylalanine conjugate **3** without shaking resulted in only 67 % conversion to the amine product after 1 hour (see ESI). Carrying out the reactions of the Cbz‐phenylalanine **3**, benzyloxyacetamide **9**, 2‐nitro‐4‐methoxyphenyl **15** and 4‐vinylphenyl **22** in the absence of TPGS‐750‐M resulted in almost total loss of DNA‐conjugated material (<1 % recovery in all cases, Table S3). This, coupled with the increased reactivity with higher surfactant concentrations observed for the less reactive substates, demonstrates the beneficial role that the TPGS‐750‐M plays in both the reaction efficiency and the protection of the DNA.

It is notable that increasing surfactant concentration from 2 to 3 % apparently leads to more forcing reaction conditions. The increased concentration of surfactant may lead to greater solubility of more hydrophobic substrates in the reaction medium. Additionally higher surfactant concentrations may lead to more of the active catalyst surface being associated with the surfactant and therefore a greater effective concentration of palladium hydride species in proximity with the DNA‐conjugated substrates. It has been shown that the rate and extent of substrate adsorption onto the catalyst is an important determinant of the rate of traditional heterogeneous palladium catalysed hydrogenations, especially at high hydrogen coverage.^[22]^ Related to this latter point, it is interesting that *trans*‐alkene **23** was only reduced in the presence of 3 % TPGS‐750‐M whereas the analogous alkyne **24** reduced completely with 2 % surfactant. It has been shown that the adsorption of alkynes to palladium surfaces is energetically more favourable than for the corresponding *trans*‐alkenes.^[22]^ Hence the increased surfactant concentration may be required to promote sufficient adsorption of **23**. This implies that **23** is not an intermediate in the reduction of **24**, which may proceed without dissociation of the intermediates from the catalyst surface (or proceed via the *cis* isomer).

During the course of this work, an alternative method for hydrogenation using Pd(OAc)_2_ and NaBH_4_ as the hydrogen source was published.^[23]^ To compare the performance of our method Cbz‐protected phenyl alanine **3**, benzyloxyacetamide **9**, benzonitrile **25** and benzaldehyde **26** were subjected to the Pd(OAc)_2_/NaBH_4_ conditions (Table S4). They performed similarly for **3** (100 % conversion) but failed to give any conversion with **9** or **25**. Benzaldehyde **26** reduced cleanly to the alcohol.^1^ These results suggest that the micellar hydrogen transfer reaction is comparable with existing methods for Cbz deprotection but is advantageous in that it is capable of hydrogenolysing benzyl ethers.

To demonstrate the applicability of the methodology to DEL synthesis, a representative encoded compound was synthesised using a 3‐cycle sequence of amide coupling, Cbz‐deprotection and second amide coupling (Scheme 1a). Cbz‐protected valine was coupled to headpiece amino‐PEG4‐hexylamido‐DNA **1** to give amide **4**. Cbz‐hydrogenolysis proceeded cleanly to reveal the amine **29**, which underwent a second amide coupling with benzoic acid to give bisamide **50** in 12 % overall yield. The reactions proceeded with quantitative conversions, the reduction in overall yield is likely associated with losses during handling on small scale during this procedure and is not representative of what could be expected in a DEL synthesis protocol (see below).

**Scheme 1:**
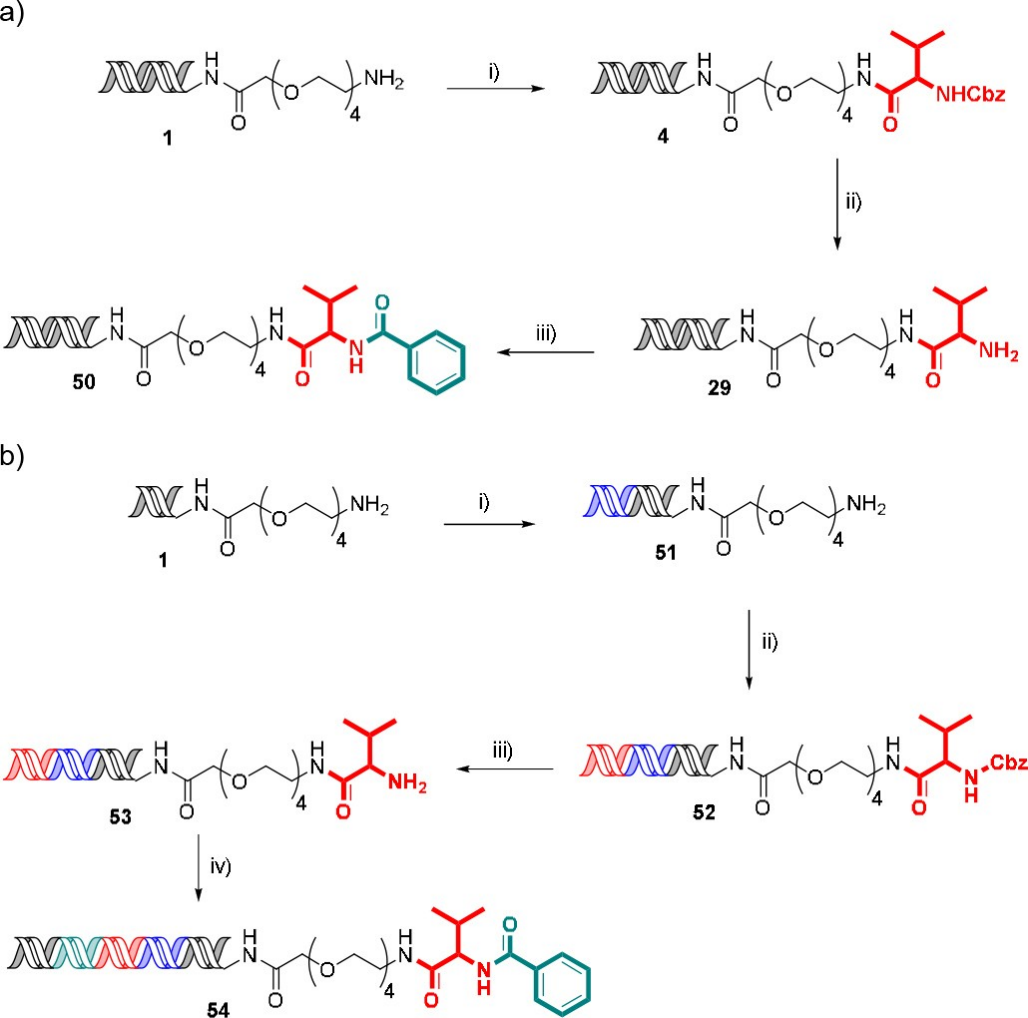
a) Synthesis of 3‐cycle encoded compound. Conditions: i). Cbz‐Val‐OH (0.5 M), HATU (0.5 M), lutidine (2 M), 3.5 % TPGS‐750‐M, 45 °C, 16 h, 46 % yield; ii) 10 wt % Pd/C (6.25 mM), HCO_2_NH_4_ (0.5 M), 2 % TPGS‐750‐M, rt, 1200 rpm, 2 h, 41 % yield; iii) Benzoic acid (0.5 M), HATU (0.5 M), lutidine (2 M), 3.5 % TPGS‐750‐M, 45 °C, 16 h 61 % yield; b) Synthesis of representative encoded compound. Conditions: i) Ligation (primer and library codon), 100 % yield; ii) a. Ligation (first monomer codon), b. Cbz‐Val‐OH (0.5 M), HATU (0.5 M), lutidine (2 M), 3.5 % TPGS‐750‐M, 45 °C, 16 h, iii) 10 wt % Pd/C (6.25 mM), HCO_2_NH_4_ (0.5 M), 2 % TPGS‐750‐M, rt, 1200 rpm, 2 h, 59 % yield over 3 steps; iv) a) Ligation (closing primer and second monomer codon), b) benzoic acid ((0.5 M), HATU (0.5 M), lutidine (2 M), 3.5 % TPGS‐750‐M, 45 °C, 16 h, 58 % yield over 2 steps. Yields determined by Nanodrop™ spectrophotometry.

This synthetic sequence was repeated coupled with DNA codon ligation (Scheme 1b). In this case, the synthesis started with ligation of a primer sequence and unique identifier to give headpiece **51**, followed by ligation of a cycle 1 monomer codon prior to the first amide coupling to give Cbz protected **52**. Cbz hydrogenolysis to amine **53** was followed by ligation of the second codon and closing primer sequence, then acylation with benzoic acid to give fully encoded bisamide **54** in 34 % overall yield. PCR amplification of **54**, followed by next‐generation DNA sequencing proceeded well with 72 % of the reads corresponding to the expected sequence for both the substrate and complementary strands (338 596 total reads).

To quantify the amount of amplifiable DNA recovered after the hydrogenation reaction, a sample of the coded substrate was subjected to the reaction conditions. Equal quantities of the starting material and recovered DNA from the reaction were subjected to qPCR. This showed that the amount of amplifiable DNA remained the same after the reaction (cycle threshold 14.24 and 14.32 for starting material and product respectively). Hence the hydrogenolysis conditions are fully compatible with both multicycle DEL synthesis and both DNA‐encoding and decoding.

These results demonstrate a highly efficient method of hydrogenolysis and hydrogenation of DNA‐conjugated substrates. The hydrogenolysis of Cbz‐protected amines and reduction of nitro‐ and cyano‐ groups provide a versatile means of unmasking amines for subsequent derivatisation, which will allow the use of a huge range of substrates in DEL synthesis. Cleavage of benzyl ethers provides a similar protocol for the deprotection of hydroxyl groups. Extension of the methodology to reduction of aryl halides and alkenes/alkynes may also be desirable in DEL synthesis as a means of removing these less desirable functional groups that may arise in some synthetic sequences. This methodology provides a valuable addition to the current range of transformations available in DEL synthesis.

## Conflict of interest

The authors declare no conflict of interest.

## Supporting information

As a service to our authors and readers, this journal provides supporting information supplied by the authors. Such materials are peer reviewed and may be re‐organized for online delivery, but are not copy‐edited or typeset. Technical support issues arising from supporting information (other than missing files) should be addressed to the authors.

Supporting Information

## Footnotes

n1 The reduction of benzaldehyde under the Pd(OAc)_2_/NaBH_4_ conditions is noteworthy: in the literature report, it is claimed that these conditions do not reduce aldehydes, although the reported analytical data are not definitive.
