# Supplementary material for: On‐DNA Transfer Hydrogenolysis and Hydrogenation for the Synthesis of DNA‐Encoded Chemical Libraries
Source: Angew Chem Int Ed Engl. 2021 Nov 27;61(3):e202111927. doi: 10.1002/anie.202111927 (PMC9298793; doi:10.1002/anie.202111927)

Angewandte Chemie
Eine Zeitschrift der Gesellschaft Deutscher Chemiker

Supporting Information

On-DNA Transfer Hydrogenolysis and Hydrogenation for the Synthesis of DNA-Encoded Chemical Libraries

*H. A. Stanway-Gordon, J. S. Graham, M. J. Waring**

Contents

Solvents and Reagents S2
Analytical Techniques S2
Chromatography and Equipment S2
General Procedures S3
General MMT-Deprotection Method S3
General HP Synthesis and Cleavage from solid support to form **1** S3
General Ethanol Precipitation Procedure for Purification of Intermediates and Products S4
Experimental Procedure and Characterisation of Amide Coupling to form **2**-**20, 22-26** S5
Results and Chromatograms S5
Cbz-Protected amines S7
Benzyl Ethers S11
Nitros S12
Halogens S15
Alkenes and Alkynes S17
Additional Functional Groups S18
Experimental Procedure and Characterisation of Amide Coupling to form **21** S19
Experimental Procedure and Characterisation of Transfer Hydrogenation to form **27** to **51** S20
2% TPGS-750-M Procedure S20
3% TPGS-750-M Procedure S21
Results and Chromatograms S21
Cbz-Deprotection S23
Bn-Deprotection S27
Nitro Reductions S29
Dehalogenations S33
Multiple Bond Reductions S35
Reduction of Other Functional Groups S38
Experimental Procedure for Transfer Hydrogenation without TPGS-750-M S39
Experimental Procedure for Transfer Hydrogenation without shaking S40
Comparison to Literature Reaction S40
Application of Procedure in Construction of Dipeptide **52** S41
Construction of 1x1 Library S43
Quantitation of DNA by qPCR S49

Solvents and Reagents

Chemicals were purchased from Fluorochem, Sigma-Aldrich and TCI, and used without further purification. Fmoc-NH-PEG4-COOH linker was purchased from Key Organics. TPGS-750-M was purchased from Sigma-Aldrich, concentrations of surfactant in water are quoted as percentages (by weight) as used by the supplier. All water used with DNA substrates was nuclease-free water purchased from ThermoFisher. DNA was purchased from Sigma-Aldrich as either solid supported crude material, or supplied as single strands after desalting.

Analytical Techniques

Calculated exact masses were quoted from ChemDraw Professional 15.0. DNA mass spectra were measured on an Agilent 6550 QTOF in negative mode, using standard 3200 m/z maximum and 2GHz extended dynamic range. Drying gas temperature was at 260 °C at 12 l/min, sheath gas temperature was 400 °C at 12 l/min, nebuliser at 45 psig, VCap voltage of 4000 V and nozzle voltage of 2000 V. 2 The LC was carried out on an Agilent 1260 Infinity 2 using an Agilent Advancedbio oligonucleotides column, 2.1x100 mm where the gradient was run at 0.8 ml/min from 10% MeOH to 50% MeOH over 4 mins against a 50 mM HFIP:15 mM DIPEA buffer solution. A 1 min flush at 95% MeOH preceded each run. Analysis of data was carried out using Agilent Qualitative Analysis version 7. Where appropriate a 1260 Infinity II Multiple Wavelength Detector was used and analysis was carried out at 260 nm.

The conversions were determined by integrating the peak areas in the total ion count chromatograms for the starting material and desired product and reported as a percentage. In cases where there were additional by-products formed the peak area for the desired product and those of all detectable products were used to determine the percentage of desired product relative to all detected components.

Gel electrophoresis was conducted using prepacked 4% E-Gel™ EX Agarose Gels on an Invitrogen™ E-Gel Power Snap Electrophoresis System, using Invitrogen™ Ultra Low Range DNA Ladders.

DNA concentrations were calculated using a NanoDrop™ One/OneC Microvolume UV-Vis Spectrophotometer, pipetting 1 µl of sample on the loading plate.

Chromatography and Equipment

Preparative HPLC purification was carried on an Agilent 1260 infinity system using a Phenomenex Clarity 5 um Oligo-RP column, 21.2x250 mm, with a gradient run at 20 ml/min from 10% MeOH to 60% MeOH over 11 mins against a 200 mM HFIP:8 mM TEA buffer solution. Fractions were analysed at 260 nm wavelength.

PCR, ligation and phosphorylation were carried out using a Techne® Prime thermal cycler (5PRIMEG/02). qPCR was carried out using a Bio-Rad CFX96™ real time system.

General Procedures

General MMT-Deprotection Method

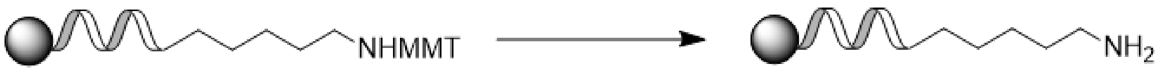

The average loading of single stranded DNA attached to solid support was found by cleavage from solid support using the below method and repeating three times. Nanodrop concentration of cleaved DNA showed that 103 mg yielded 2 µmol of DNA.

The single-stranded DNA employed was a 14mer (GTCTTGCCGAATTC) modified with a 5’ MMT-amino C6 linker, bound to solid support at the 3’ end. Solid supported DNA (103 mg, ca. 2 µmol) was washed with 3% trichloroacetic acid in DCM (10 x 500 µL). A yellow colour indicated that the deprotection was in progress. Once this colour subsided, the solid supported DNA was washed with DCM (3 x 500 µL) and left to air dry for 20 minutes before coupling to the headpiece.

General HP Synthesis and Cleavage from solid support to form **1**

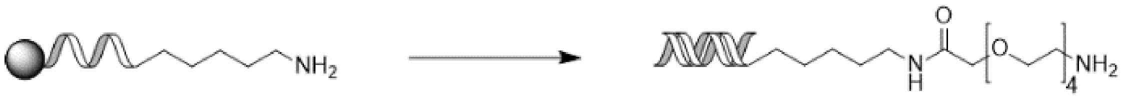

1

To a 1.5 mL microcentrifuge tube was added HATU (17 mg, 44 µmol), DIPEA (17 µL, 100 µmol) and DMF (1 mL). To this was added 12-((((9H-fluoren-9-yl)methoxy)carbonyl)amino)dodecanoic acid (26 mg, 40 µmol), and the mixture was shaken for 20 minutes at room temperature. Deprotected solid supported DNA (ca. 2 µmol) was added, and the reaction was shaken at room temperature overnight. The mixture was then filtered and washed with DMF (3 x 500 µL), MeCN (3 x 500 µL), MeOH (3 x 500 µL) and DCM (3 x 500 µL), before being allowed to air dry for 20 minutes.

40% methylamine in water (500 µL) and 33% ammonia in water (500 µL) were mixed in a 1.5 mL microcentrifuge tube. The solid supported DNA was added and the mixture shaken overnight at room temperature. The mixture was then filtered and washed with water (3 x 500 µL), and the filtrate was concentrated to ca. 0.5 mL using a Genevac at 40 °C. The crude product was then purified by HPLC, fractions concentrated using a Genevac at 40°C and dissolved in water (1 mL). The concentration of samples was then quantified by UV using a NanoDrop One by ThermoFisher. The usual amount was ca. 0.5-1 µmol of DNA after HPLC purification. The exact amount of the complimentary 14mer (GAATTCGGCAAGAC) was then added in water, and the solution was heated to 80 °C for 1 hour, then allowed to cool slowly. The double stranded DNA was concentrated using a Genevac at 40 °C until dry, and dissolved in water to form a 1 mM solution of the product.

Figure S1. Chromatogram of HP-**1**

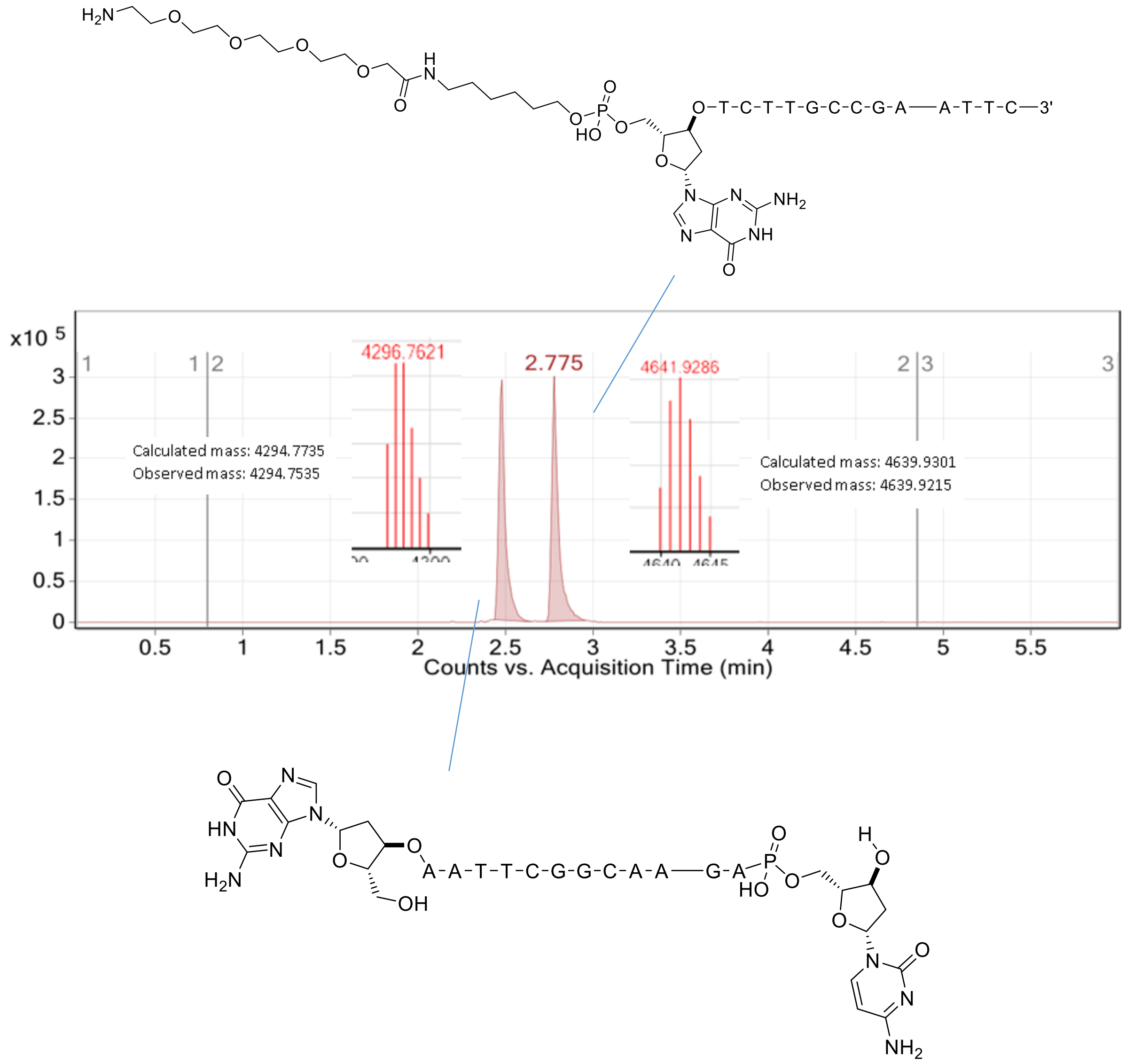

General Ethanol Precipitation Procedure for Purification of Intermediates and Products

To the reaction mixture was added 10% volume NaCl (5 M in water) and 3x volume cold EtOH. The mixture was allowed to sit for 1 hour at -78 °C, or overnight at -20 °C. The sample was then centrifuged at 13400 rpm for 10 minutes. The supernatant was decanted, and cold 70% EtOH was added. The mixture was centrifuged at 13400 rpm for a further 10 minutes and the supernatant was again decanted. The resulting pellet was allowed to air dry before being dissolved in water.

Experimental Procedure and Characterisation of Amide Coupling to form **2-20, 22-26**

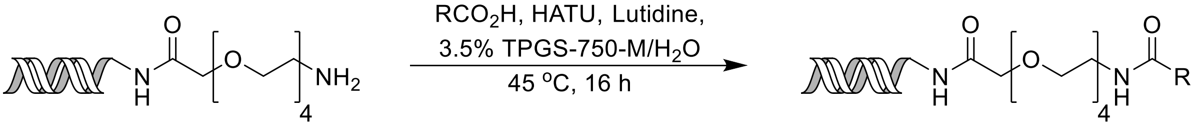

HATU (5.7 mg, 15 µmol) and the appropriate carboxylic acid (15 µmol) were added to a 50 µL glass insert for a Para-dox™ 96-well micro photoredox plate. 5% TPGS-750-M (21 µL), H_2O (4 µL) and HP **1** (5 µL, 1 mM in H_2O) were added to the vial, followed by lutidine (6.92 µL, 60 µmol). Samples were vortexed for 30 seconds each, and then heated in a Para-dox™ 96-well micro photoredox plate at 45 °C for 16 hours. Samples were then diluted to 200 µL with H_2O; DCM (2 x 400 µL) was added, and the samples were vortexed. The organic layer was discarded, the sample was filtered through a hydrophilic PTFE filter and analysed via mass spectrometry. Products were then precipitated according to the general ethanol precipitation procedure.

Results and Chromatograms

Table S1: Results of Amide Coupling to form **2-20, 22-26**

**No.**	R	Conversion (%)
2	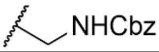	90
3	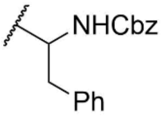	88
4	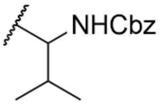	90
5	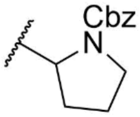	100
6	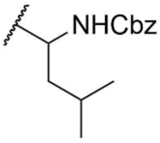	87
7	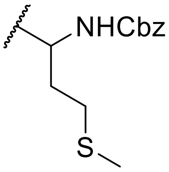	90
8	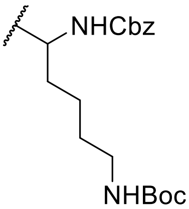	85
9	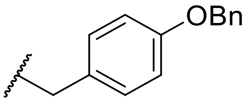	91

10	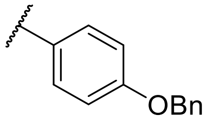	100
11	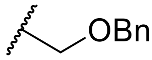	92
12	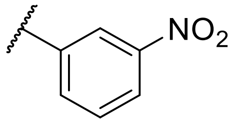	100
13	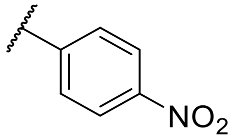	90
14	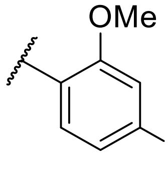	80
15	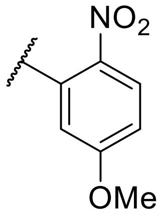	90
16	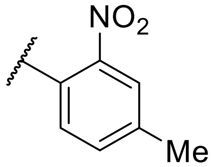	90
17	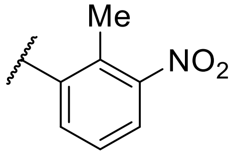	100
18	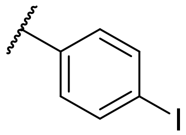	95
19	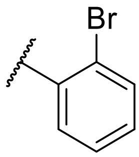	98
20	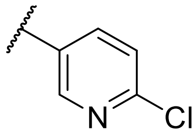	90
22	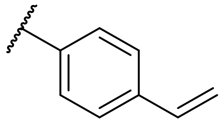	100

23	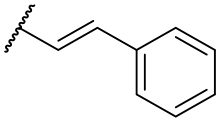	100
24	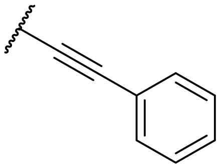	100
25	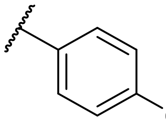	89
26	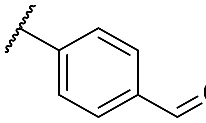	93

Cbz-Protected amines

*Figure S2: Chromatogram and deconvoluted mass spectrum of **2***

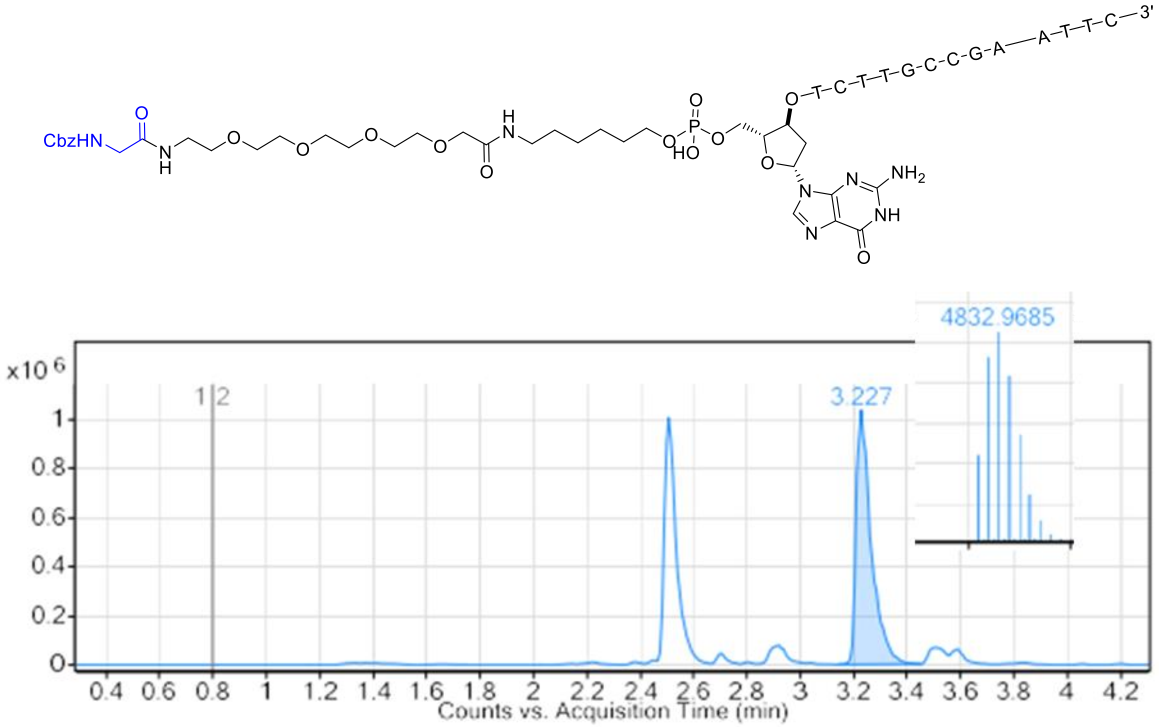

Calculated mass: 4830.9884

Observed mass: 4830.9764

Figure S3: Chromatogram and deconvoluted mass spectrum of ***3***

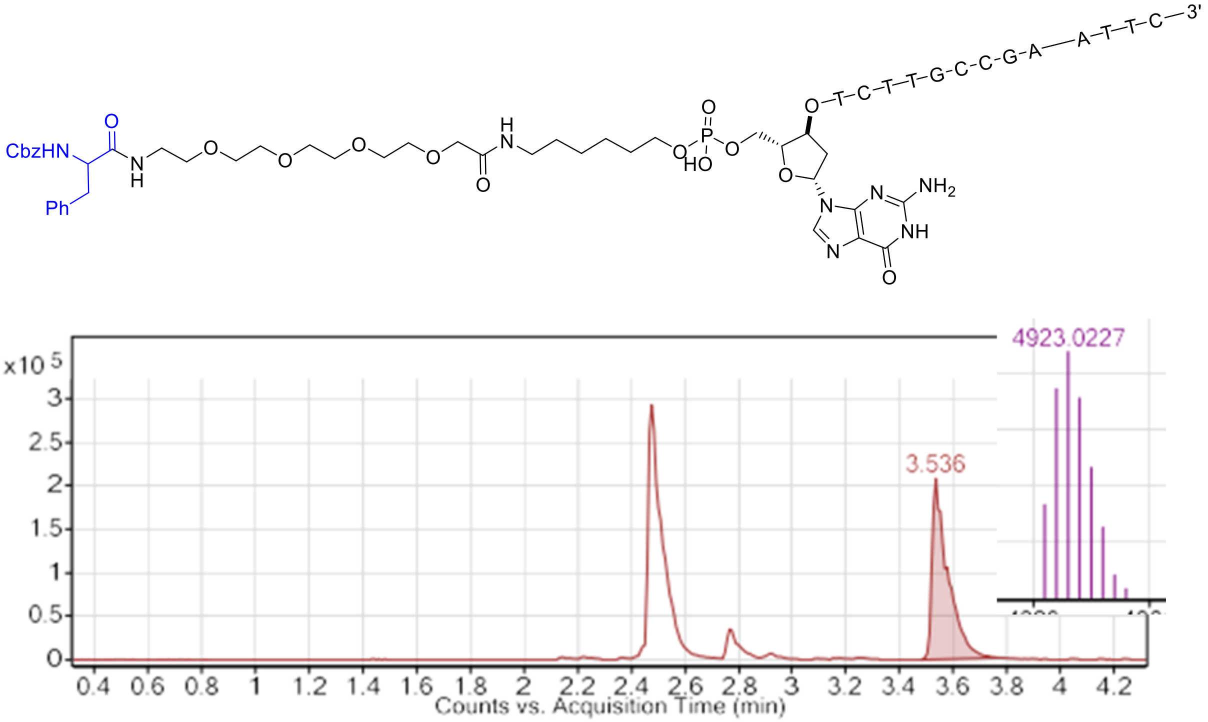

Calculated mass: 4921.0353
Observed mass: 4921.0150

Figure S4: Chromatogram and deconvoluted mass spectrum of ***4***

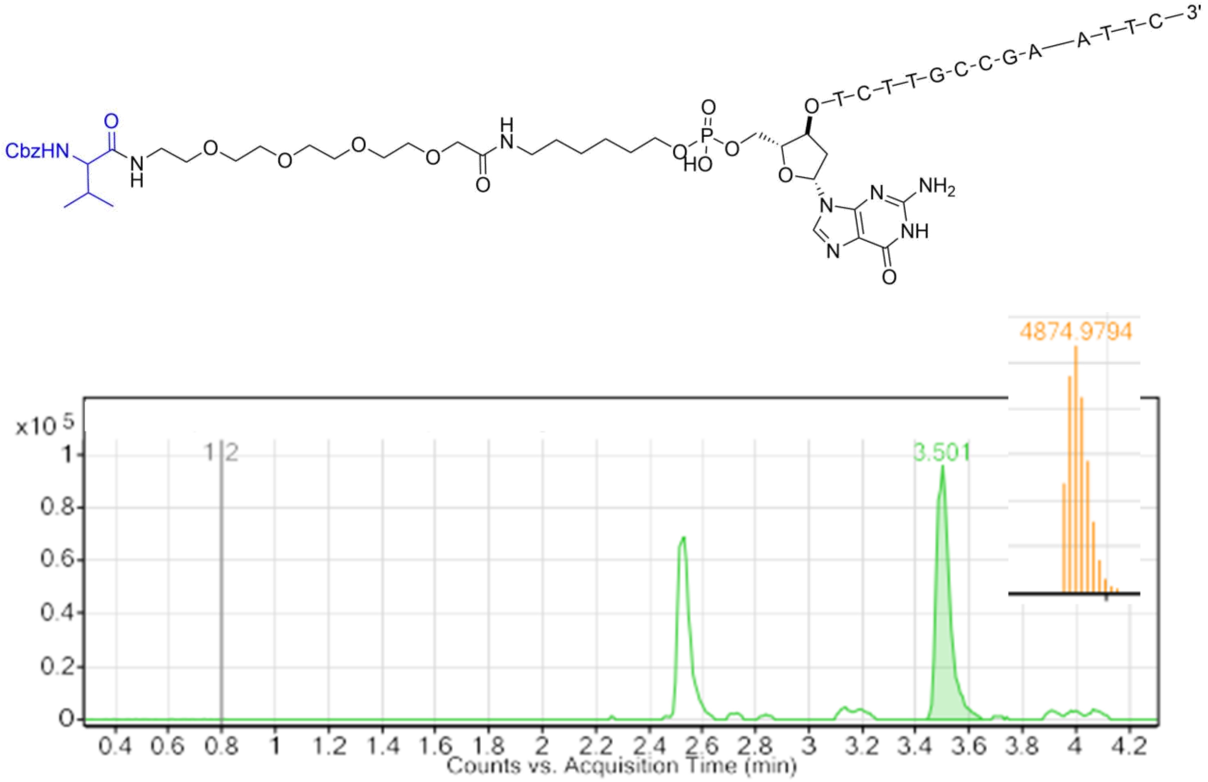

Calculated mass: 4873.0353
Observed mass: 4872.9705

*Figure S5: Chromatogram and deconvoluted mass spectrum of **5***

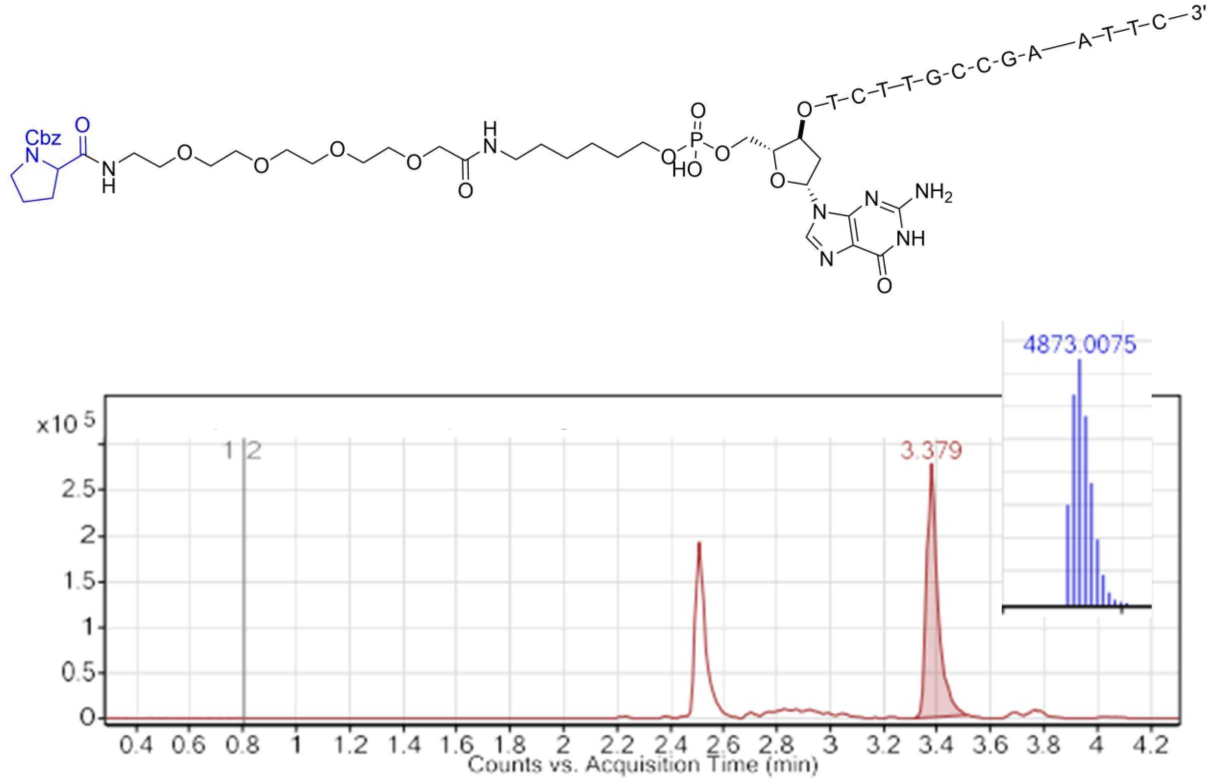

Calculated mass: 4871.0197

Observed mass: 4871.0007

*Figure S6: Chromatogram and deconvoluted mass spectrum of **6***

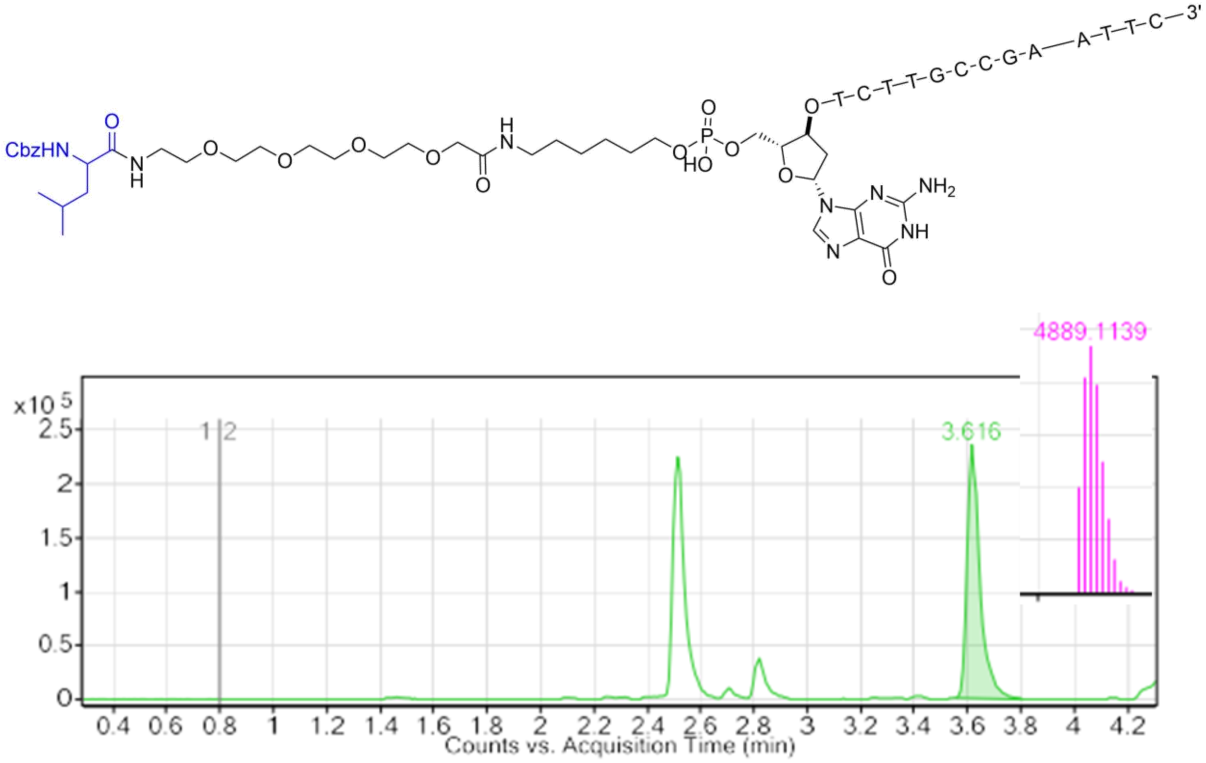

Calculated mass: 4887.0510

Observed mass: 4887.1074

Figure S7: Chromatogram and deconvoluted mass spectrum of ***7***

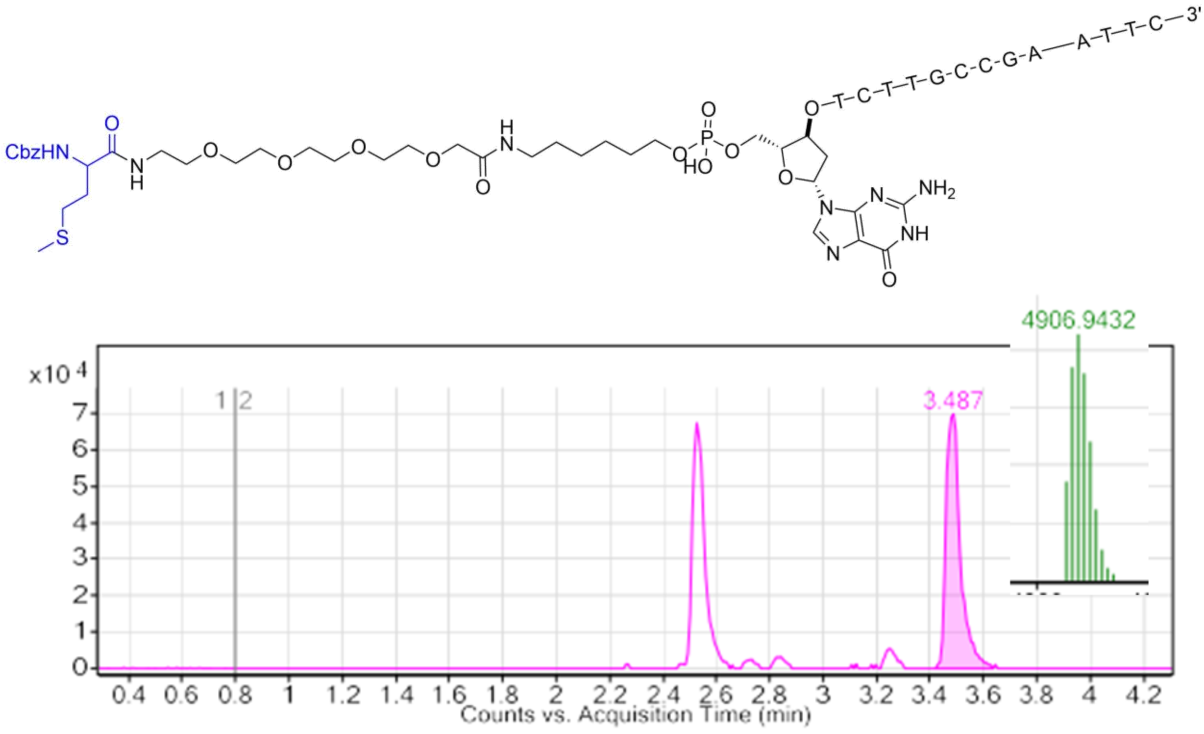

Calculated mass: 4905.0074
Observed mass: 4904.9364

Figure S8: Chromatogram and deconvoluted mass spectrum of ***8***

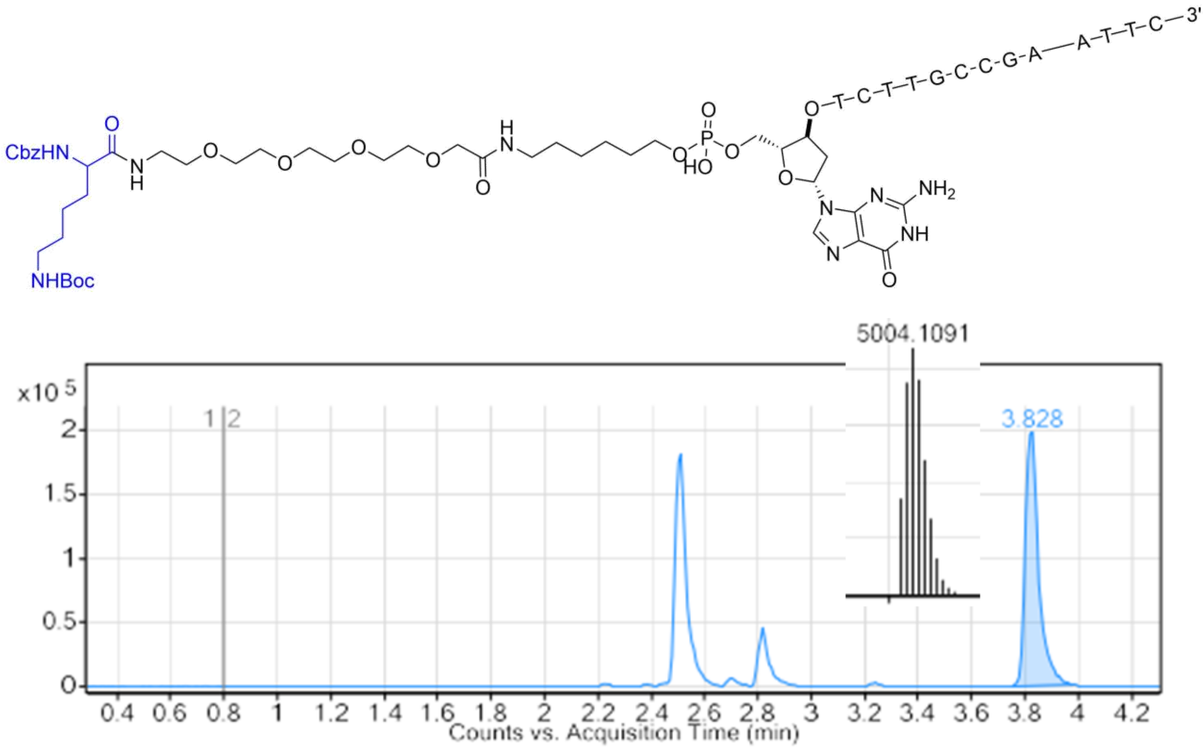

Calculated mass: 5002.1143
Observed mass: 5002.0968

Benzyl Ethers

*Figure S9: Chromatogram and deconvoluted mass spectrum of **9***

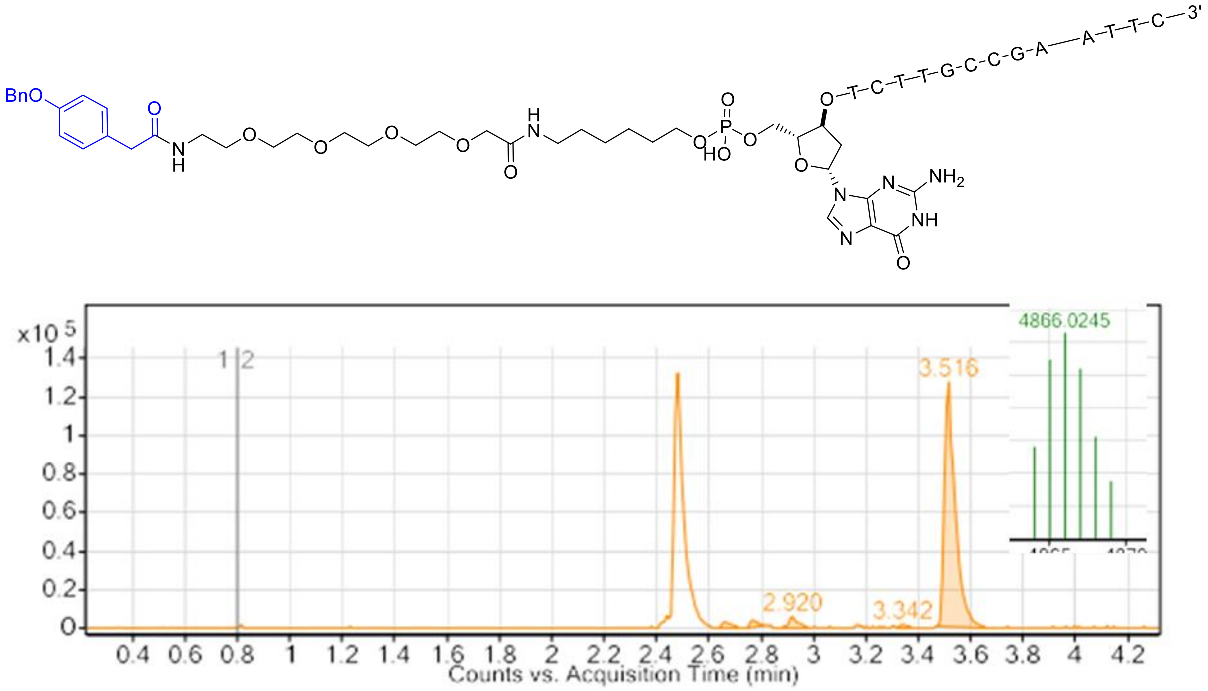

Calculated mass: 4864.0139
Observed mass:4864.0151

*Figure S10: Chromatogram and deconvoluted mass spectrum of **10***

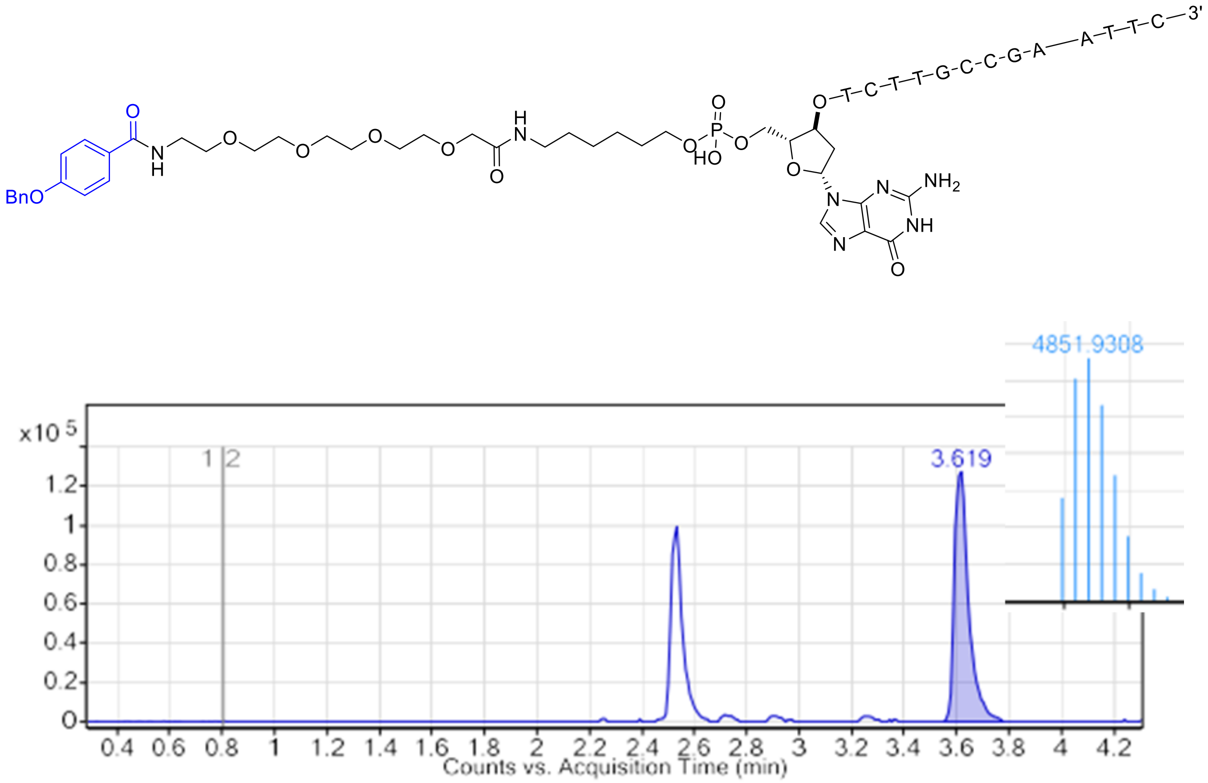

Calculated mass: 4849.9982
Observed mass: 4849.9238

Figure S11: Chromatogram and deconvoluted mass spectrum of ***11***

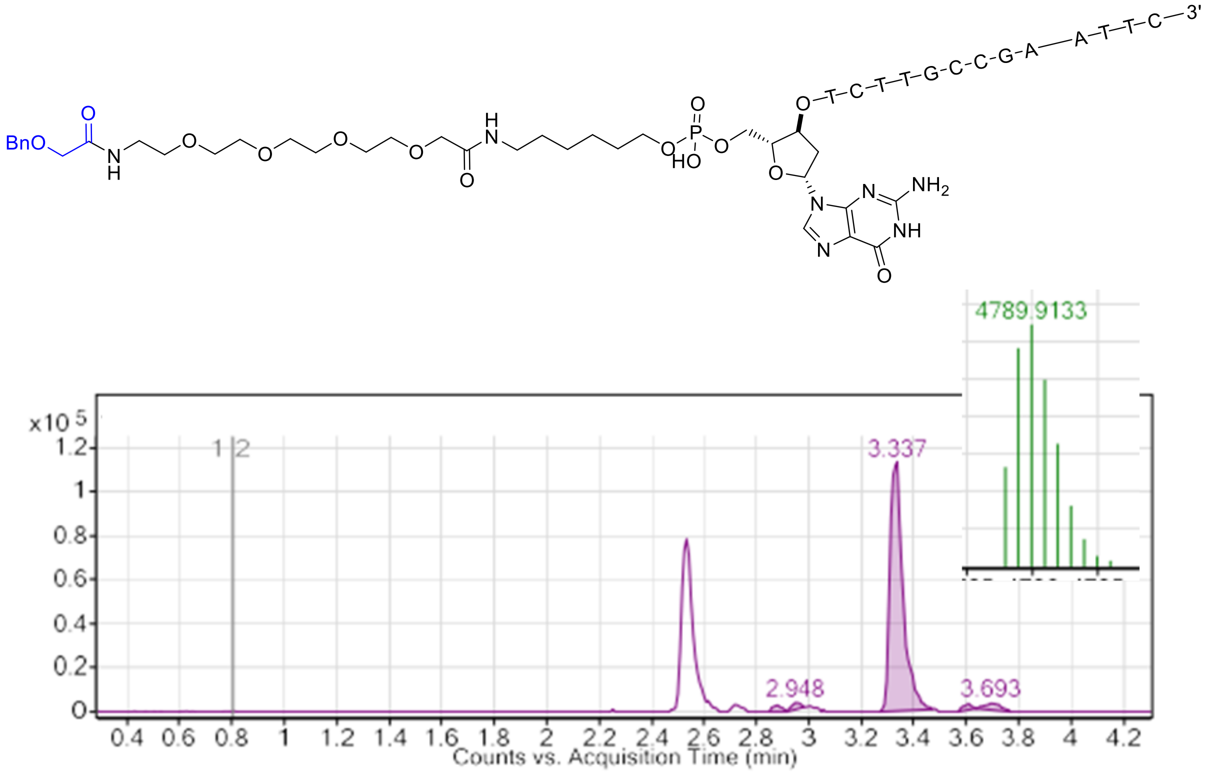

Calculated mass: 4787.9826
Observed mass: 4787.9085

Nitros

Figure S12: Chromatogram and deconvoluted mass spectrum of ***12***

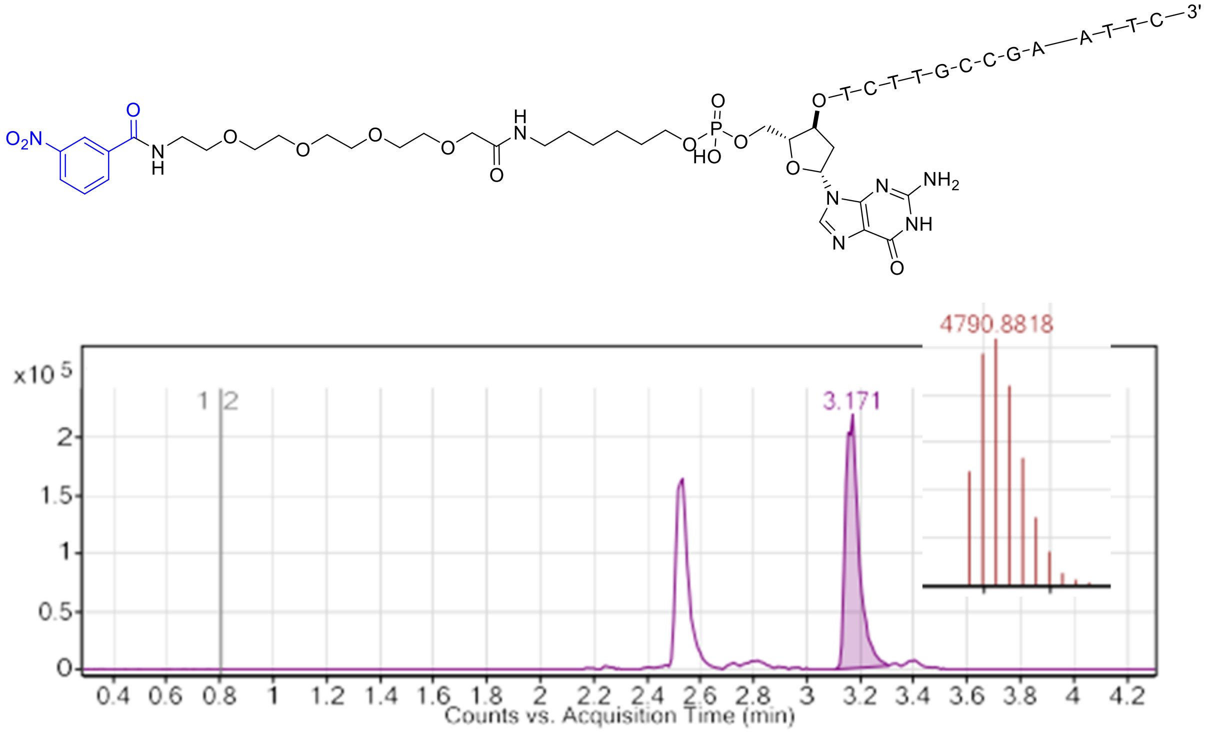

Calculated mass: 4788.9414
Observed mass: 4788.8724

*Figure S13: Chromatogram and deconvoluted mass spectrum of **13***

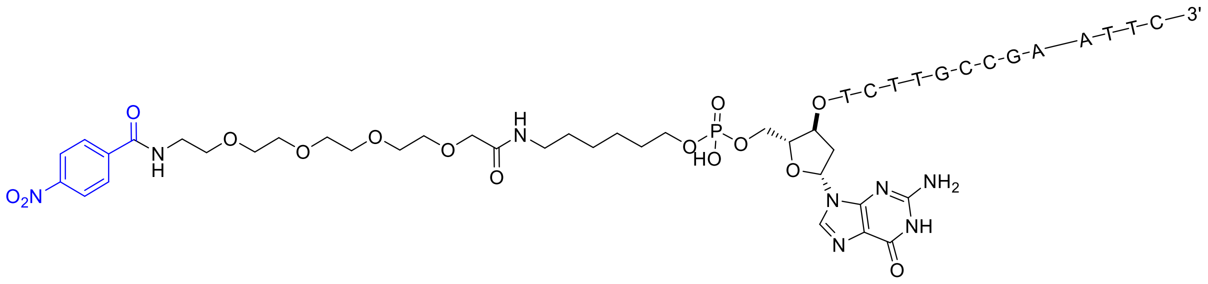

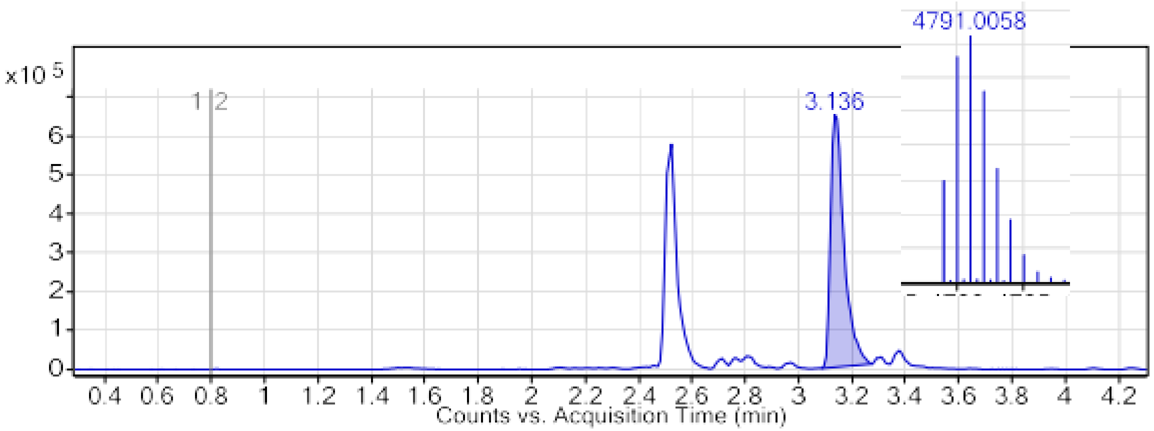

Calculated mass: 4788.9414
Observed mass: 4789.0042

*Figure S14: Chromatogram and deconvoluted mass spectrum of **14***

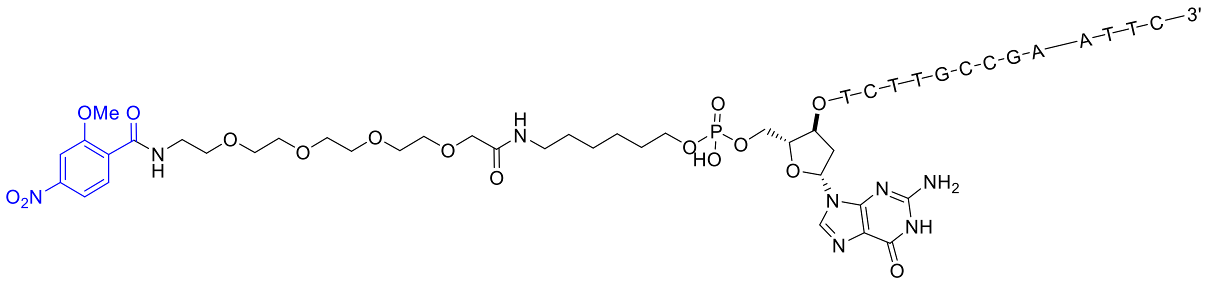

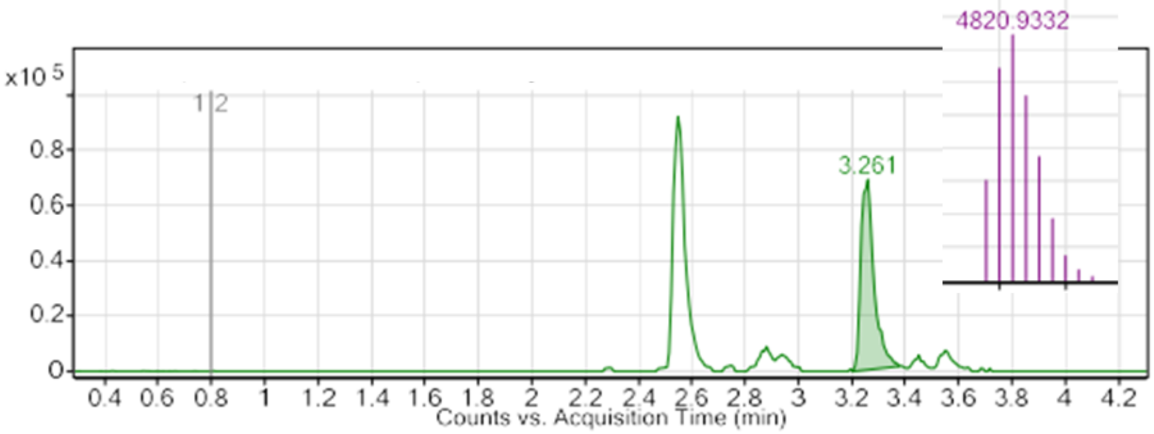

Calculated mass: 4818.9520
Observed mass: 4818.9283

*Figure S15: Chromatogram and deconvoluted mass spectrum of **15***

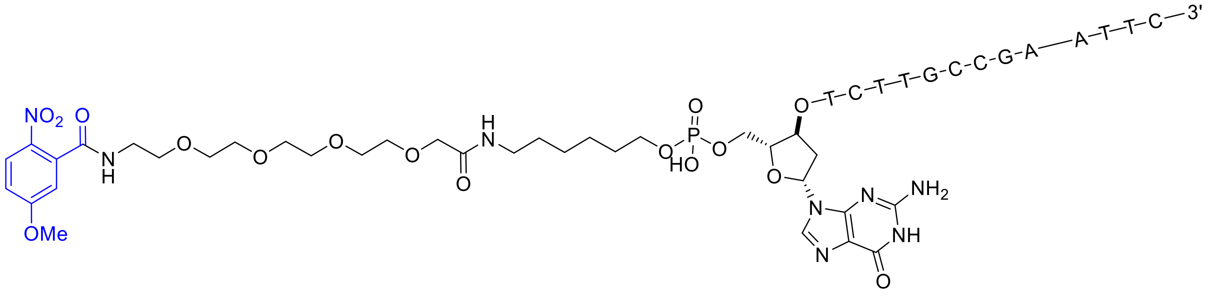

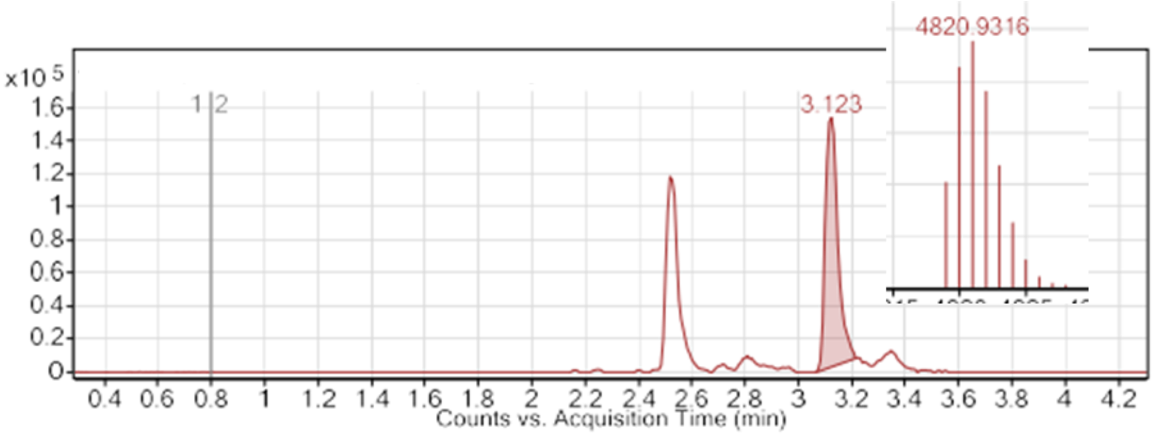

Calculated mass: 4818.9520
Observed mass: 4818.9267

*Figure S16: Chromatogram and deconvoluted mass spectrum of **16***

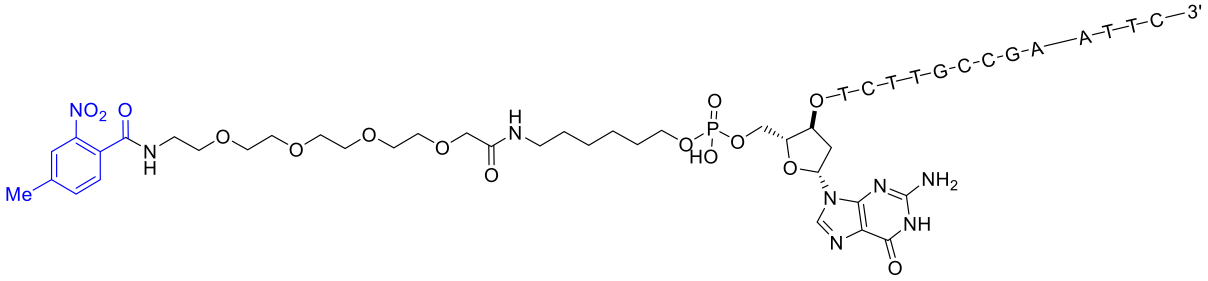

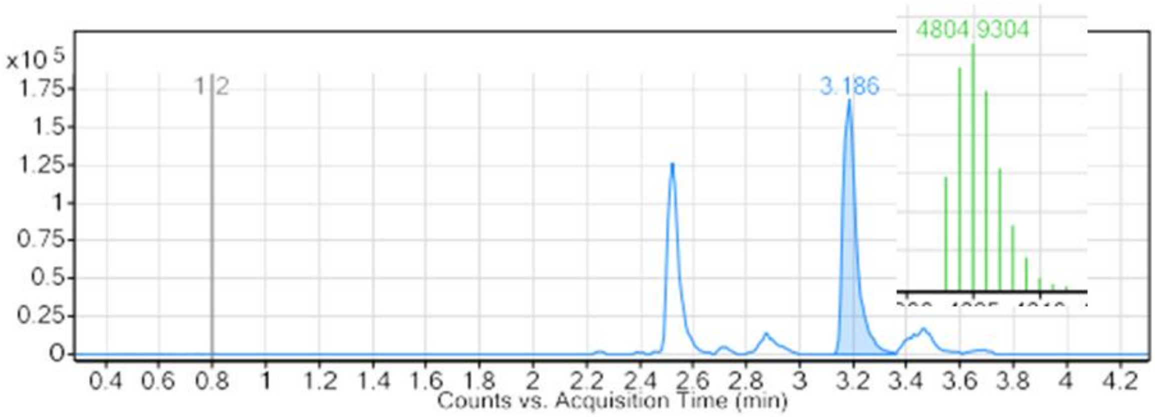

Calculated mass: 4802.9571
Observed mass: 4802.9177

Figure S17: Chromatogram and deconvoluted mass spectrum of ***17***

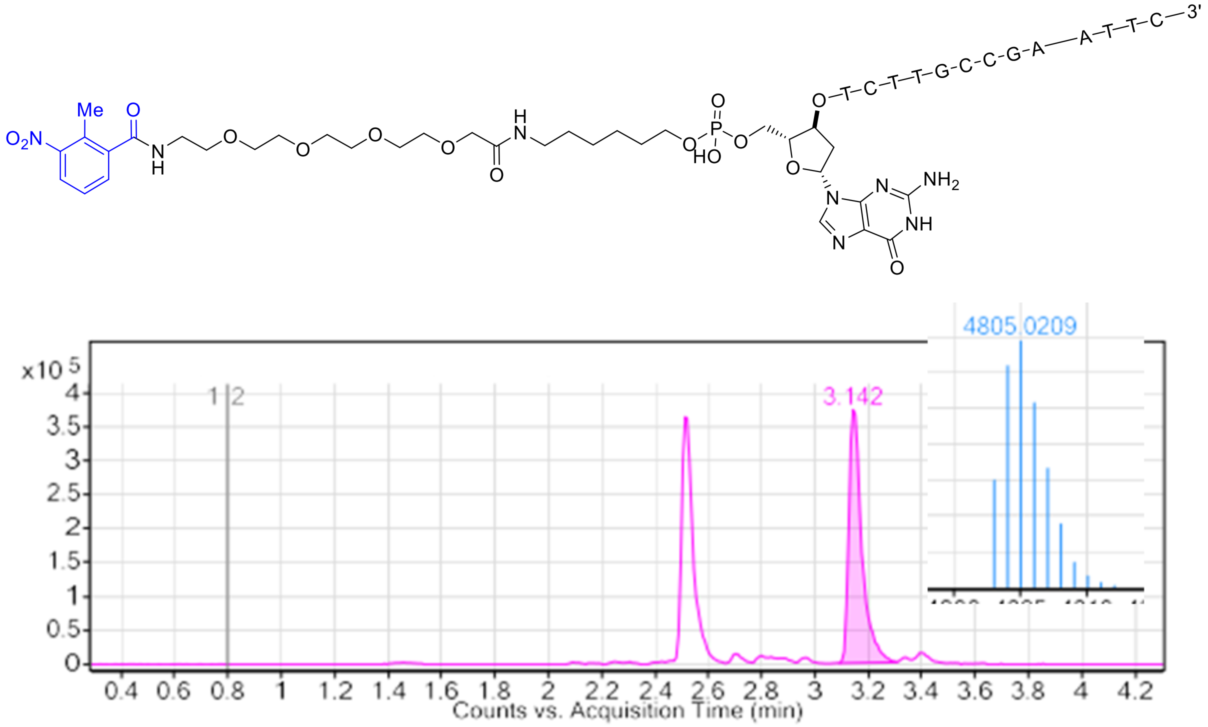

Calculated mass: 4802.9571

Observed mass: 4803.0232

Halogens

Figure S18: Chromatogram and deconvoluted mass spectrum of ***18***

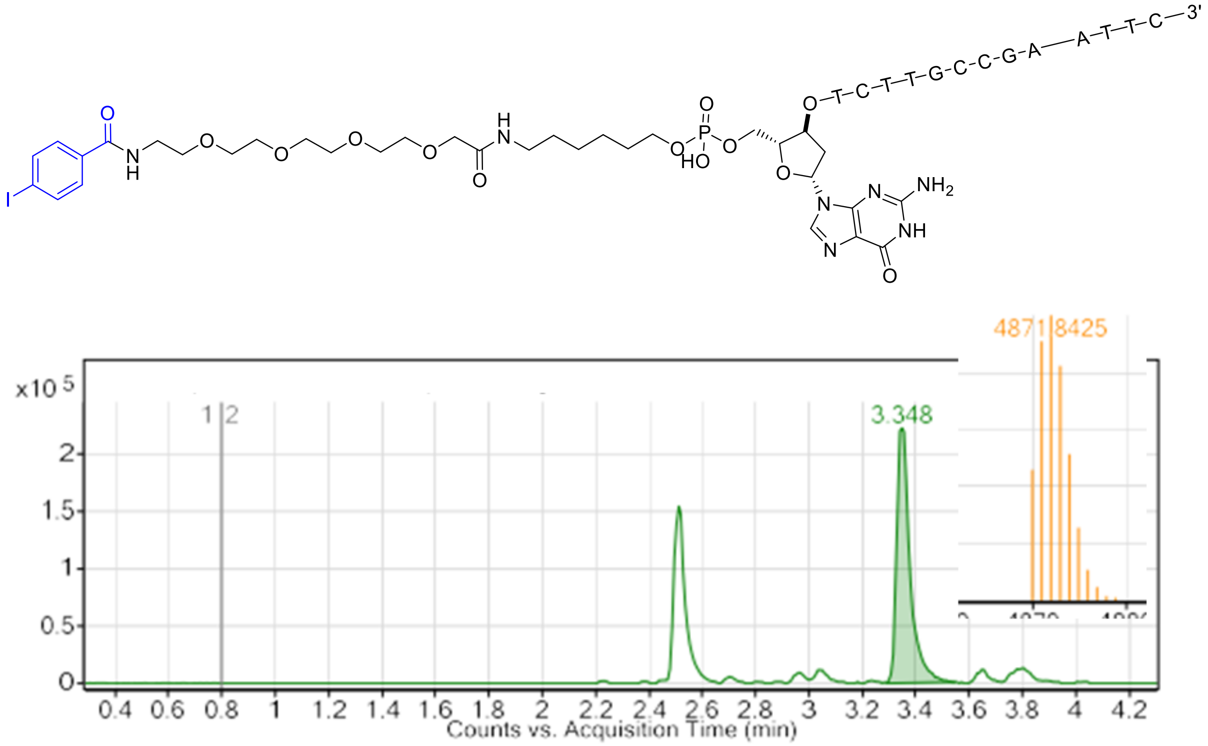

Calculated mass: 4869.8530

Found mass: 4869.8309

*Figure S19: Chromatogram and deconvoluted mass spectrum of **19***

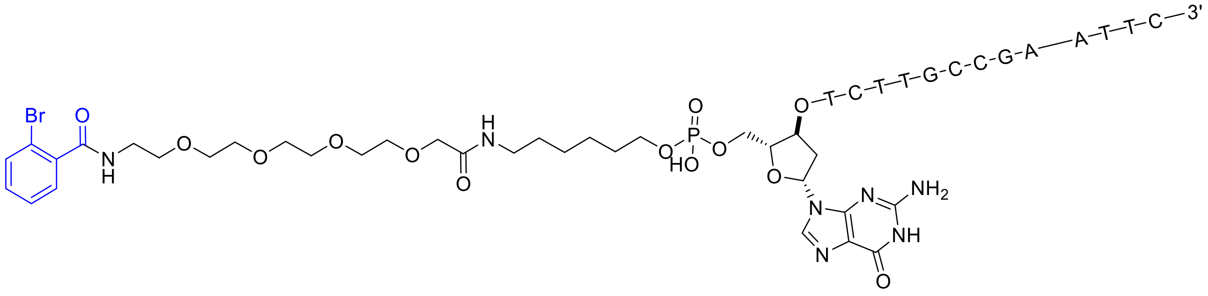

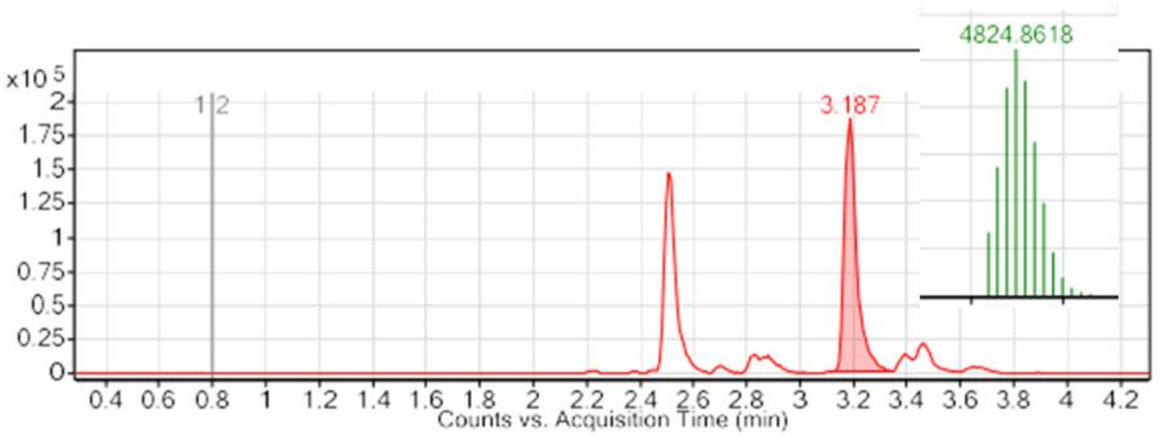

Calculated mass: 4821.8669
Observed mass: 4821.8464

*Figure S20: Chromatogram and deconvoluted mass spectrum of **20***

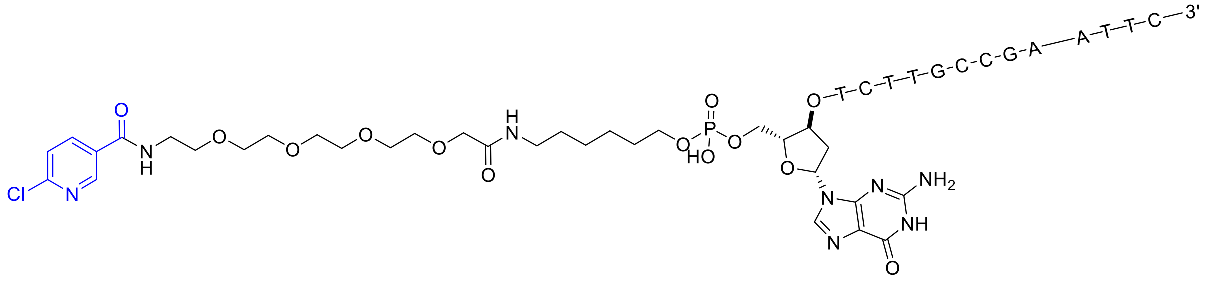

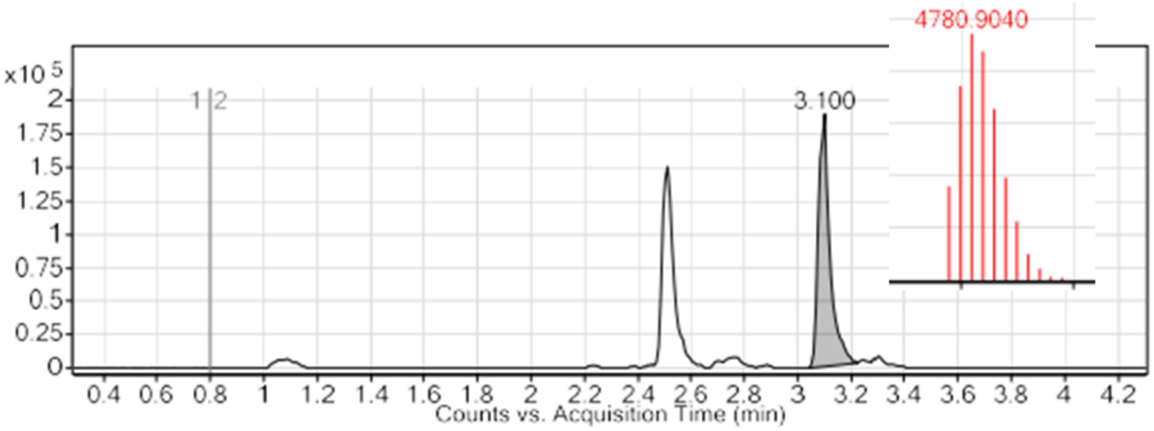

Calculated mass: 4778.9126
Observed mass: 4778.8961

Alkenes and Alkynes

Figure S21: Chromatogram and deconvoluted mass spectrum of ***22***

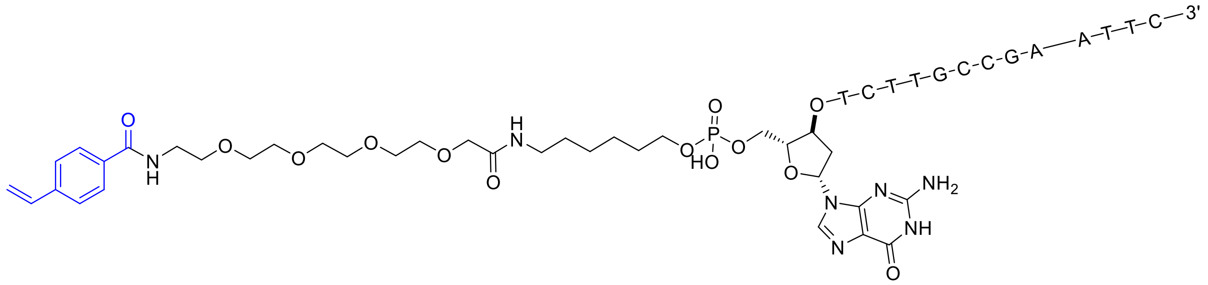

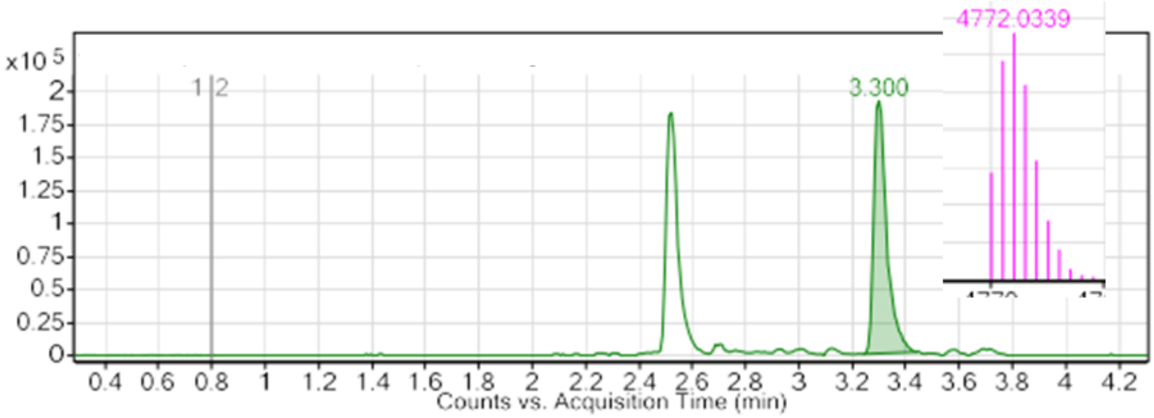

Calculated mass: 4769.9720

Observed mass: 4770.0277

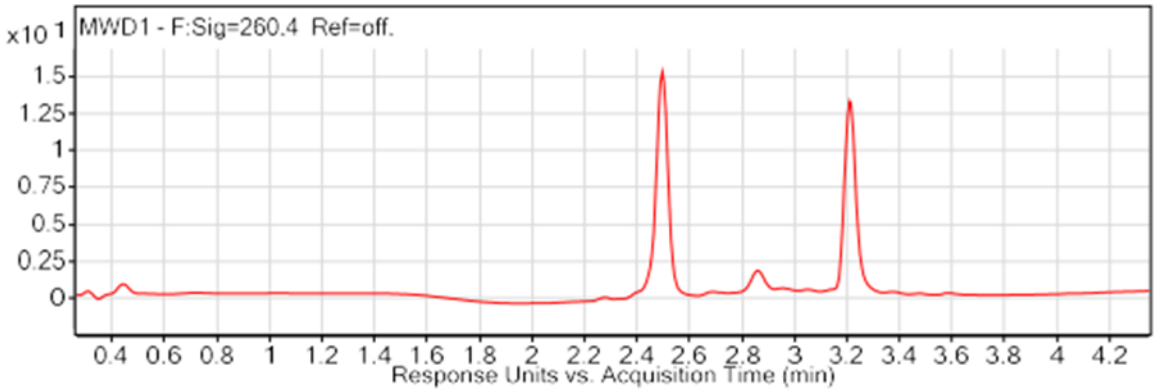

Figure S22: Chromatogram and deconvoluted mass spectrum of ***23***

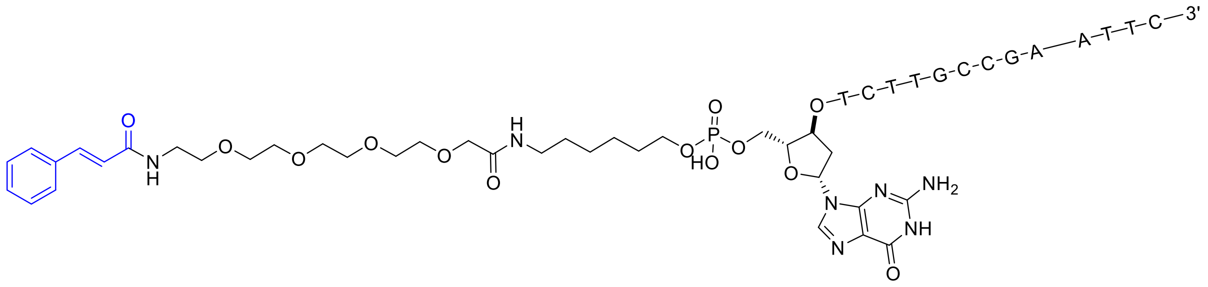

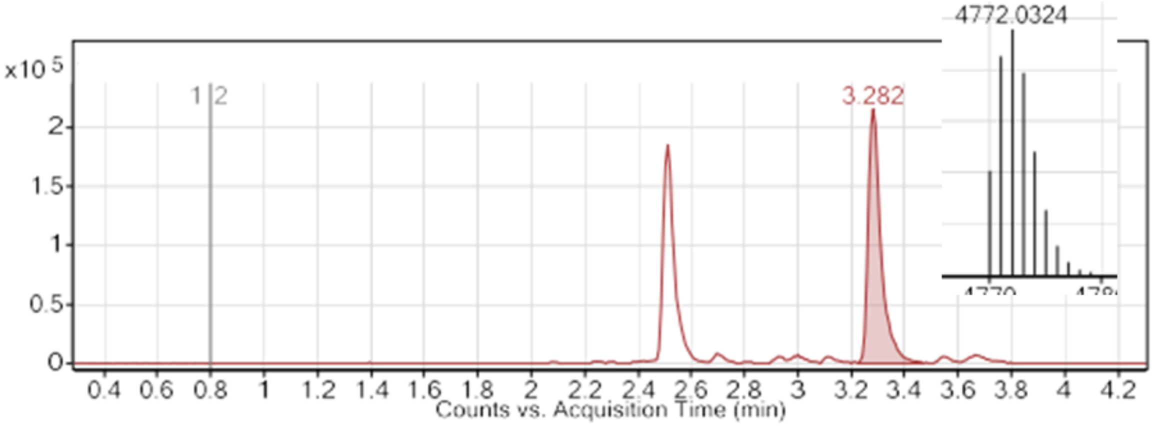

Calculated mass: 4769.9720
Observed mass:4770.0242

Figure S23: Chromatogram and deconvoluted mass spectrum of ***24***

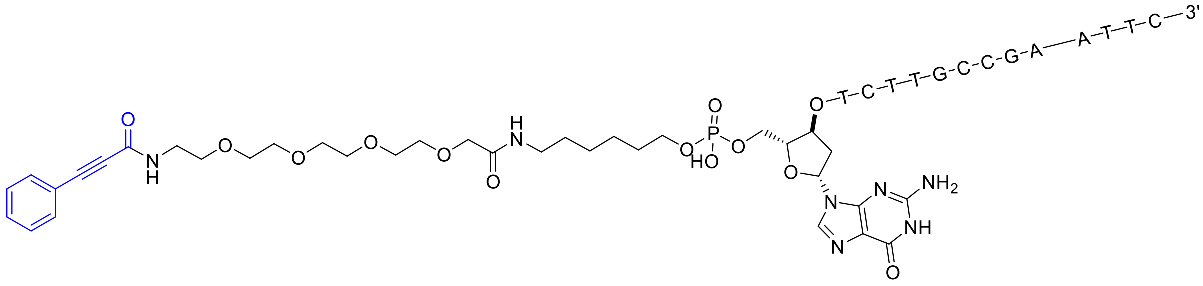

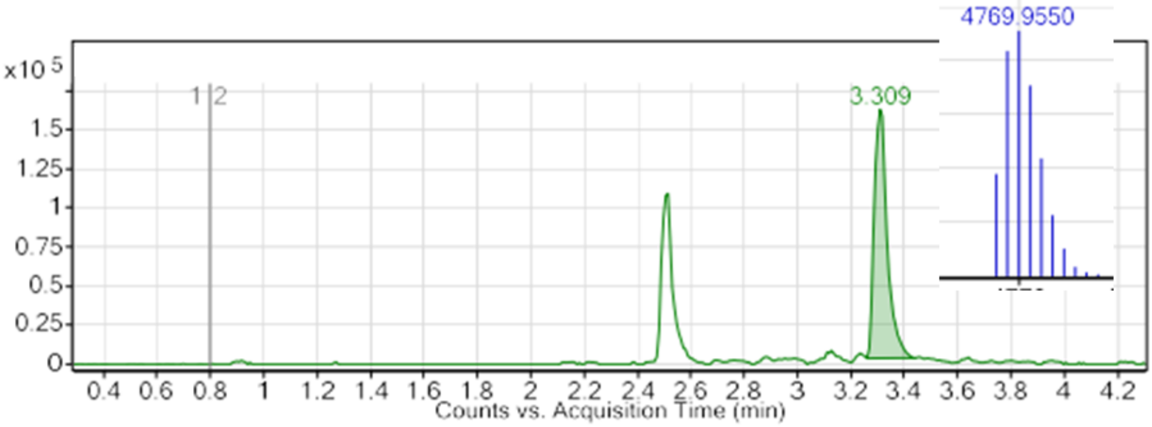

Calculated mass: 4767.9563
Observed mass: 4767.9461

Additional Functional Groups

Figure S24: Chromatogram and deconvoluted mass spectrum of ***25***

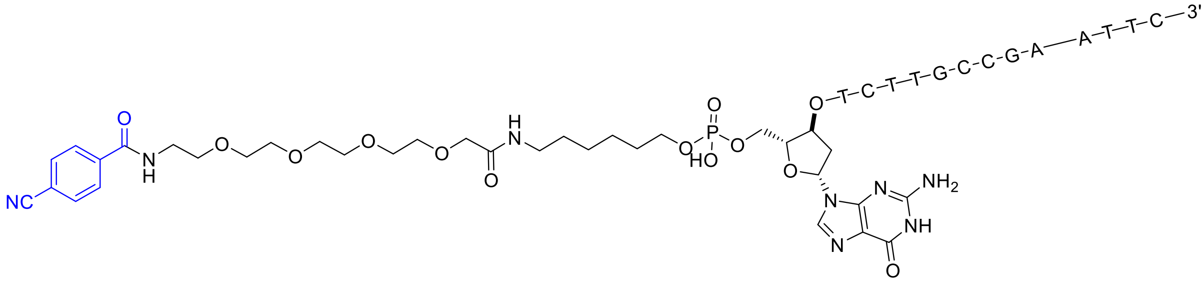

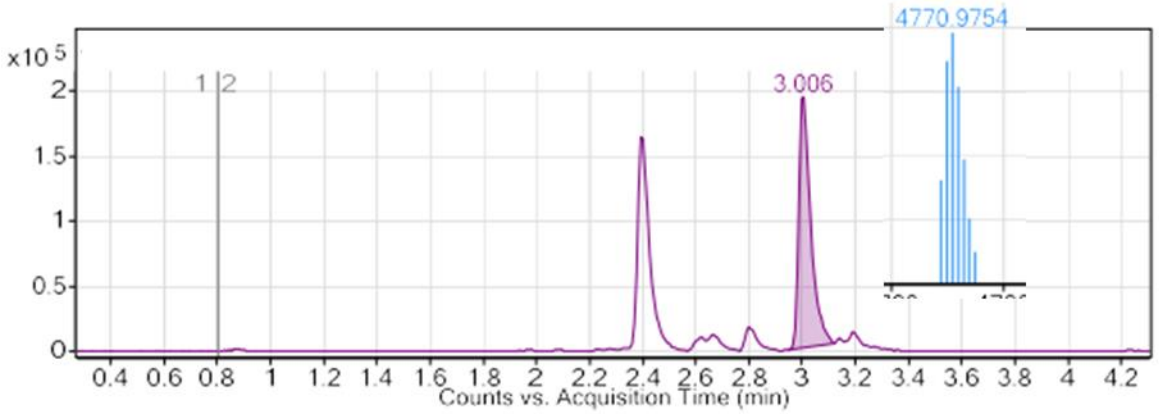

Calculated mass: 4768.9516
Observed mass: 4768.9684

*Figure S25: Chromatogram and deconvoluted mass spectrum of **26***

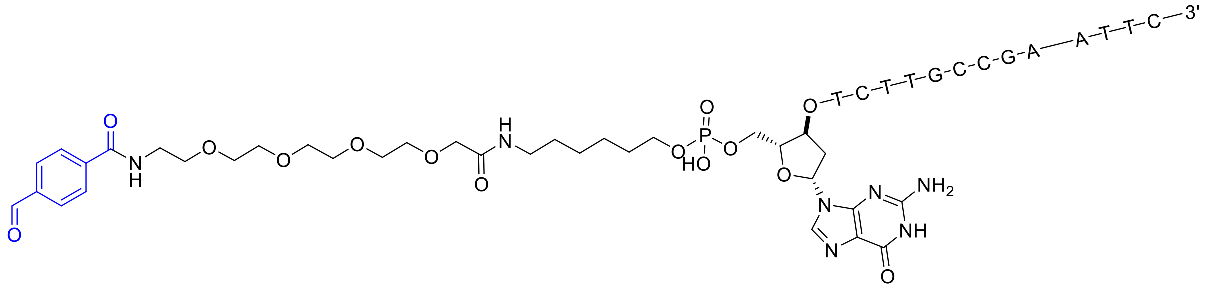

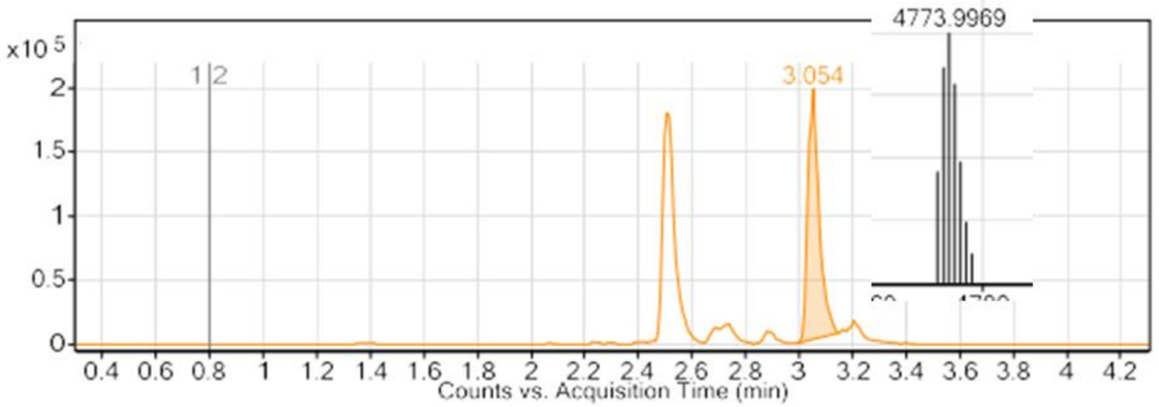

Calculated mass: 4771.9513
Observed mass: 4771.9887

Experimental Procedure and Characterisation of Amide Coupling to form 21

To a 200 µL PCR tube was added Headpiece **1** (20 µL, 1 mM in water), sodium borate buffer (20 µL, pH 9.4) and N-acryloxysuccinimide (8 µL, 200 mM in DMA). The solution was shaken at room temperature overnight. Samples were then diluted to 200 µL with H_2O, filtered through a hydrophilic PTFE filter and analysed via mass spectrometry (94% conversion to desired product). **21** was then precipitated according to the general ethanol precipitation procedure.

*Figure S26: Chromatogram and deconvoluted mass spectrum of **21***

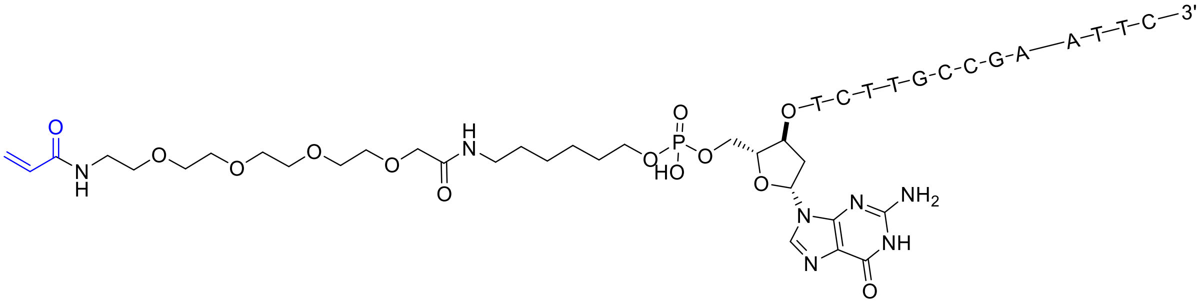

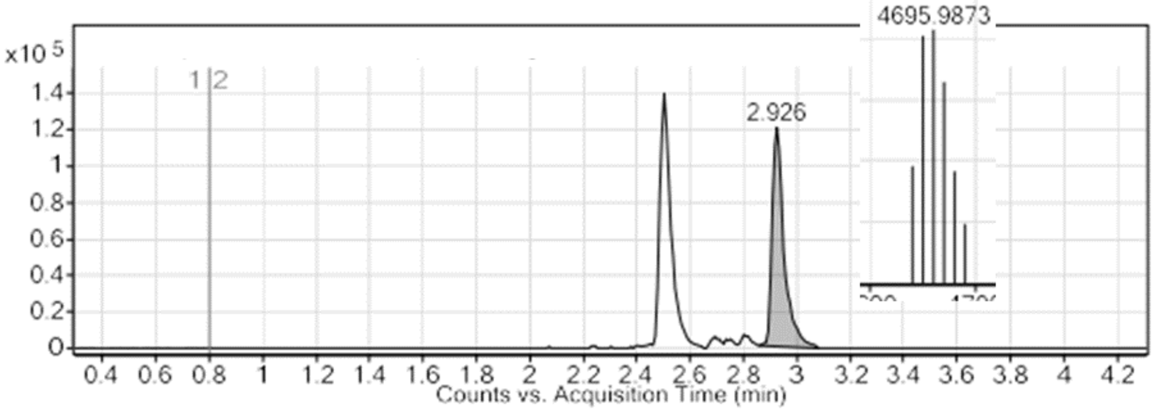

Calculated mass: 4393.9407
Observed mass: 4693.9760

Experimental Procedure and Characterisation of Transfer Hydrogenation to form **27** to **51**

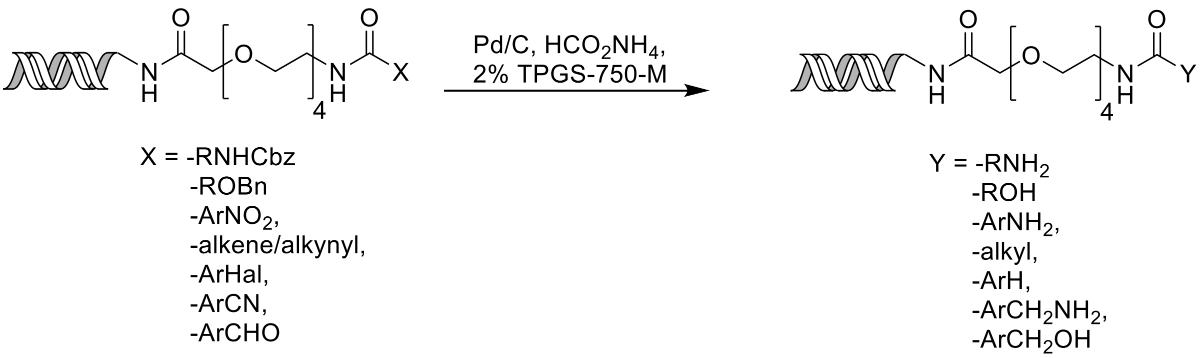

2% TPGS-750-M Procedure

To a 50 µL glass insert for a Para-dox™ 96-well micro photoredox plate was added 10% wt Pd/C (5 µL of 8 mg in 200 µL water), 5% TPGS-750-M (12 µL), water (6 µL) and DNA (4 µL, 0.25 mM in water). Samples were vortexed for 30 seconds each, then ammonium formate (3 µL, 5.3 M in water) was added, and the samples were vortexed for a further 10 seconds. Reactions were then shaken at 1200 rpm, at room temperature in a PMS-1000i Microplate shaker for up to 2 hours. The samples were diluted to 200 µL with water, filtered through a hydrophilic PTFE filter and analysed via mass spectrometry. Products were precipitated according to the general ethanol precipitation procedure.

3% TPGS-750-M Procedure

To a 50 µL glass insert for a Para-dox™ 96-well micro photoredox plate was added 10% wt Pd/C (5 µL of 8 mg in 200 µL water), 5% TPGS-750-M (18 µL) and DNA (4 µL, 0.25 mM in water). Samples were vortexed for 30 seconds each, then ammonium formate (3 µL, 5.3 M in water) was added, and the samples were vortexed for a further 10 seconds. Reactions were then shaken at 1200 rpm, at room temperature in a PMS-1000i Microplate shaker for up to 2 hours. The samples were diluted to 200 µL with water, filtered through a hydrophilic PTFE filter and analysed via mass spectrometry. Products were precipitated according to the general ethanol precipitation procedure.

Results and Chromatograms

Table S2: Results of Transfer Hydrogenation in TPGS-750-M

No.	Starting Material	Product	Conversion in 2% TPGS-750-M (%)	Conversion in 3% TPGS-750-M (%)
27	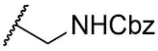	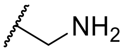	100	100
28	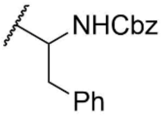	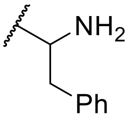	100	100
29	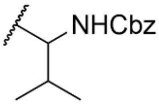	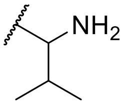	100	100
30	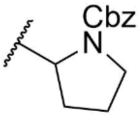	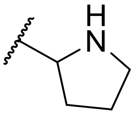	100	100
31	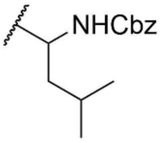	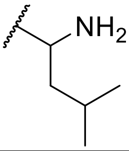	100	100
32	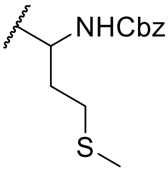	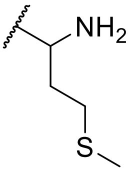	90	100 (94)
33	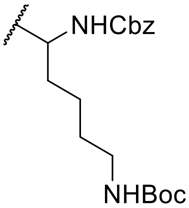	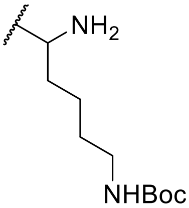	100	100
34	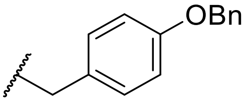	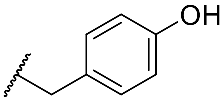	100	100
35	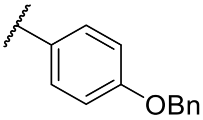	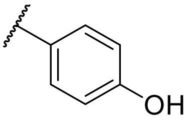	100	100
36	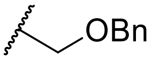	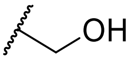	18	100

37	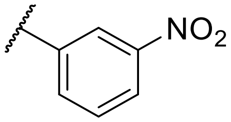	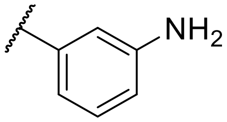	100	100
38	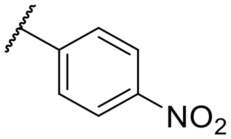	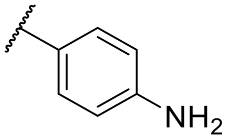	100	100
39	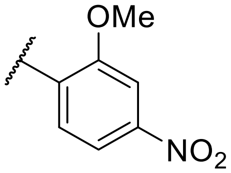	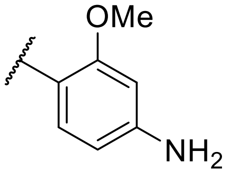	100	100
40	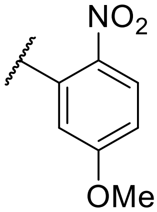	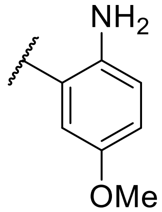	100	100
41	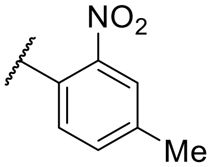	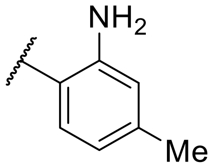	100	100
42	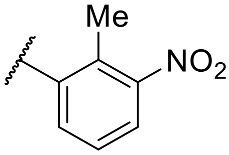	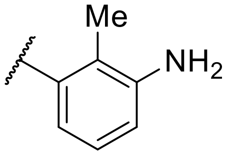	100	100
43	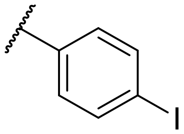	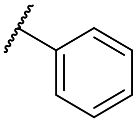	100	100
43	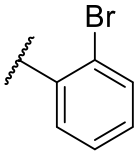	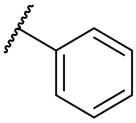	60	100
44	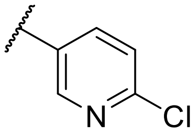	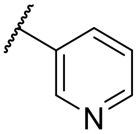	0	100 (96)
45	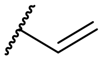	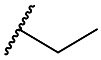	100	100
46	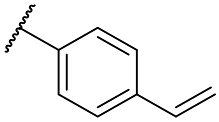	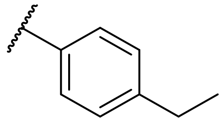	100	100
47	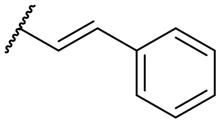	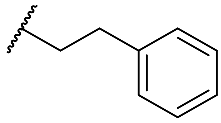	0	100

47	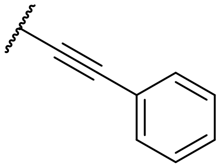	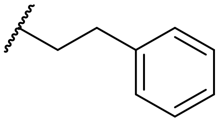	100	100
48	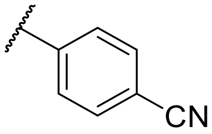	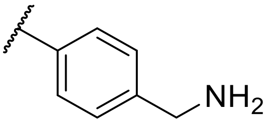	48 (39)	97 (67)
49	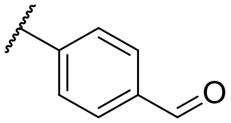	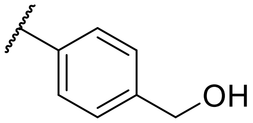	100 (91)	100 (94)

Cbz-Deprotection

*Figure S27: Chromatogram and deconvoluted mass spectrum of **27***

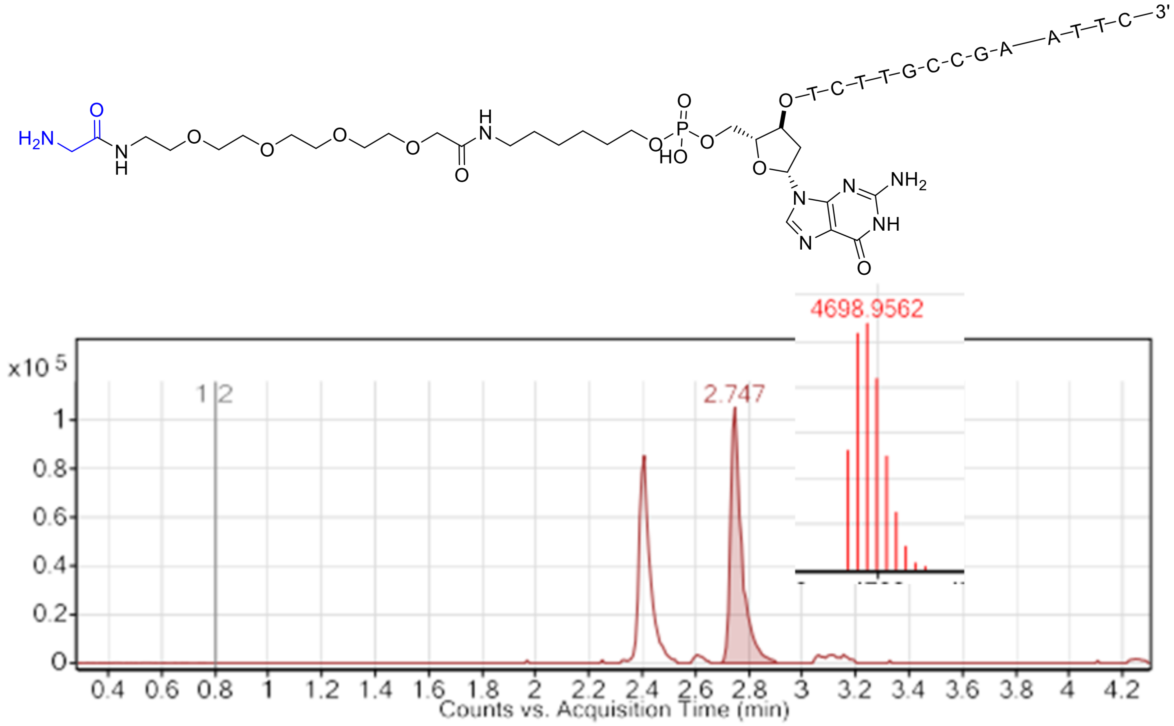

Calculated mass: 4696.9516
Observed mass: 4696.9505

*Figure S28: Chromatogram and deconvoluted mass spectrum of **28***

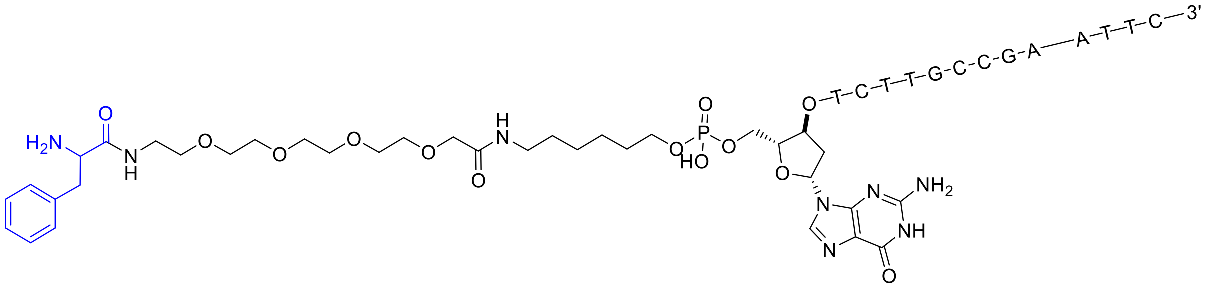

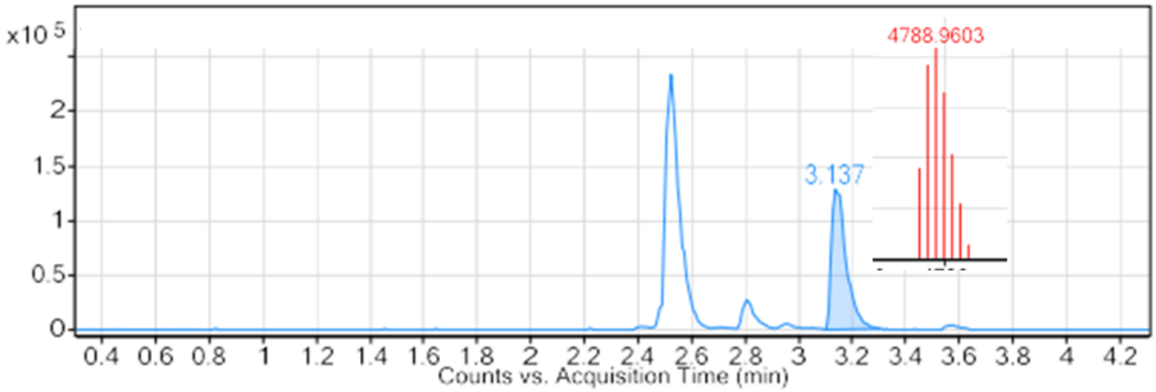

Calculated mass: 4786.9985
Observed mass: 4786.9524

*Figure S29: Chromatogram and deconvoluted mass spectrum of **29***

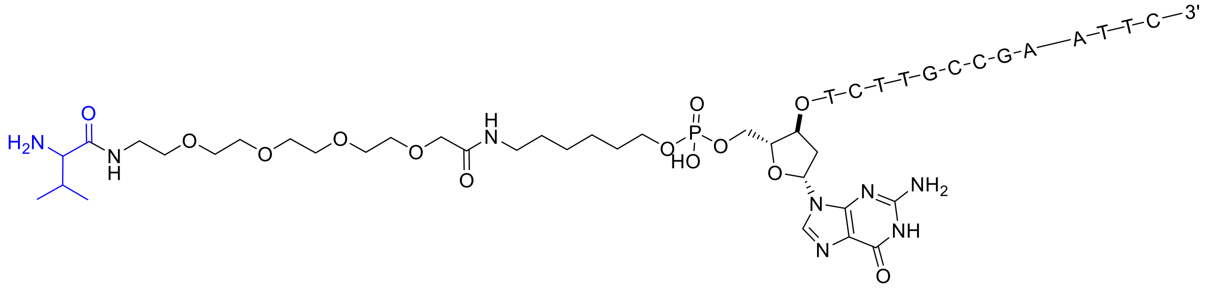

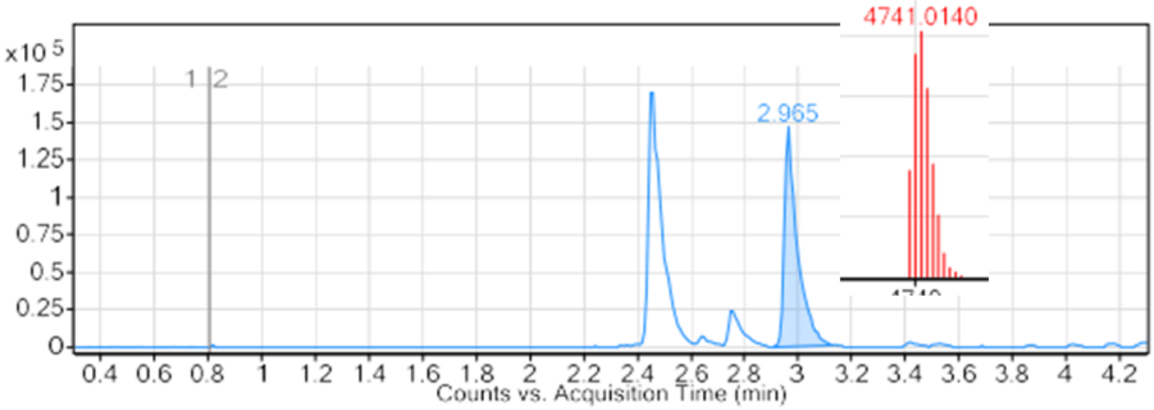

Calculated mass: 4738.9985
Observed mass: 4739.0054

Figure S30: Chromatogram and deconvoluted mass spectrum of ***30***

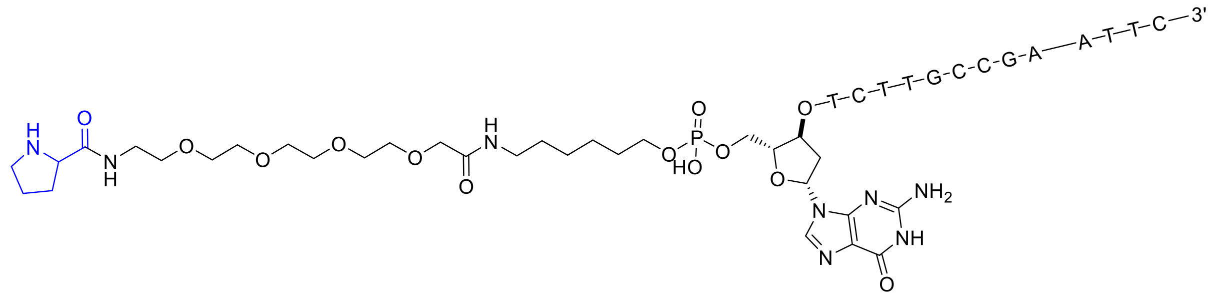

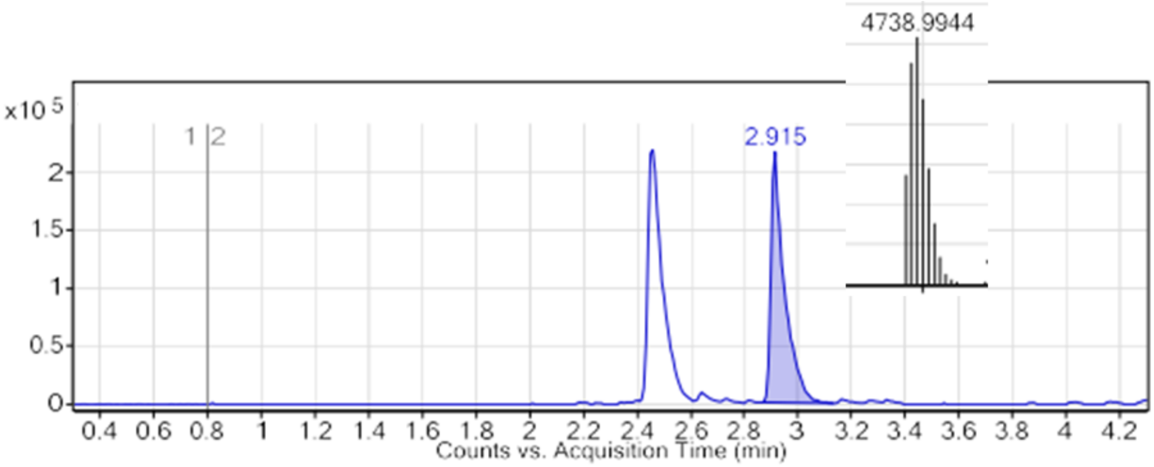

Calculated mass: 4736.9829
Observed mass: 4736.9860

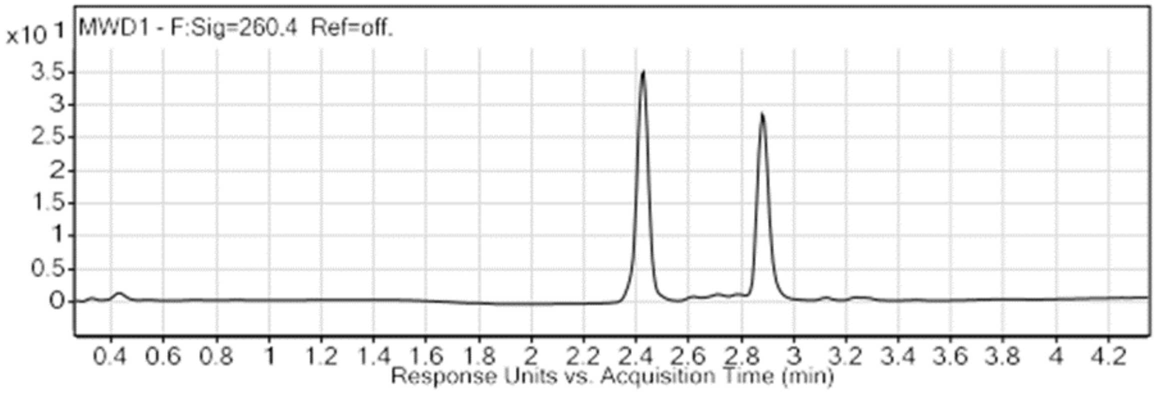

Figure S31: Chromatogram and deconvoluted mass spectrum of ***31***

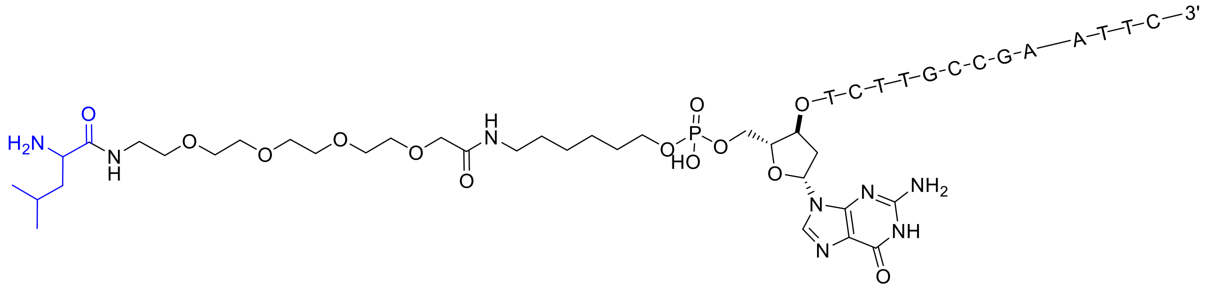

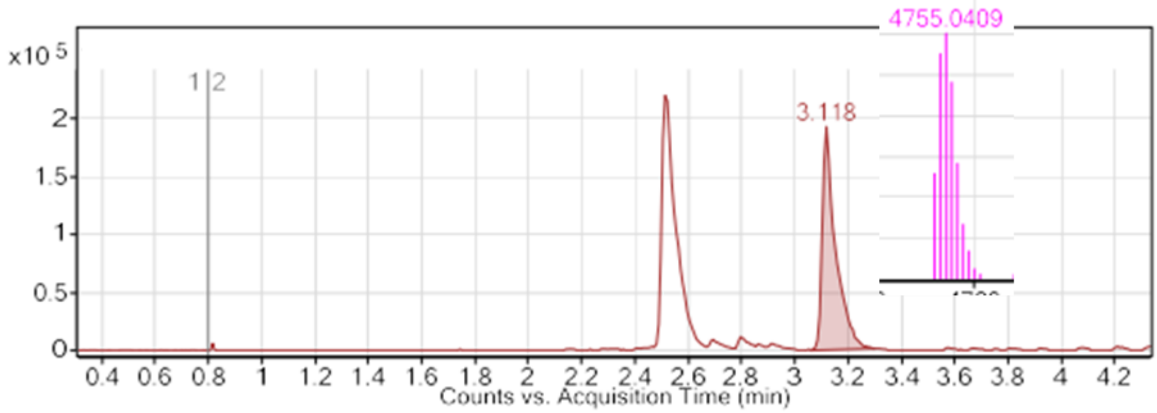

Calculated mass: 4753.0142
Observed mass:4753.0366

*Figure S32: Chromatogram and deconvoluted mass spectrum of **32***

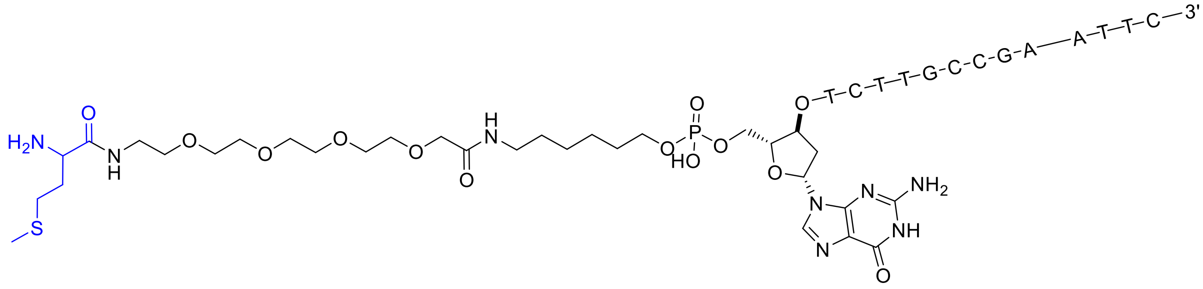

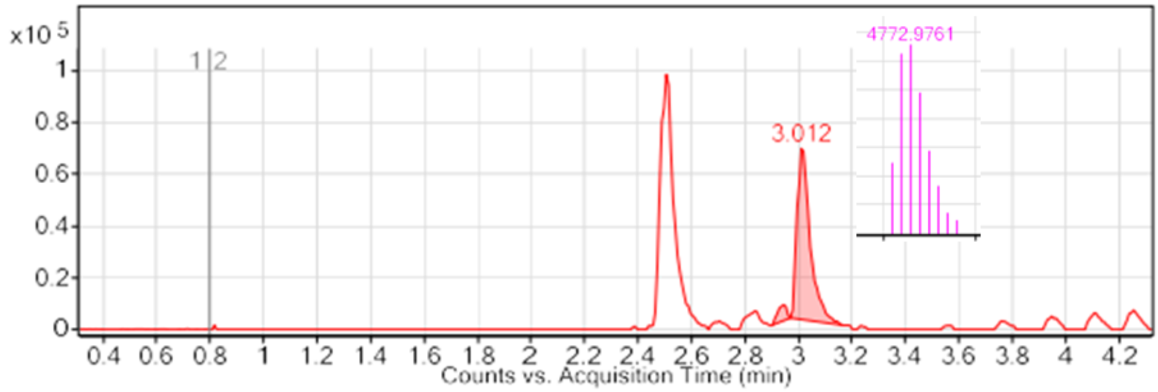

Calculated mass: 4770.9706
Observed mass: 4770.9546

*Figure S33: Chromatogram and deconvoluted mass spectrum of **33***

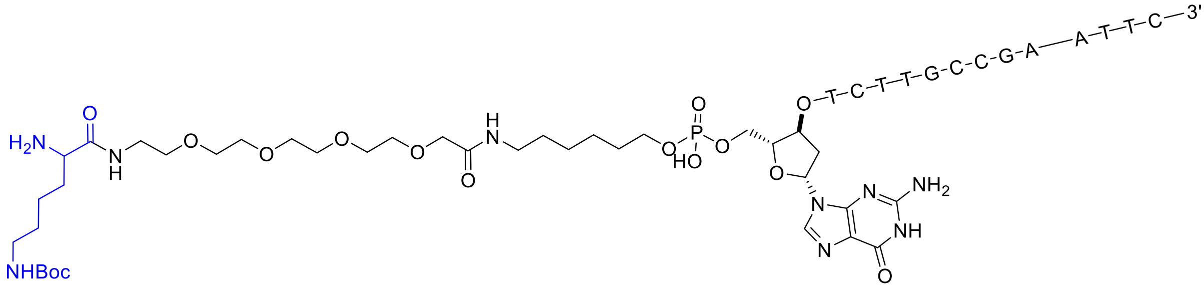

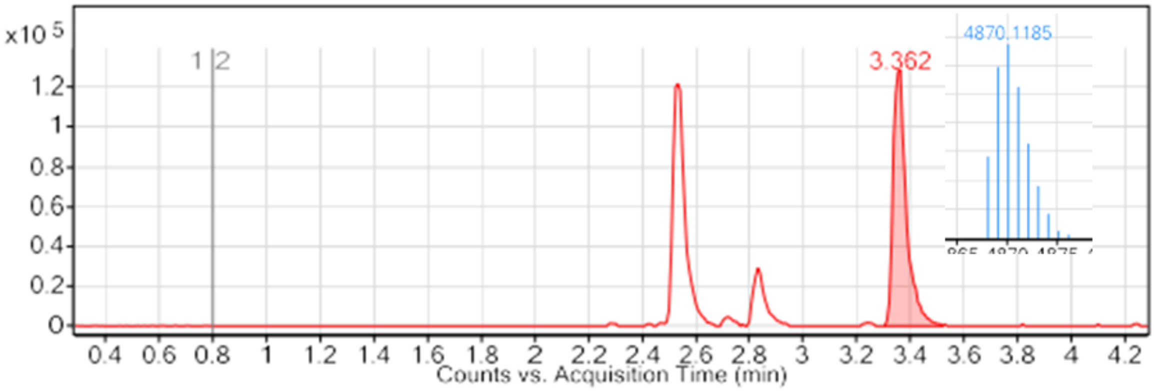

Calculated mass: 4868.0775
Observed mass: 4868.1115

Bn-Deprotection

*Figure S34: Chromatogram and deconvoluted mass spectrum of **34***

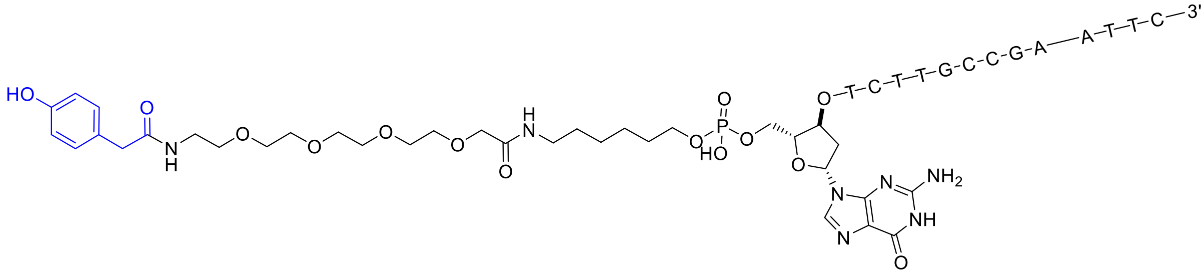

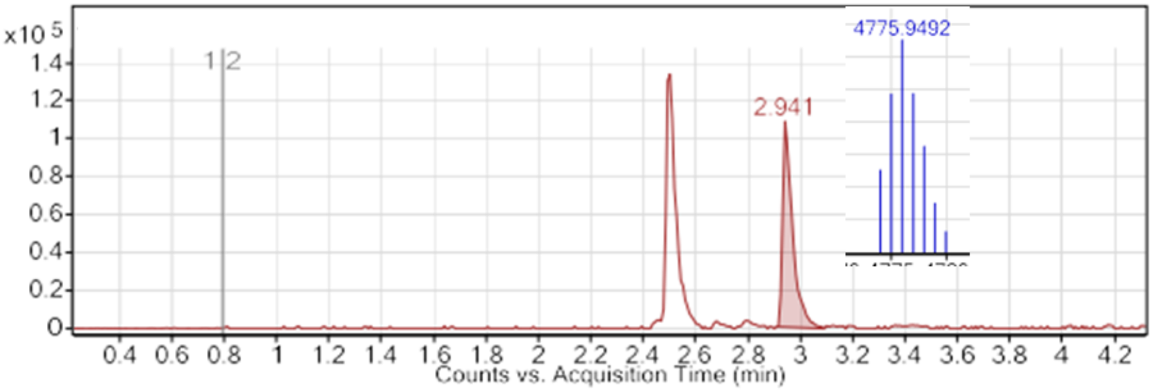

Calculated mass: 4773.9669
Observed mass:4773.9441

*Figure S35: Chromatogram and deconvoluted mass spectrum of **35***

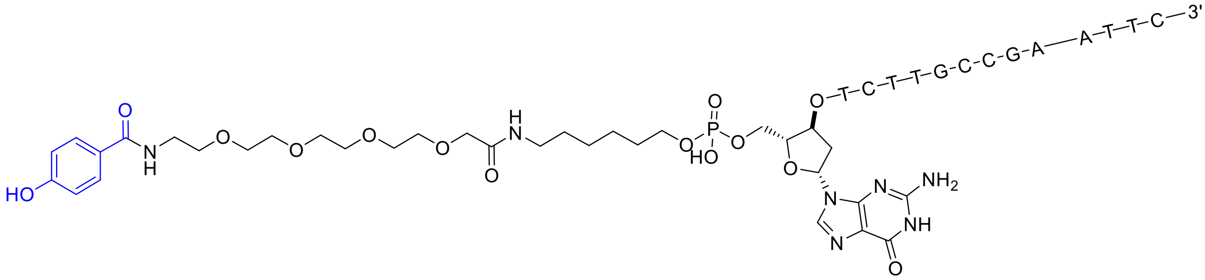

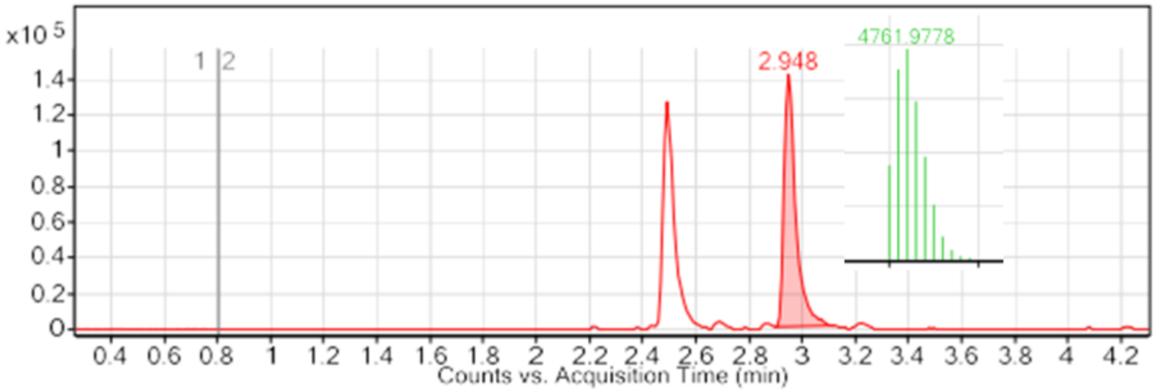

Calculated mass: 4759.9513
Observed mass: 4759.9711

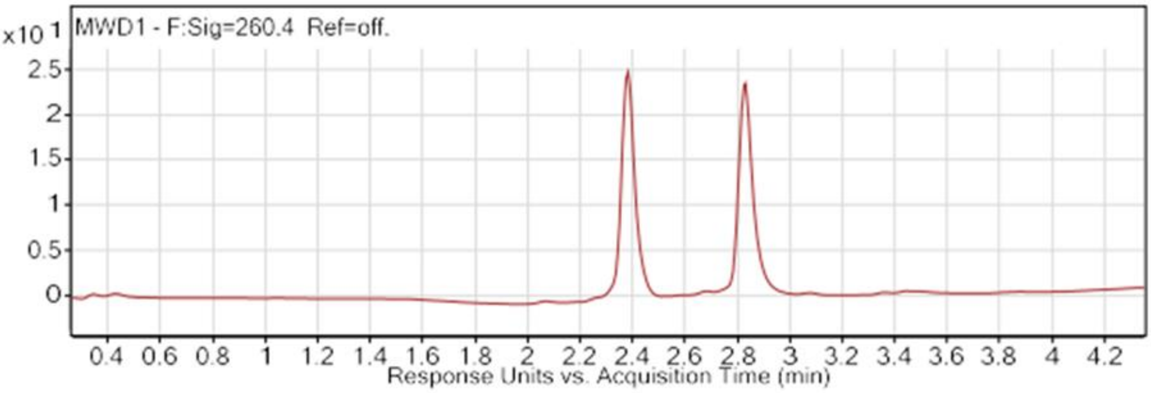

Figure S36: Chromatogram and deconvoluted mass spectrum of ***36***

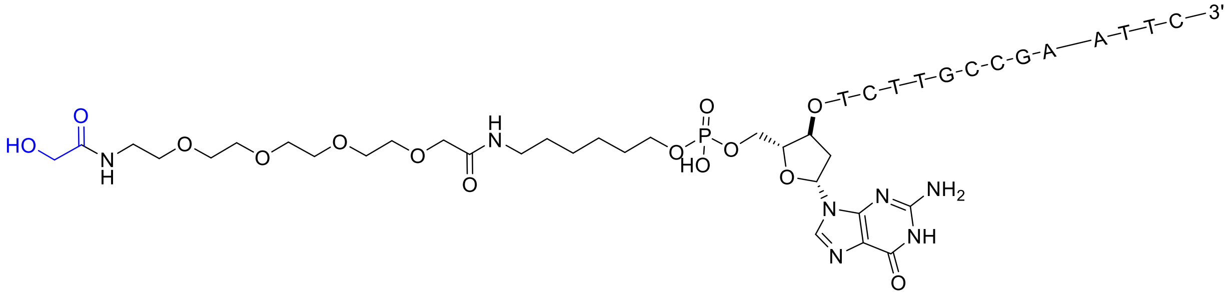

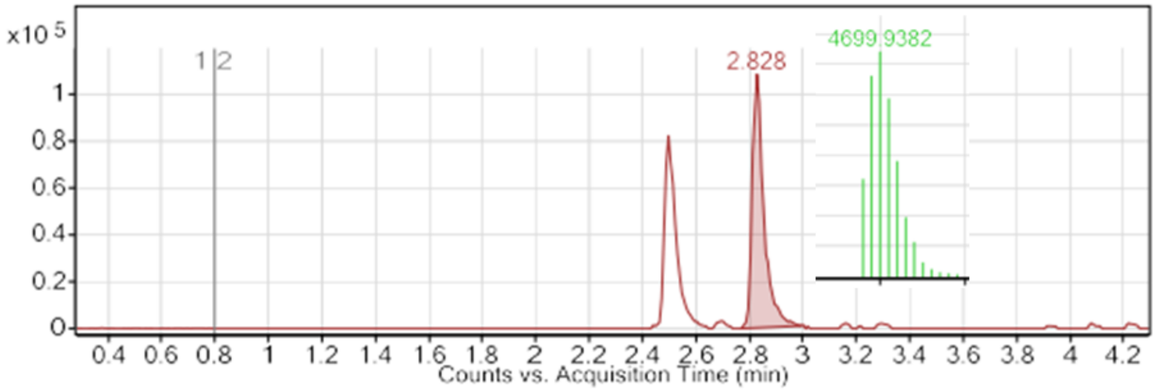

Calculated mass: 4697.9356
Observed mass: 4697.9341

Nitro Reductions

*Figure S37: Chromatogram and deconvoluted mass spectrum of **37***

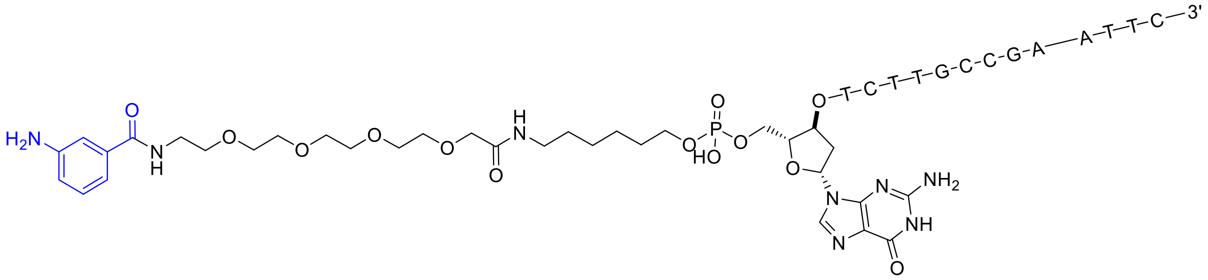

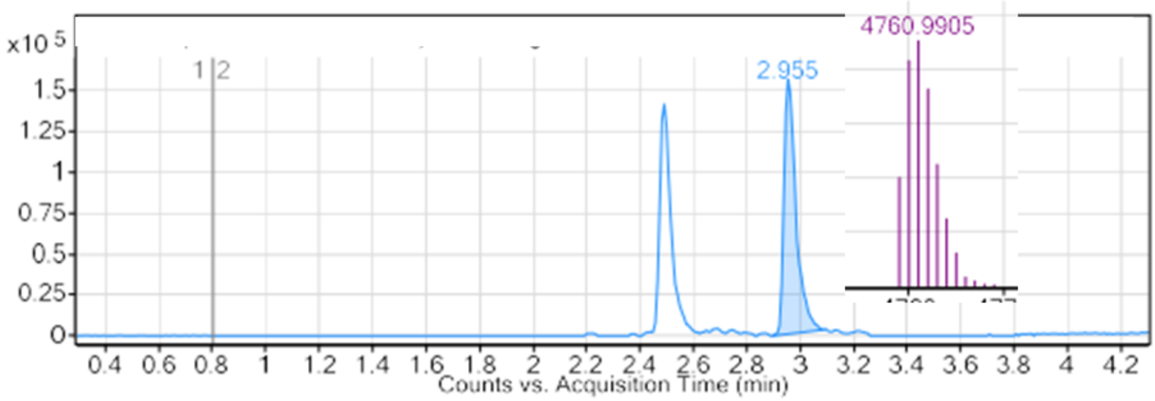

Calculated mass: 4758.9672

Observed mass: 4758.9823

*Figure S38: Chromatogram and deconvoluted mass spectrum of **38***

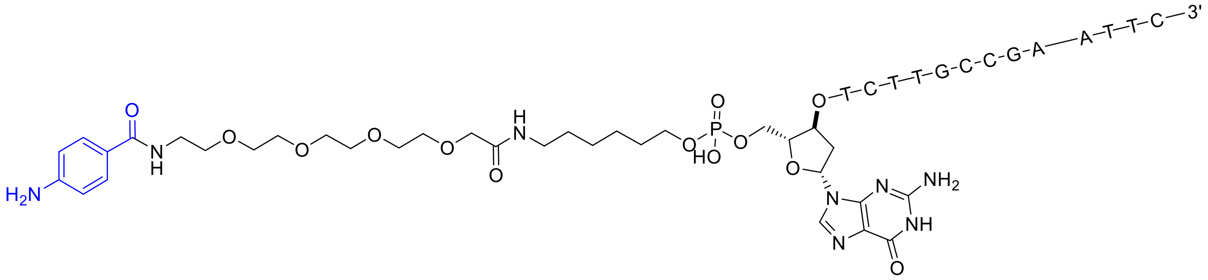

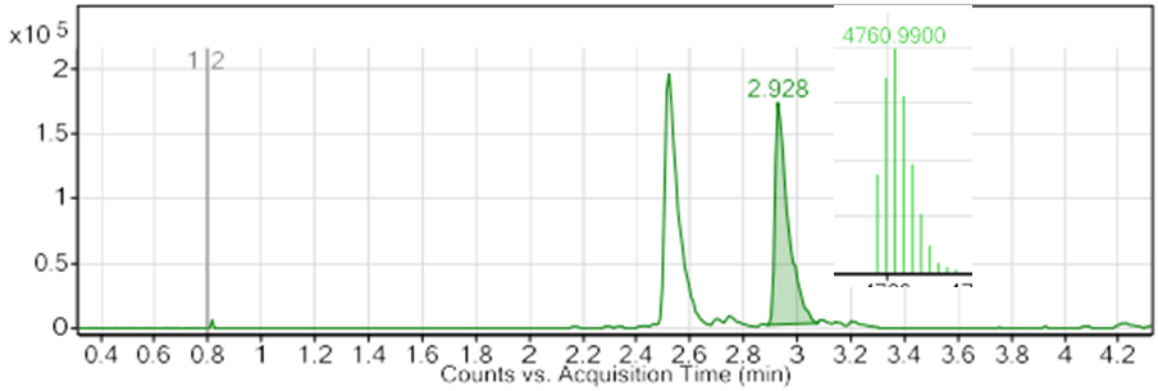

Calculated mass: 4758.9672

Observed mass: 4758.9811

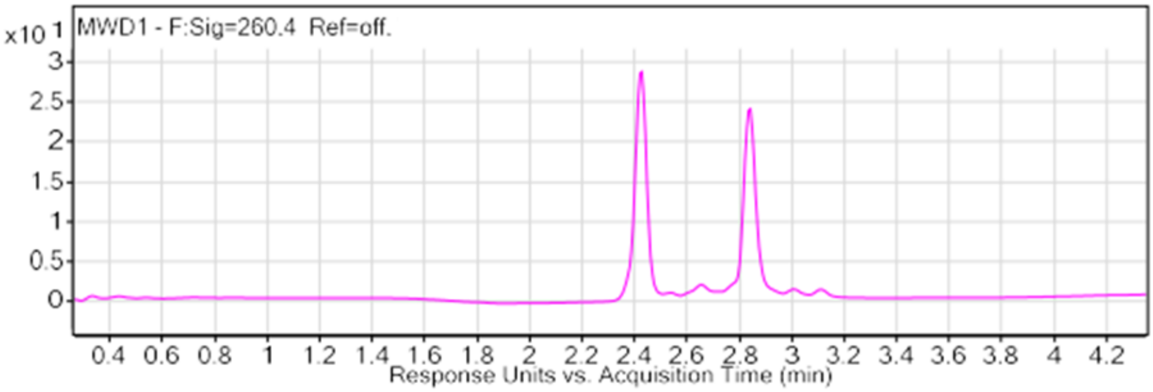

Figure S39: Chromatogram and deconvoluted mass spectrum of ***39***

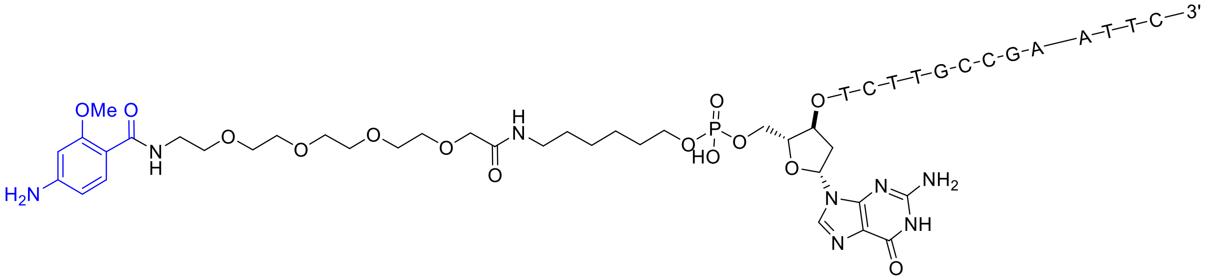

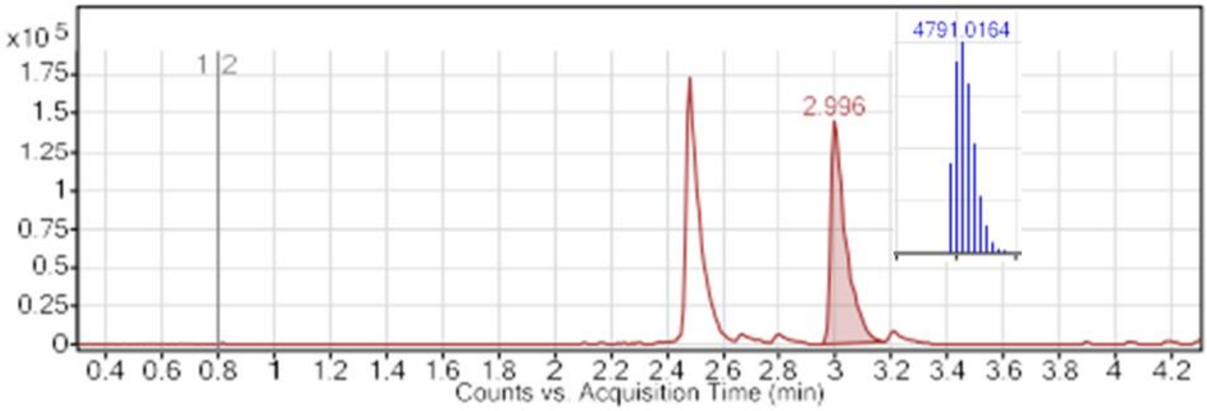

Calculated mass: 4788.9778

Observed mass: 4789.0105

Figure S40: Chromatogram and deconvoluted mass spectrum of ***40***

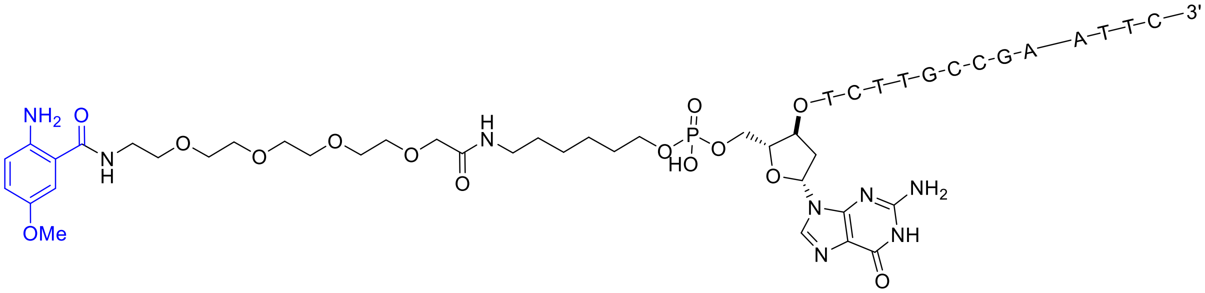

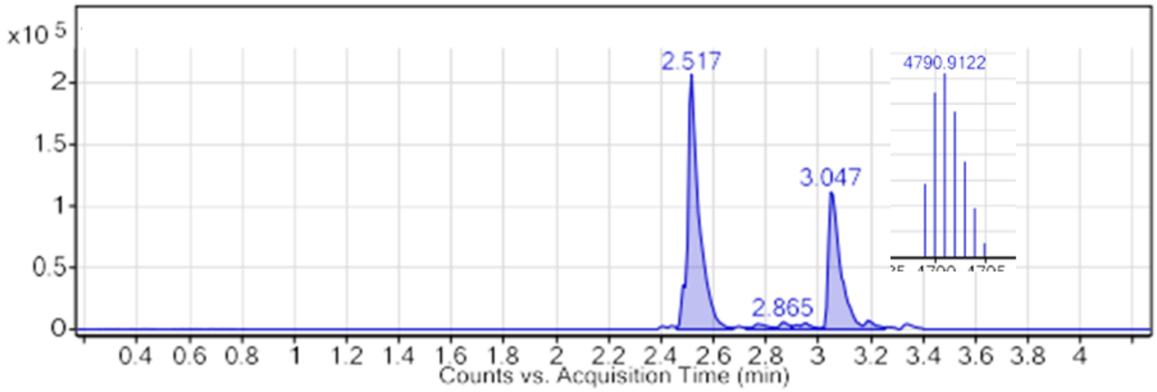

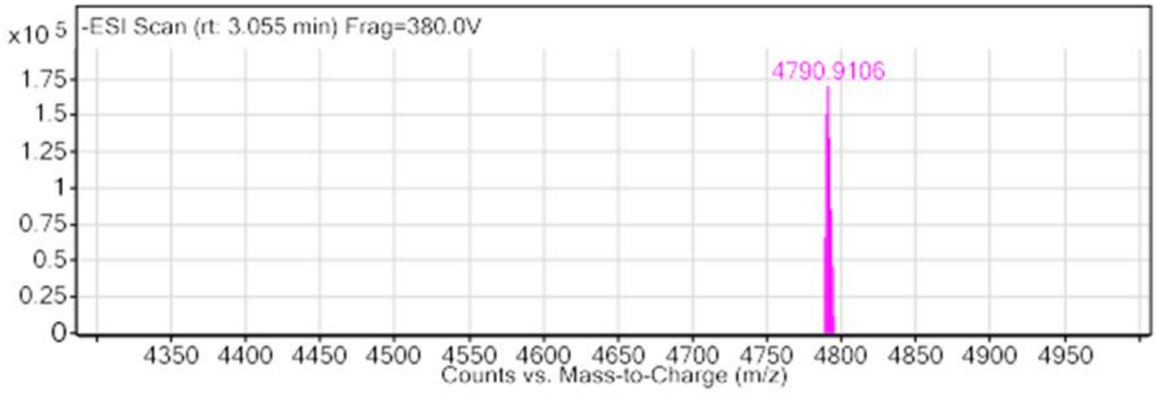

Calculated mass: 4788.9778
Observed mass: 4788.9063

*Figure S41: Chromatogram and deconvoluted mass spectrum of **41***

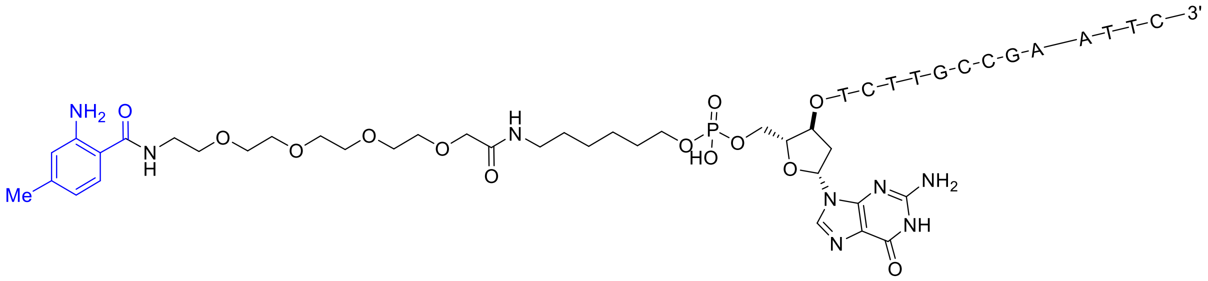

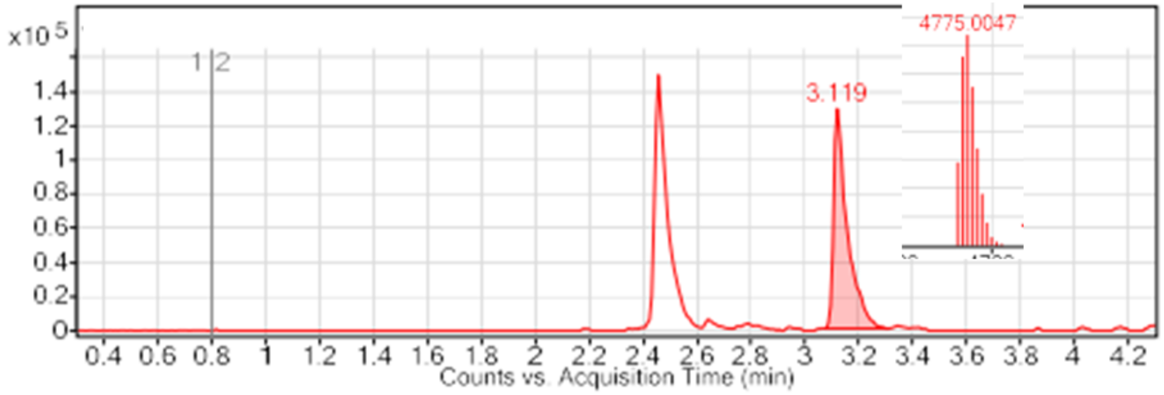

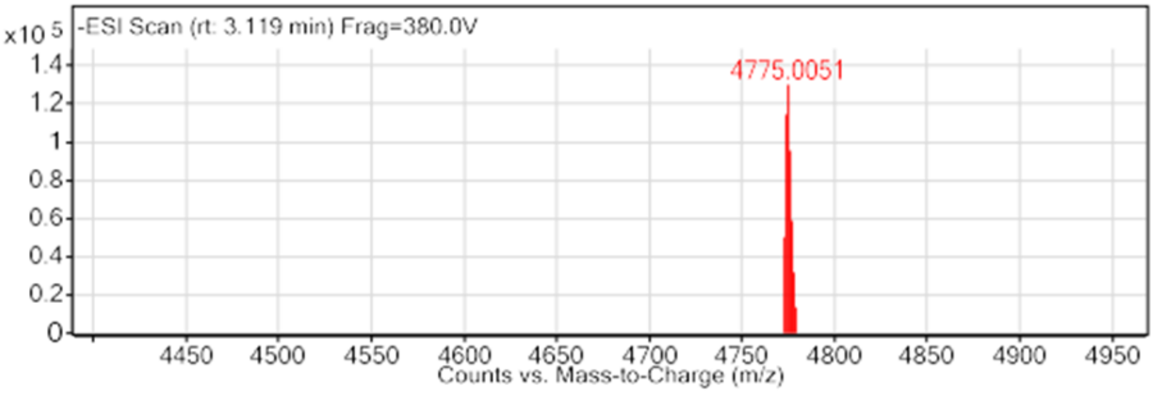

Calculated mass: 4772.9829
Observed mass: 4772.9984

*Figure S42: Chromatogram and deconvoluted mass spectrum of **42***

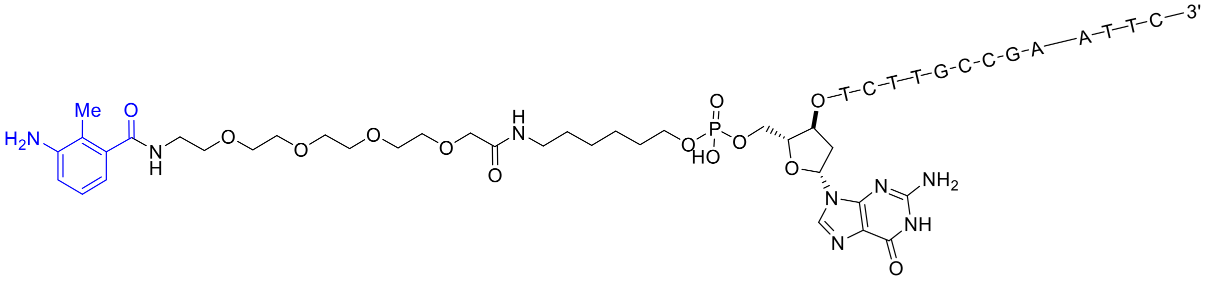

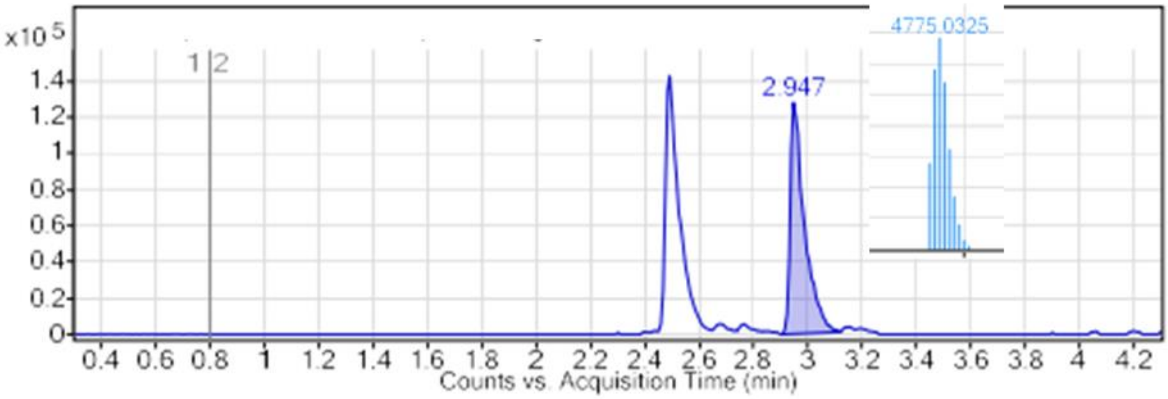

Calculated mass: 4772.9829
Observed mass: 4773.0271

Dehalogenations

*Figure S43: Chromatogram and deconvoluted mass spectrum of **43** (from **18**)*

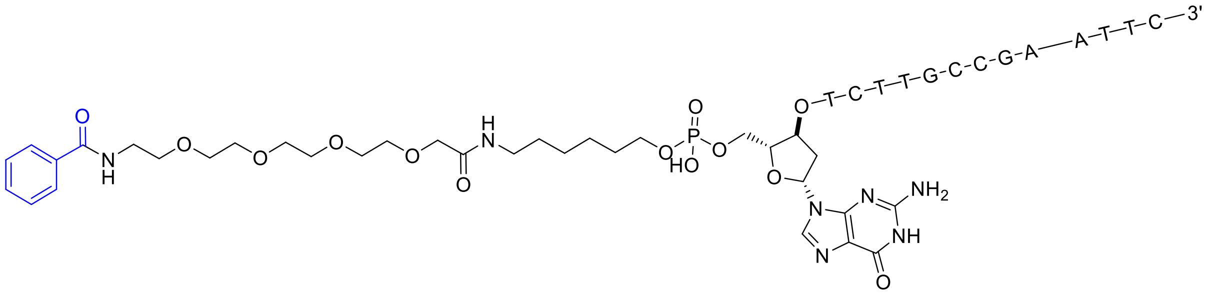

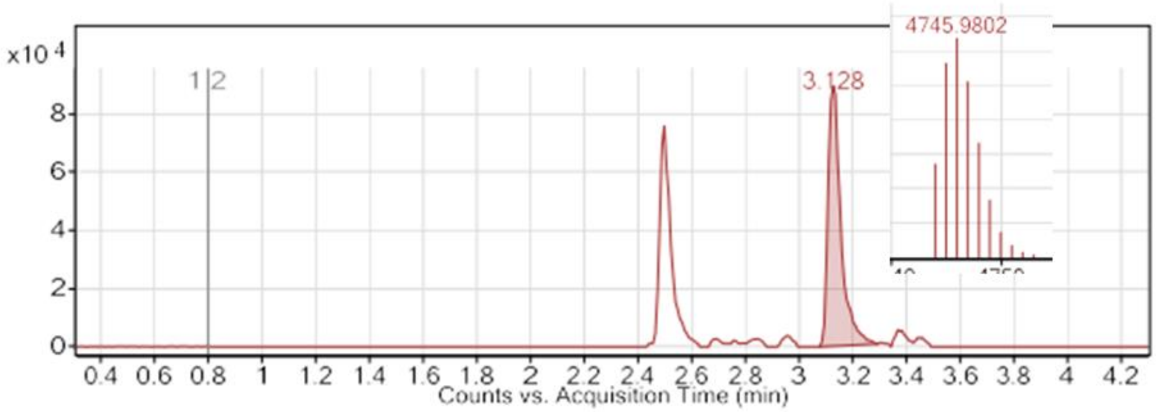

Calculated mass: 4743.9563
Observed mass: 4743.9776

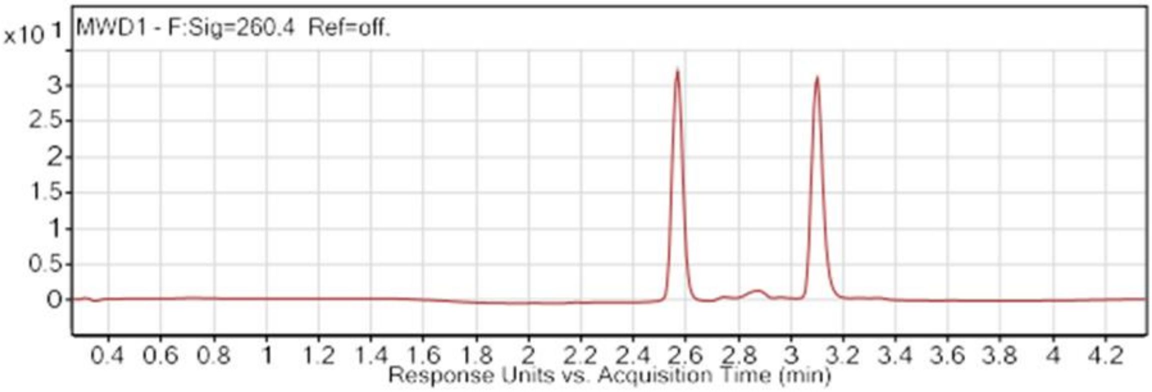

*Figure S44: Chromatogram and deconvoluted mass spectrum of **43** (from **19**)*

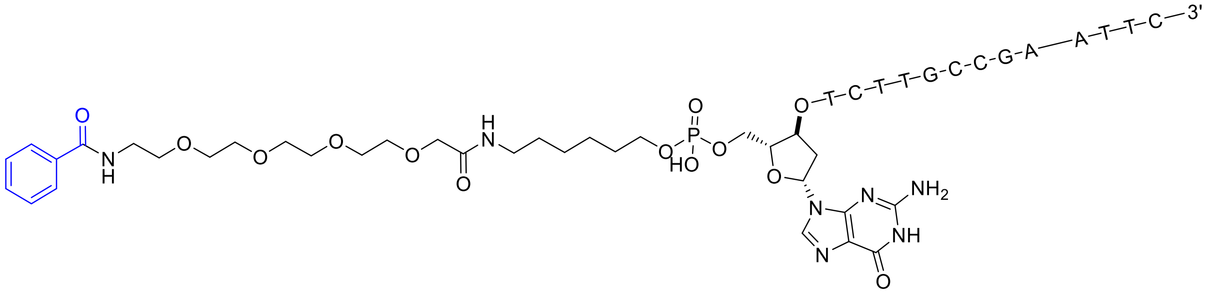

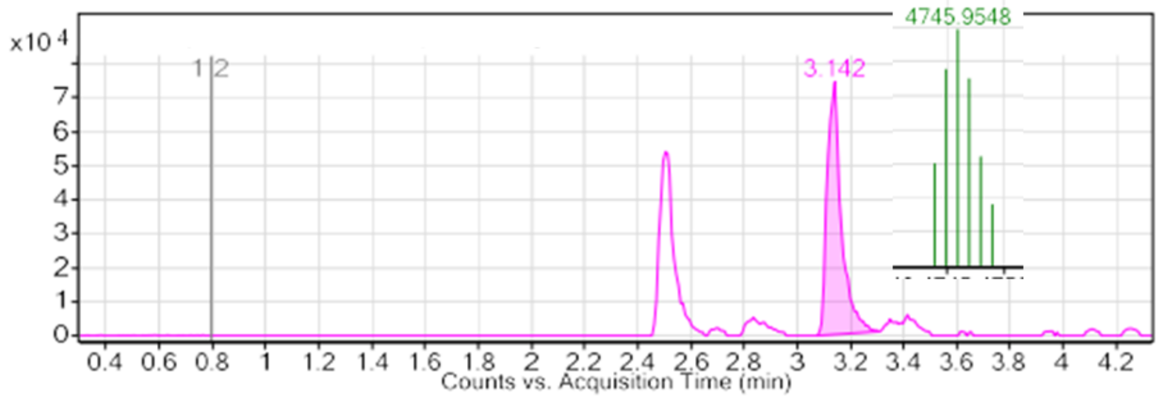

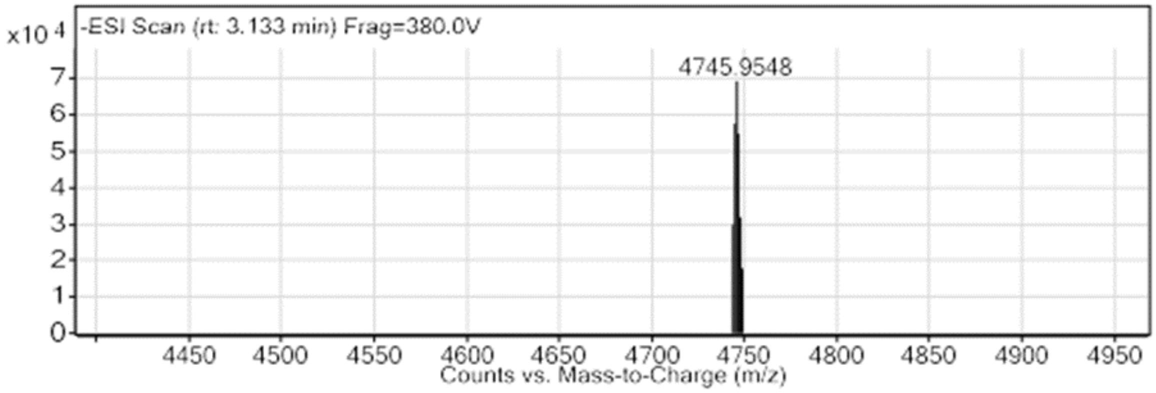

Calculated mass: 4743.9563
Observed masses: 4743.9467

*Figure S45: Chromatogram and deconvoluted mass spectrum of **44***

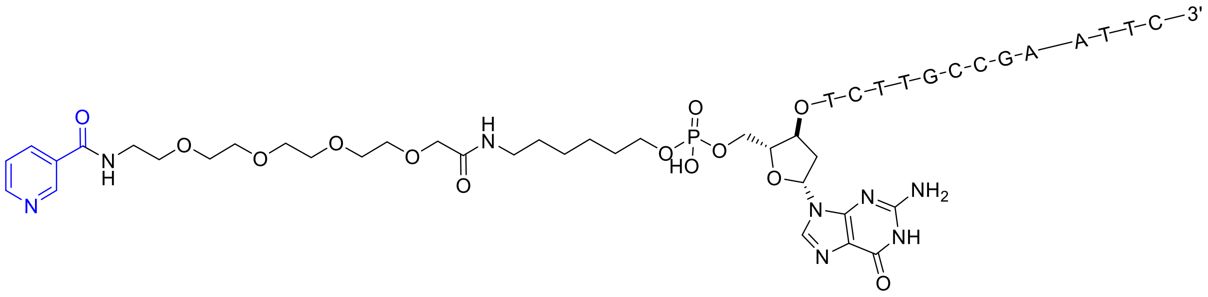

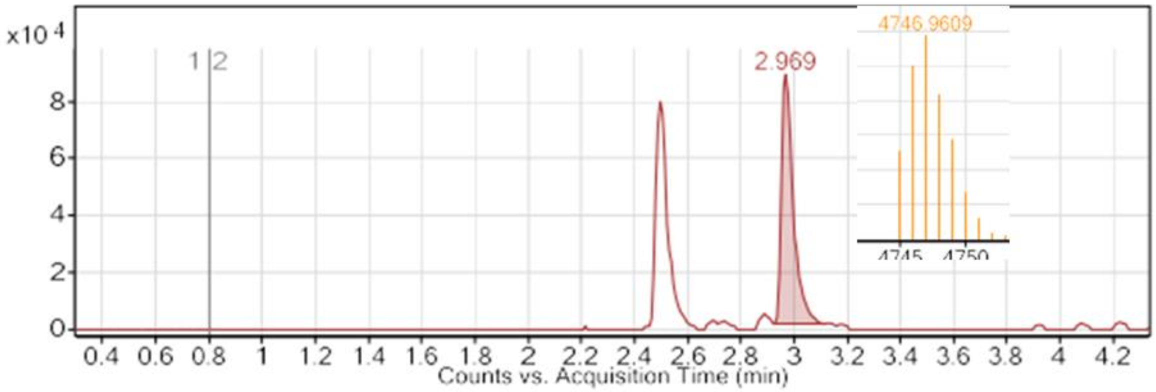

Calculated mass: 4744.9516

Observed mass: 4778.9548

Multiple Bond Reductions

*Figure S46: Chromatogram and deconvoluted mass spectrum of **45***

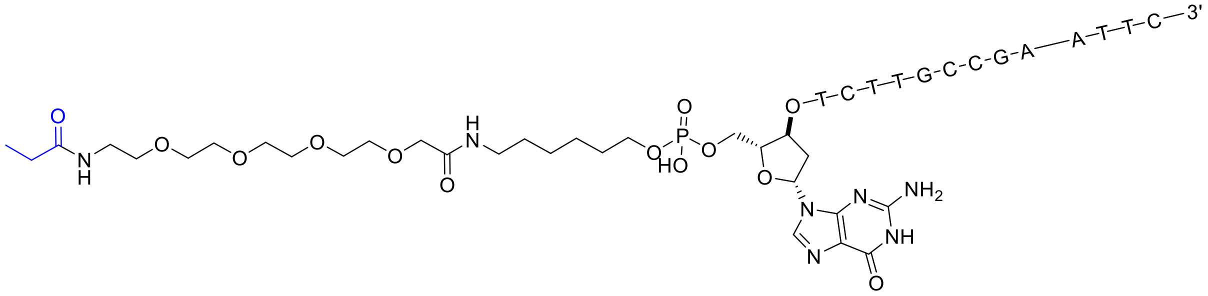

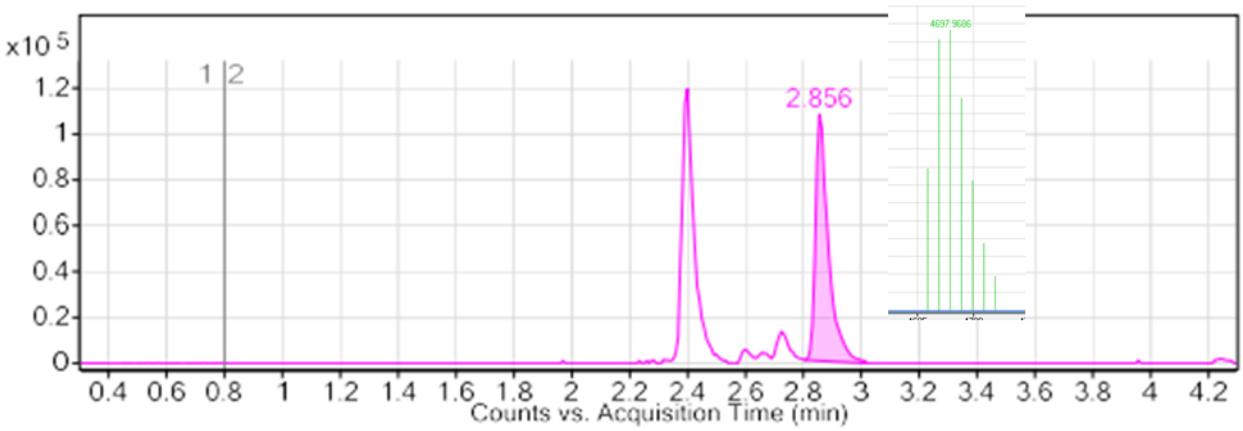

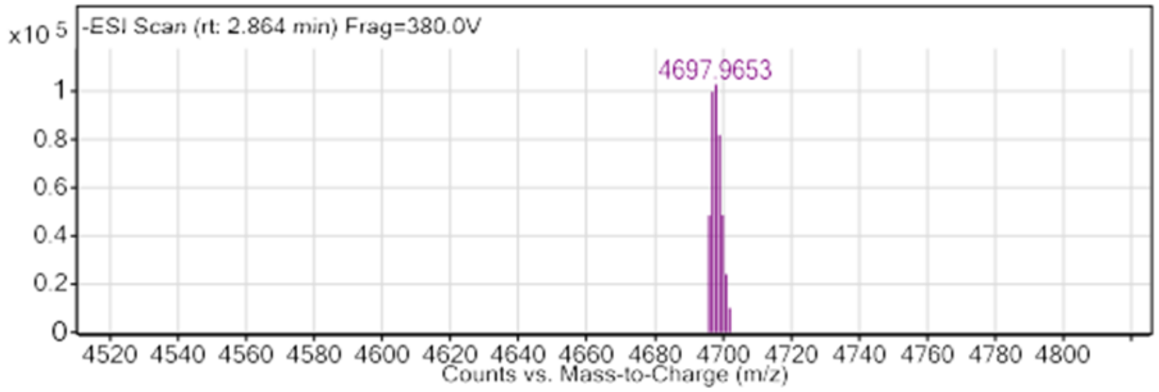

Calculated mass: 4695.9563
Observed mass: 4695.9599

*Figure S47: Chromatogram and deconvoluted mass spectrum of **46***

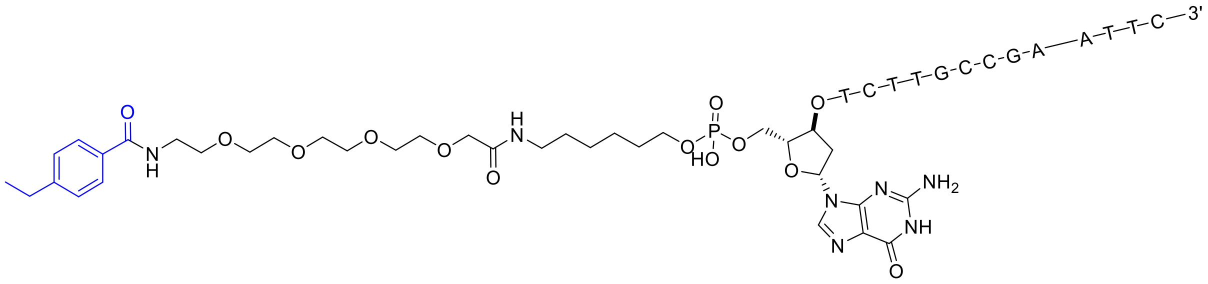

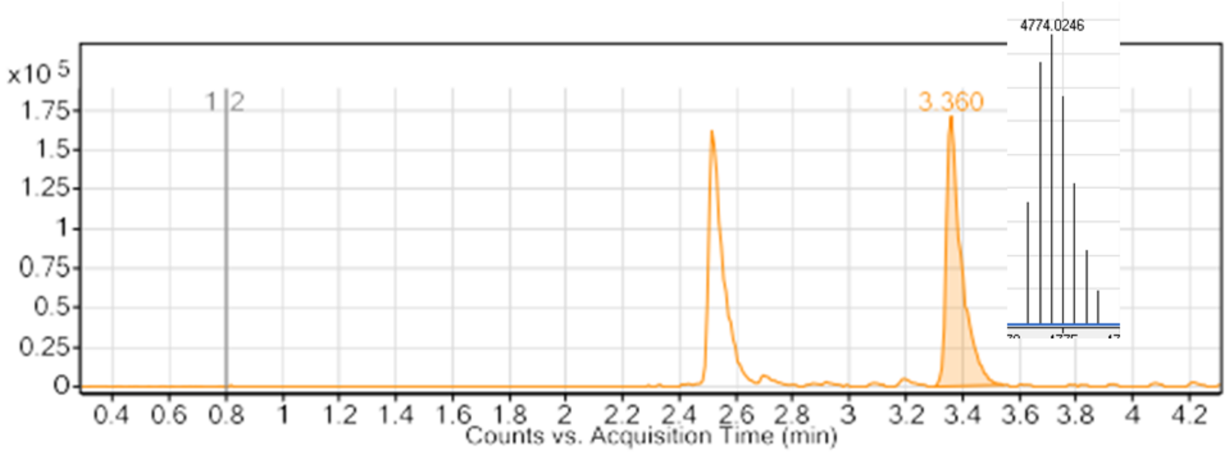

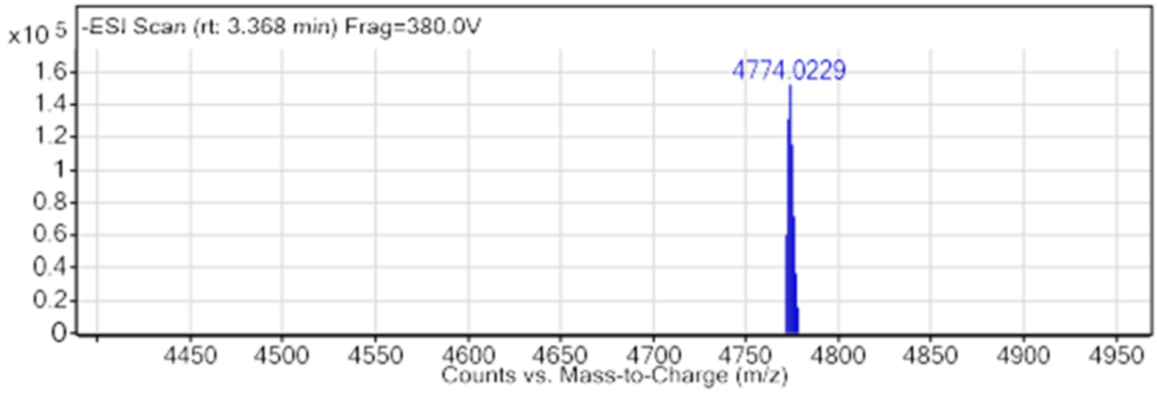

Calculated mass: 4771.9876
Observed mass: 4772.0140

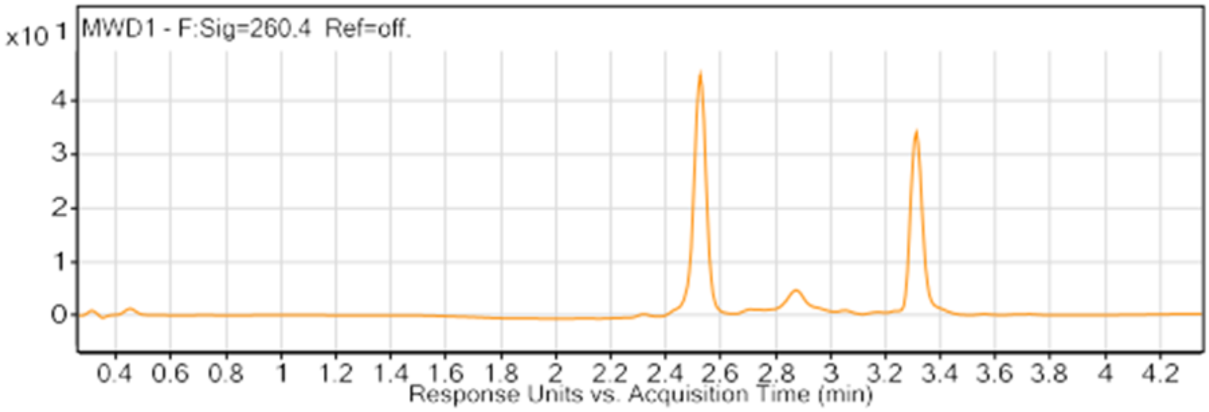

*Figure S48: Chromatogram and deconvoluted mass spectrum of **47** (from **23**)*

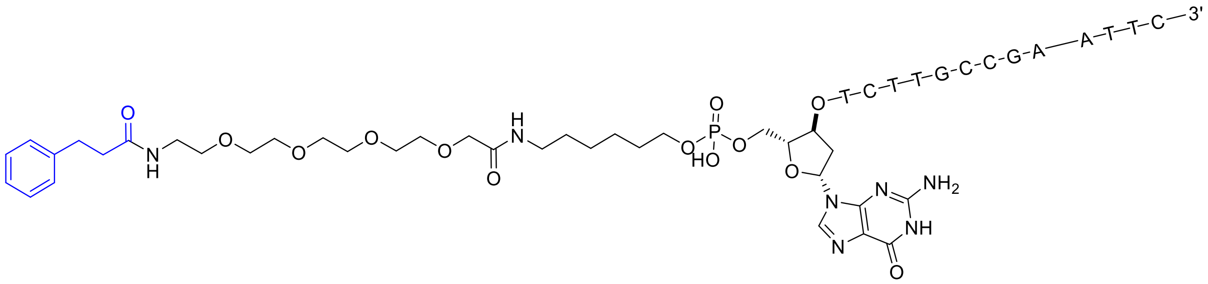

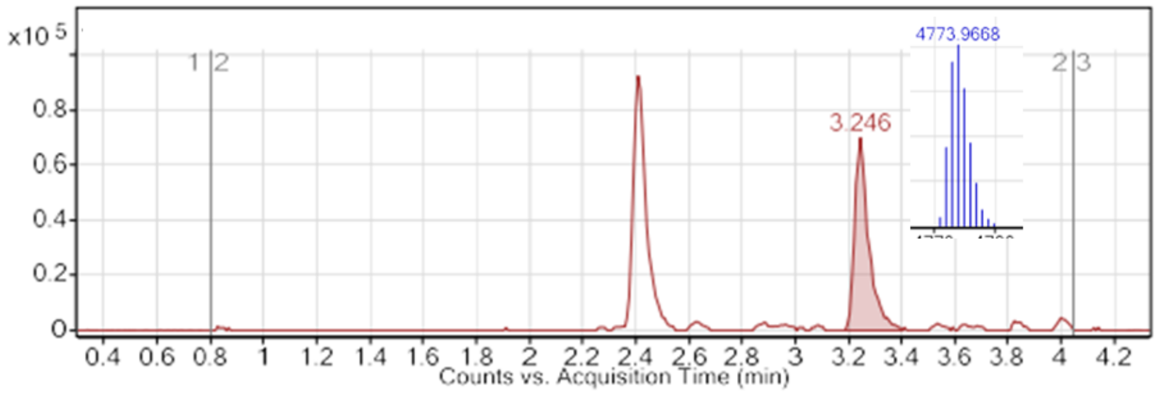

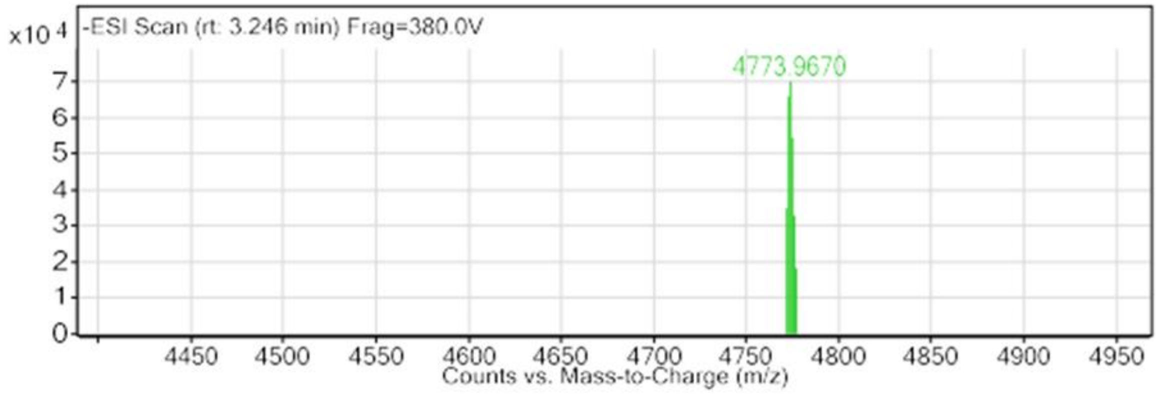

Calculated mass: 4771.9876
Observed mass: 4771.9581

*Figure S49: Chromatogram and deconvoluted mass spectrum of **47** (from **24**)*

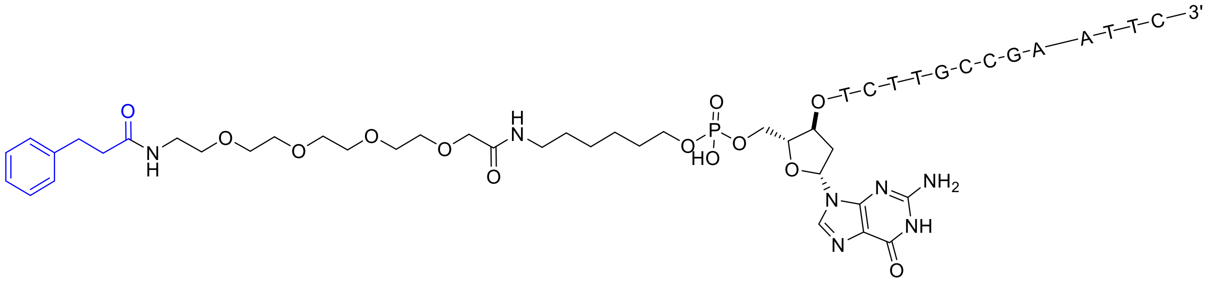

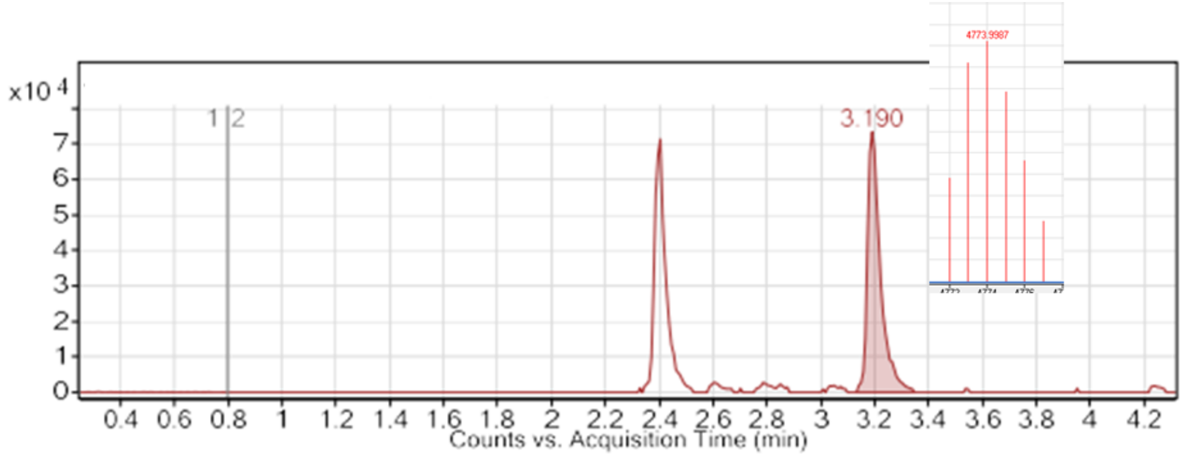

Calculated mass: 4771.9876
Observed mass: 4771.9925

Reduction of Other Functional Groups

*Figure S50: Chromatogram and deconvoluted mass spectrum of **48***

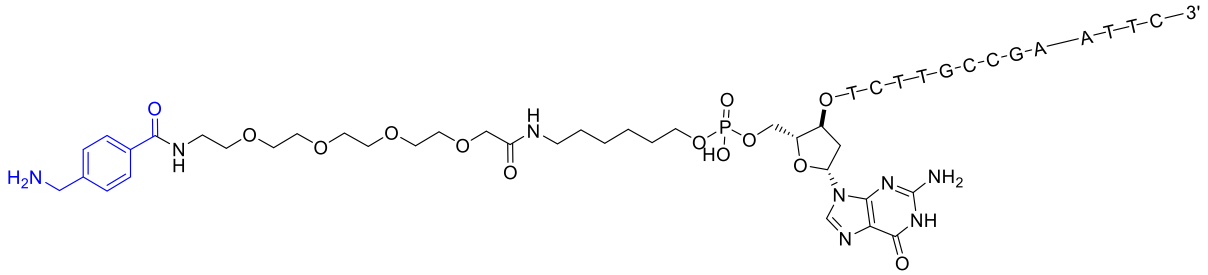

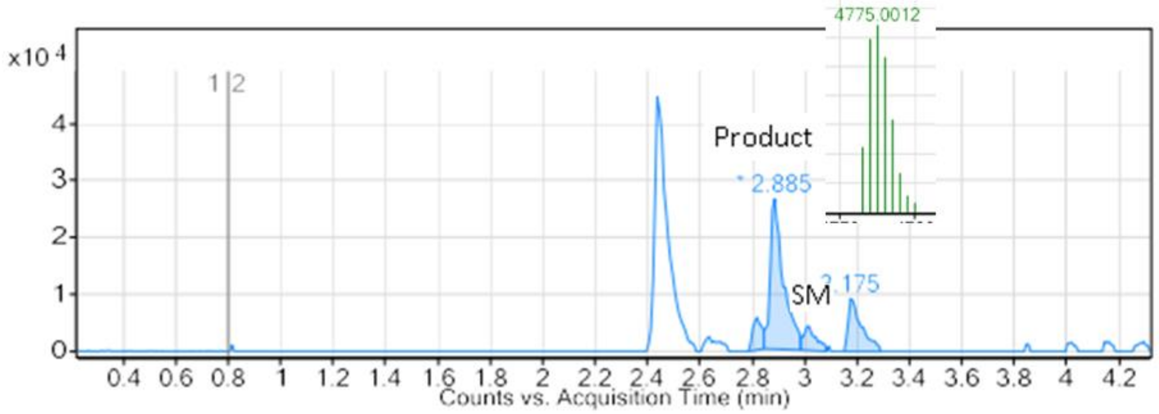

Calculated mass: 4772.9829
Observed mass: 4772.9981 (61%)

*Figure S51: Chromatogram and deconvoluted mass spectrum of **49***

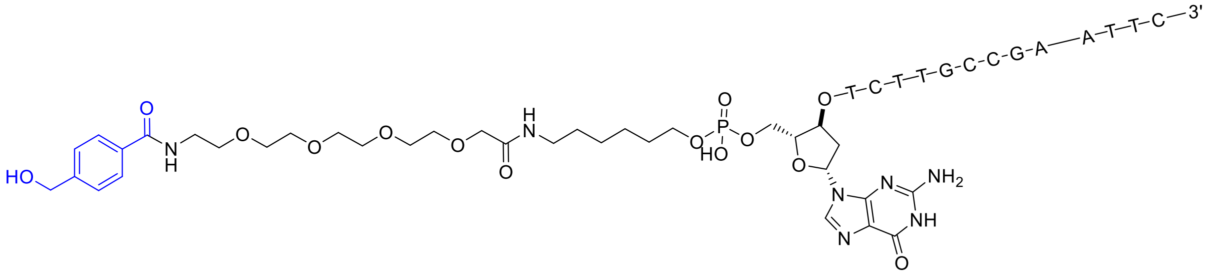

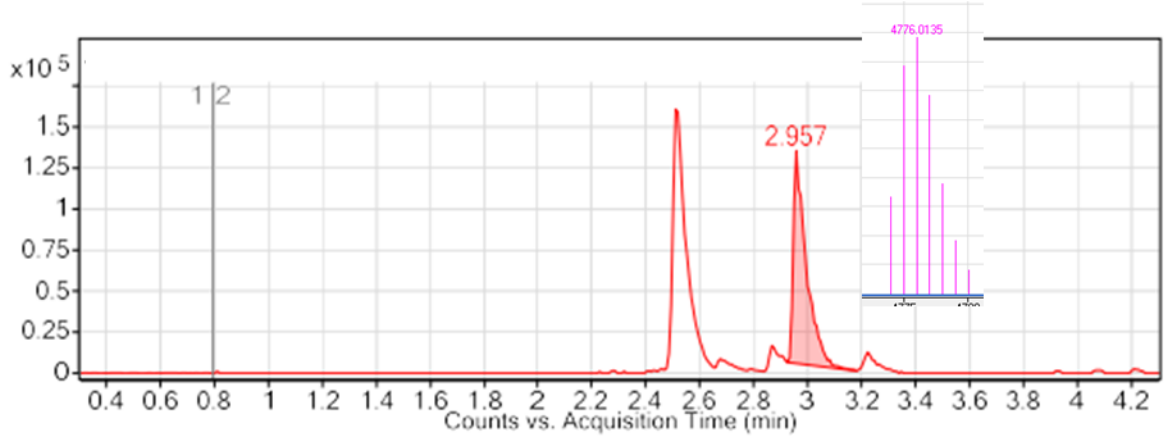

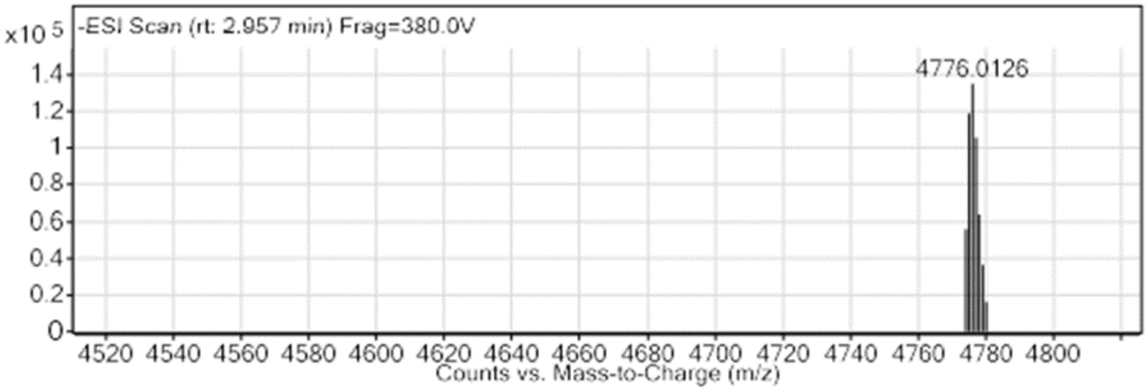

Calculated mass: 4773.9669
Observed mass: 4774.0055

Experimental Procedure for Transfer Hydrogenation without TPGS-750-M

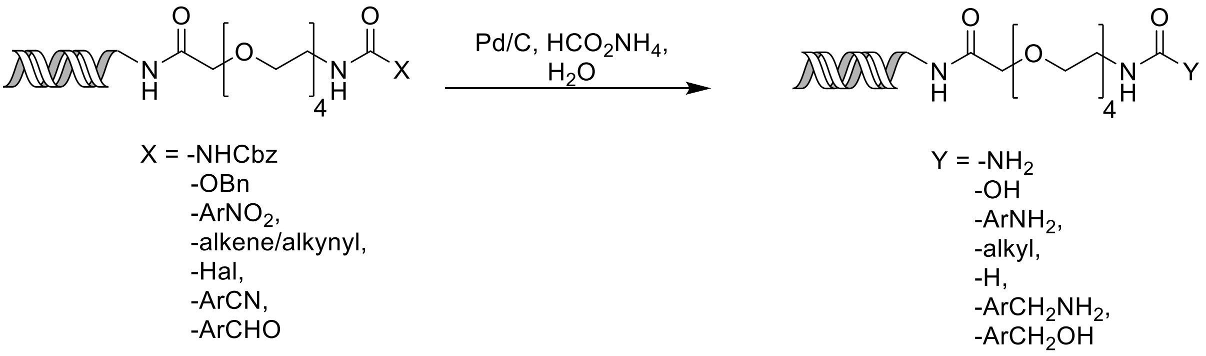

To a 50 µL glass insert for a Para-dox™ 96-well micro photoredox plate was added 10% wt Pd/C (5 µL, 400 mM in H_2O), water (18 µL) and DNA (4 µL, 0.25 mM in water). Samples were vortexed for 30 seconds each, then ammonium formate (3 µL, 5.3 M in water) was added, and the samples were vortexed for a further 10 seconds. Reactions were then shaken at 1200 rpm, at room temperature in a PMS-1000i Microplate shaker for up to 2 hours. The samples were diluted to 200 µL with water, filtered through a hydrophilic PTFE filter and analysed via mass spectrometry. Products were precipitated according to the general ethanol precipitation procedure.

Table S3: Results of Transfer Hydrogenation without TPGS-750-M

**No.**	Product	Conversion	DNA Recovery (pmol, %)	DNA Recovery from 2% TPGS (pmol, %)	DNA Recovery from 3% TPGS (pmol, %)
28	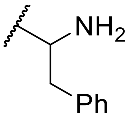	unquant	6 (0.6%)	745 (74.5%)	502 (50.2%)
36	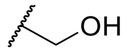	unquant	5 (0.5%)		

40	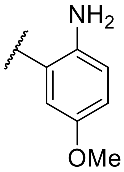	unquant	4 (0.4%)	728 (72.8%)	413 (41.3%)
46	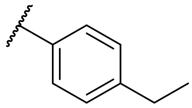	unquant	5 (0.5%)		

Experimental Procedure for Transfer Hydrogenation without shaking

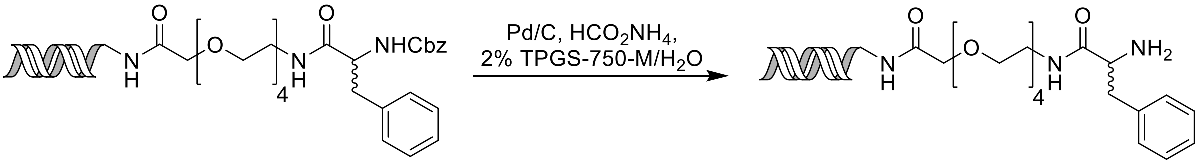

To a 50 µL glass insert for a Para-dox™ 96-well micro photoredox plate was added 10% wt Pd/C (5 µL, 400 mM in H_2O), 5% TPGS-750-M (12 µL) water (6 µL) and DNA (4 µL, 0.25 mM in water). The sample was vortexed for 30 seconds, then ammonium formate (3 µL, 5.3 M in water) was added, and the sample was vortexed for a further 10 seconds. The reaction was then stood at room temperature for 1 hour. The sample was diluted to 200 µL with water, filtered through a hydrophilic PTFE filter and analysed via mass spectrometry. Products were precipitated according to the general ethanol precipitation procedure.

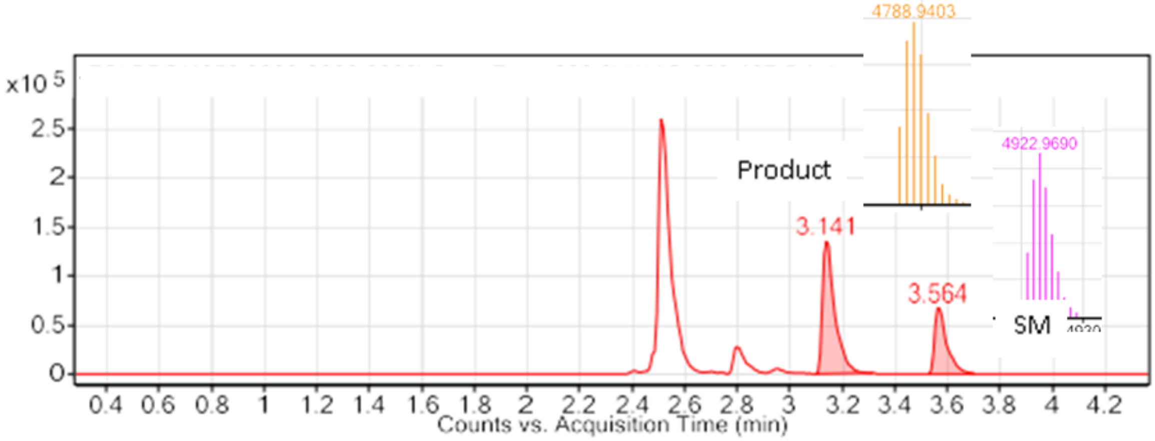

Comparison to Literature Reaction

(Torrado *et al, Bioconjugate Chem*. 2021, 32, 1, 88–93)

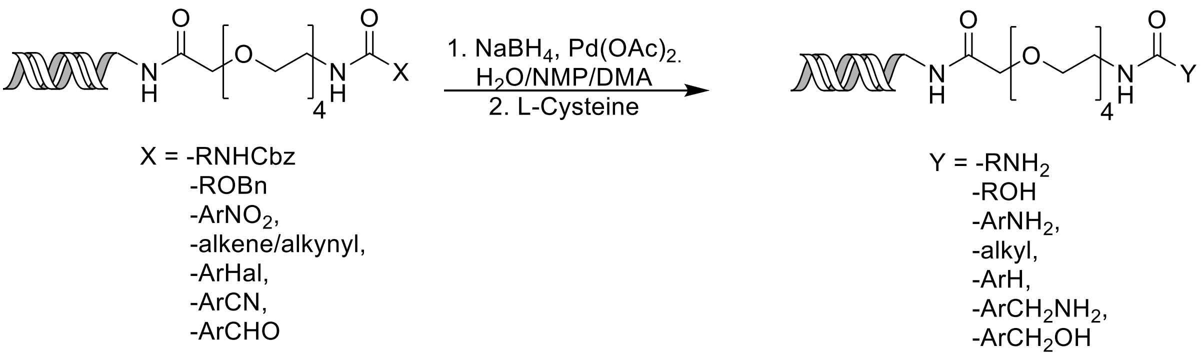

To a 1 mM solution of DNA-derivative in water (5 nmol, 5 µL) was added water (15 µL), sodium borohydride (2 µL, 400 mM in NMP) and palladium acetate (2 µL, 50 mM in DMA). The reaction was shaken at rt for 1h. L-Cysteine (20 µL, 300 mM in water) was then added and the suspension was shaken at rt for a further 4 hours. The solution was then diluted to 200 µL with water, filter through a hydrophilic PTFE filter and precipitated according to the general precipitation procedure before being analysed via mass spectroscopy.

Table S4: Results of application of literature method to selected substrates

No.	SM	Product	Conversion	Calculated mass	Observed mass
28	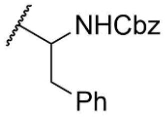	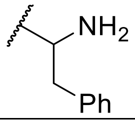	100	4786.9985	4786.9467
36	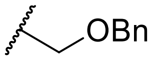	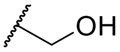	0	4697.9356	4787.9530 (SM)
48	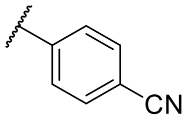	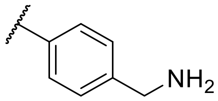	0	4772.9829	4768.8948 (SM)
49	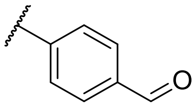	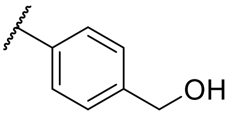	100	4773.9669	4773.9182

Application of Procedure in Construction of Dipeptide **52**

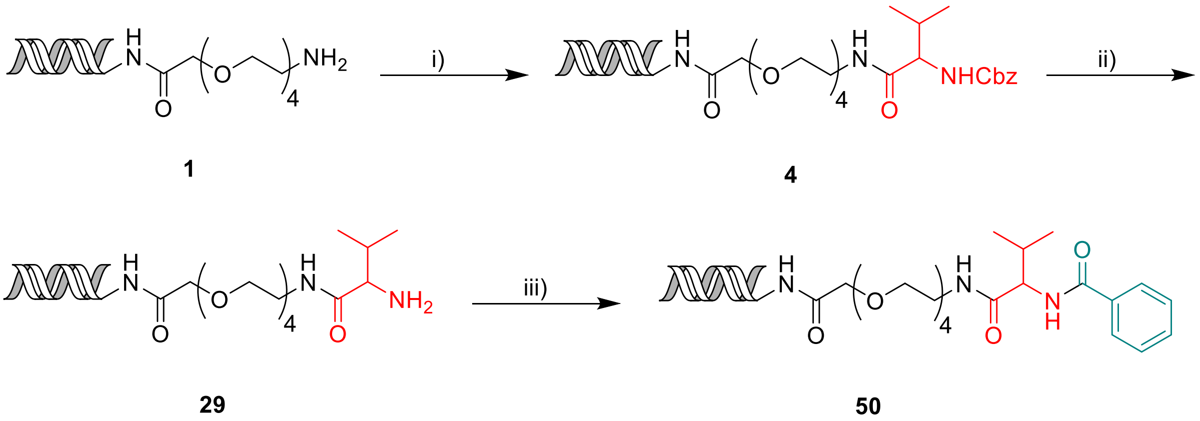

Conditions: i). Cbz-Val-OH (0.5 M), HATU (0.5 M), lutidine (2 M), 3.5% TPGS-750-M, 45°C, 16 h; ii) 10 wt% Pd/C (6.25 mM), HCO2NH4 (0.5 M), 2% TPGS-750-M, rt, 1200 rpm, 2 h; iii) Benzoic acid ((0.5 M), HATU (0.5 M), lutidine (2 M), 3.5% TPGS-750-M, 45°C, 16 h

*Experimental Procedure and Characterisation of **4***

HATU (5.7 mg, 15 µmol) and Cbz-Valine-OH (3.73 mg, 15 µmol) were added to a 50 µL glass insert for a Para-dox™ 96-well micro photoredox plate. 5% TPGS-750-M (21 µL) and HP **1** (9 µL, 1 mM in H_2O) were added to the vial, followed by lutidine (6.92 µL, 60 µmol). Samples were vortexed for 30 seconds each, and then heated in a Para-dox™ 96-well micro photoredox plate at 45 °C for 16 hours. Samples were then diluted to 200 µL with H_2O; DCM (2 x 400 µL) was added, and the samples were vortexed. The organic layer was discarded, the sample was filtered through a hydrophilic PTFE filter and analysed via mass spectrometry. Products were then precipitated according to the general ethanol precipitation procedure. 88% conversion, 46% yield. Calculated mass: 4873.0353. Observed mass: 4873.0032

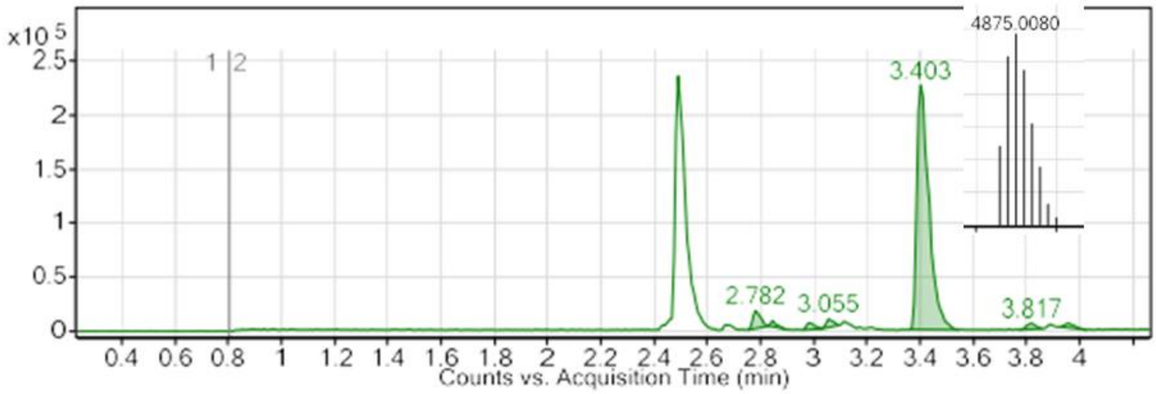

*Experimental Procedure and Characterisation of **29***

To a 50 µL glass insert for a Para-dox™ 96-well micro photoredox plate was added 10% wt Pd/C (5 µL, 400 mM in H_2O), 5% TPGS-750-M (12 µL) and **4** (10 µL, 0.41 mM in water). Samples were vortexed for 30 seconds each, then ammonium formate (3 µL, 5.3 M in water) was added, and the samples were vortexed for a further 10 seconds. Reactions were then shaken at 1200 rpm, at room temperature in a PMS-1000i Microplate shaker for 2 hours. The samples were diluted to 200 µL with water, filtered through a hydrophilic PTFE filter and analysed via mass spectrometry. Products were precipitated according to the general ethanol precipitation procedure. 100% conversion, 41% yield. Calculated mass: 4738.9985 Observed mass: 4738.9286

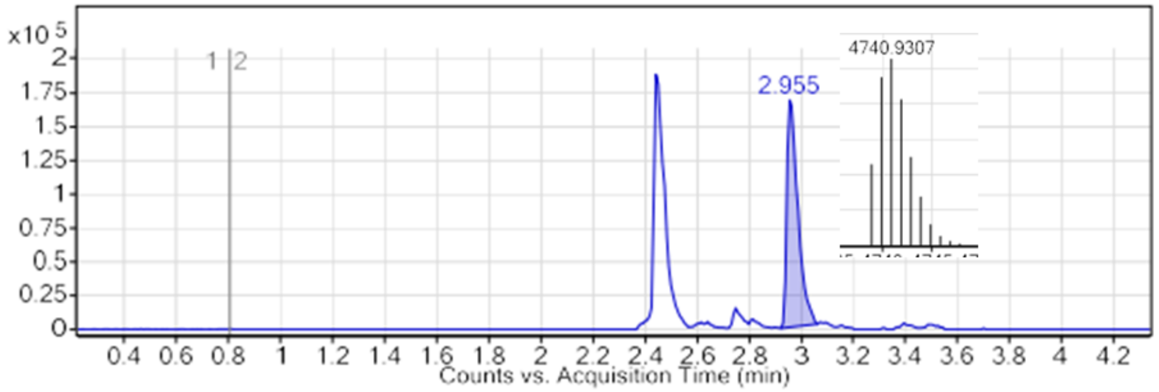

*Experimental Procedure and Characterisation of **50***

HATU (5.7 mg, 15 µmol) and benzoic acid (1.83 mg, 15 µmol) were added to a 50 µL glass insert for a Para-dox™ 96-well micro photoredox plate. 5% TPGS-750-M (21 µL) and **29** (9 µL, 0.19 mM in H_2O) were added to the vial, followed by lutidine (6.92 µL, 60 µmol). Samples were vortexed for 30 seconds each, and then heated in a Para-dox™ 96-well micro photoredox plate at 45 °C for 16 hours. Samples were then diluted to 200 µL with H_2O; DCM (2 x 400 µL) was added, and the samples were vortexed. The organic layer was discarded, the sample was filtered through a hydrophilic PTFE filter and analysed via mass spectrometry. Products were then precipitated according to the general ethanol precipitation procedure. 93% conversion, 61% yield. Calculated mass: 4843.0248. Observed mass: 4842.9795

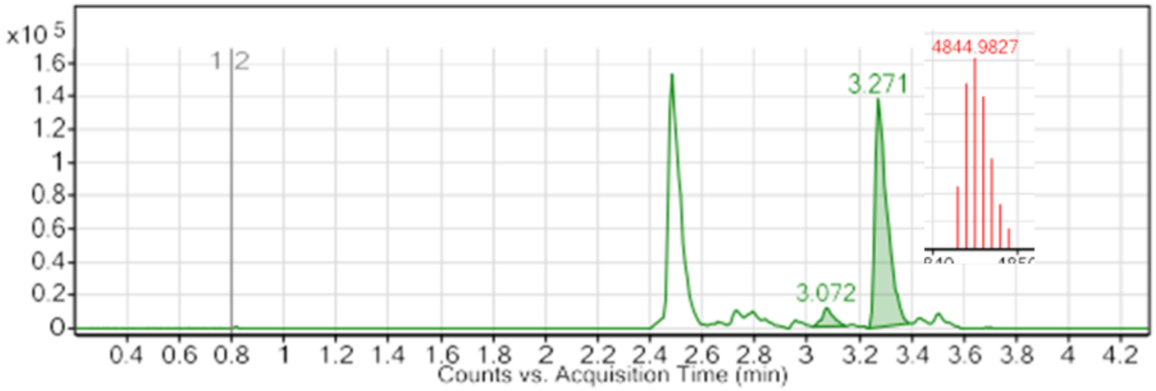

Construction of 1x1 Library

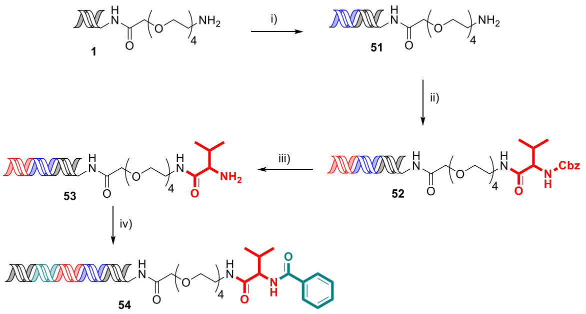

Conditions: i) Ligation (primer and library codon), 100% yield; ii) a. Ligation (first monomer codon), b. Cbz-Val-OH (0.5 M), HATU (0.5 M), lutidine (2 M), 3.5% TPGS-750-M, 45 °C, 16 h, iii) 10 wt% Pd/C (6.25 mM), HCO_2NH_4 (0.5 M), 2% TPGS-750-M, rt, 1200 rpm, 2 h, 59% yield over 3 steps; iv) a) Ligation (closing primer and second monomer codon), b) benzoic acid ((0.5 M), HATU (0.5 M), lutidine (2 M),

3.5% TPGS-750-M, 45 °C, 16 h, 58% yield over 2 steps. Yields determined by Nanodrop™ spectrophotometry.

The following code abbreviations for each DNA section have been used:

Code	Function	Sequence (5'-3')
A	Adapter – 5' amino-linked head piece	GTCTTGCCGAATTC
A'	Complimentary adapter	GAATTCGGCAAGAC
P	Primer	AGGTCGGTGTGAACGGATTTG
P'	Complementary primer	CAAATCCGTTCACACCGACCT
OH1	Ligation overhang 1	GTAT
OH1'	Complementary OH1	ATAC
BB1	Building block 1	GCACACGC
BB1'	Complementary BB1	GCGTGTGC
OH2	Ligation overhang 2	CCTA
OH2'	Complementary OH2	TAGG
BB2	Building block 2	GCATGTAC
BB2'	Complementary BB2	GTACATGC
OH3	Ligation overhang 3	TACG
OH3'	Complementary OH3	CGTA
BB3	Building block 3	AATATTGC
BB3'	Complementary BB3	GCAATATT
P2	Complementary to P2'	TGACCTCAACTACATGGTCTACA
P2'	Primer (reverse)	TGTAGACCATGTAGTTGAGGTCA

Ligation Strategy:

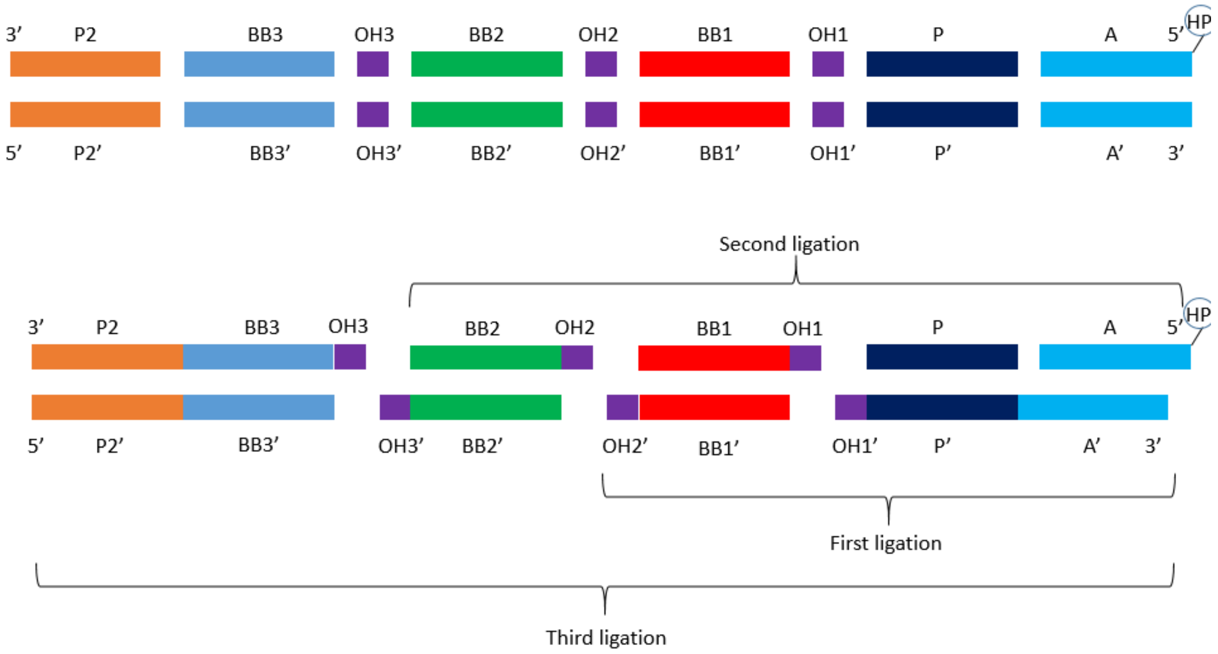

Cycle 1

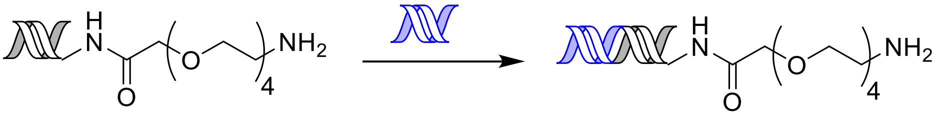

Sequences involved in the first ligation included adapter (A), primer (P), overhangs (OH) and building block 1 (BB1). Prior to ligations, the 5' terminus of any DNA strands that required ligating was phosphorylated; for cycle 1 this refers to sequences P (AGGTCGGTGTGAACGGATTTG), OH1BB1 (GTATGCACACGC) and A'P'OH1' (ATAC CAAATCCGTTCACACCGACCT GAATTCGGCAAGAC). To the DNA strands (50 µM, 1000 pmol in overall reaction media of 20 µL) was added PNK reaction buffer (2 µL, 500 mM Tris-HCl [pH 7.6 at 25 °C], 100 mM $MgCl_2$, 50 mM DTT, 1 mM spermidine), ATP (2 µL, 10 Mm, Thermo Scientific), T4 Polynucleotide Kinase (1 µL, 10U/µL. Thermo scientific) and nuclease free water (up to 20 µL). The reaction was conducted at 37 °C for 1 hour, followed by heating to 75 °C for 10 minutes. DNA was used in the subsequent ligation without purification or preparation.

Ligations were performed in 200 µL PCR tubes, using 20 µL of each phosphorylation reaction mixture, alongside 10 µL of 0.1 mM solutions of non-phosphorylated A (GTCTTGCCGAATTC) and OH2'BB1' (TAGG GCGTGTGC). To the DNA strands was added 10X T4 DNA ligase buffer (9 µL, 400 mM Tris-HCl, 100 mM $MgCl_2$, 100 mM DTT, 5 mM ATP), water (up to 90 µL) and T4 DNA Ligase (3 µL, 30 Weiss U/µL). Ligations were conducted at 25 °C for 16 hours, followed by heating to 75 °C for 10 minutes. Product **51** was then visualised by gel electrophoresis, showing a band at ca. 50 base pairs in length (expected 47 and 51 base pairs), before being purified by EtOH precipitation. The resulting pellet was then reconstituted in water to form at 0.1 mM solution.

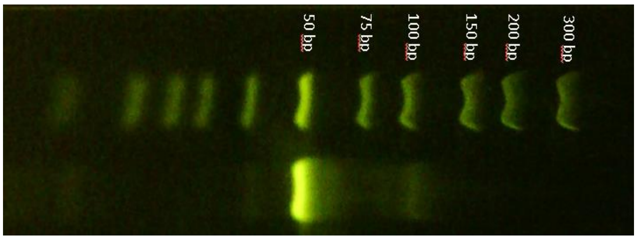

Cycle 2

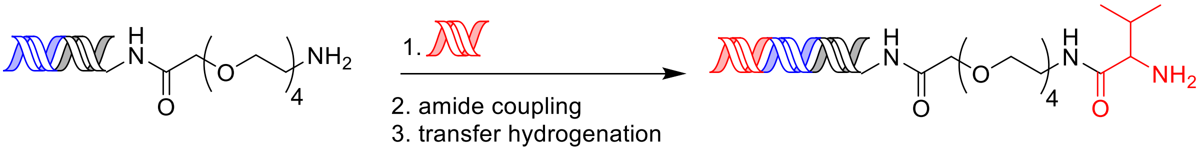

Sequences involved in the second ligation included **51**, overhangs (OH) and building block 1 (BB2). Prior to ligations, the 5' terminus of any DNA strands that required ligating was phosphorylated; for cycle 2 this refers to sequences **51** and OH2BB2 (CCTA GCATGTAC). To the DNA strands (50 µM, 1000 pmol in overall reaction media of 20 µL) was added PNK reaction buffer (2 µL, 500 mM Tris-HCl [pH 7.6 at 25 °C], 100 mM $MgCl_2$, 50 mM DTT, 1 mM spermidine), ATP (2 µL, 10 Mm, Thermo Scientific), T4 Polynucleotide Kinase (1 µL, 10U/µL. Thermo scientific) and nuclease free water (up to 20 µL). The reaction was conducted at 37 °C for 1 hour, followed by heating to 75 °C for 10 minutes. DNA was used in the subsequent ligation without purification or preparation.

Ligations were performed in 200 µL PCR tubes, using 20 µL of each phosphorylation reaction mixture, alongside 10 µL of a 0.1 mM solution of OH3'BB2' (CGTA GTACATGC). To the DNA strands was added 10X T4 DNA ligase buffer (9 µL, 400 mM Tris-HCl, 100 mM $MgCl_2$, 100 mM DTT, 5 mM ATP), water (up to 90 µL) and T4 DNA Ligase (3 µL, 30 Weiss U/µL). Ligations were conducted at 25 °C for 16 hours, followed by heating to 75 °C for 10 minutes. The product was then visualised by gel

electrophoresis, showing a band at ca. 60 base pairs in length (expected 59 and 63 base pairs), before being purified by EtOH precipitation. The resulting pellet was reconstituted in water (9 µL) for the initial amide coupling.

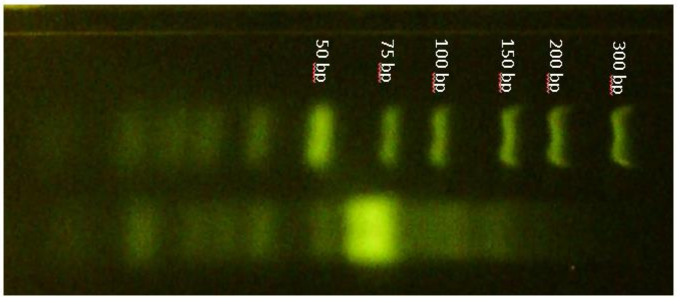

HATU (5.7 mg, 15 µmol) and Cbz-Valine-OH (3.73 mg, 15 µmol) were added to a 50 µL glass insert for a Para-dox^{TM} 96-well micro photoredox plate. 5% TPGS-750-M (21 µL) and DNA (9 µL, 0.11 mM in H_2O) were added to the vial, followed by lutidine (6.92 µL, 60 µmol). Samples were vortexed for 30 seconds each, and then heated in a Para-dox^{TM} 96-well micro photoredox plate at 45 °C for 16 hours. Samples were then diluted to 200 µL with H_2O; DCM (2 x 400 µL) was added, and the samples were vortexed. The organic layer was discarded, the sample was filtered through a hydrophilic PTFE filter and then precipitated according to the general ethanol precipitation procedure, and reconstituted in water (10 µL) for subsequent Cbz-deprotection.

To a 50 µL glass insert for a Para-dox^{TM} 96-well micro photoredox plate was added 10% wt Pd/C (5 µL, 400 mM in H_2O), 5% TPGS-750-M (12 µL), amide coupling product (10 µL, 0.1 mM in water). Samples were vortexed for 30 seconds each, then ammonium formate (3 µL, 5.3 M in water) was added, and the samples were vortexed for a further 10 seconds. Reactions were then shaken at 1200 rpm, at room temperature in a PMS-1000i Microplate shaker for 2 hours. The samples were diluted to 200 µL with water, filtered through a hydrophilic PTFE filter and precipitated according to the general ethanol precipitation procedure. The pellet was reconstituted in water and further purified through a 10000 Da MW filter to yield **53** (0.59 nmol).

Cycle 3

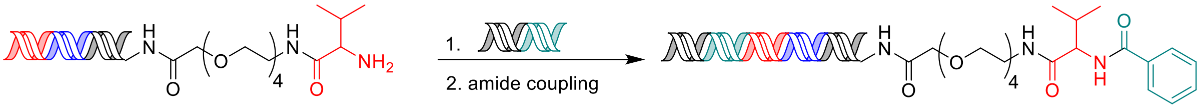

Sequences involved in the third ligation included **54**, primer 2 (P2), overhangs (OH) an;d building block 3 (BB3). Prior to ligations, the 5' terminus of any DNA strands that required ligating was phosphorylated; for cycle 3 this refers to sequences **54** and OH3BB3P2 (TACG AATATTGC TGACCTCAACTACATGGTCTACA). To the DNA strands (30 µM, 594 pmol in overall reaction media of 20 µL) was added PNK reaction buffer (2 µL, 500 mM Tris-HCl [pH 7.6 at 25 °C], 100 mM $MgCl_2$, 50 mM DTT, 1 mM spermidine), ATP (2 µL, 10 Mm, Thermo Scientific), T4 Polynucleotide Kinase (1 µL, 10U/µL. Thermo scientific) and nuclease free water (up to 20 µL). The reaction was conducted at 37 °C for 1 hour, followed by heating to 75 °C for 10 minutes. DNA was used in the subsequent ligation without purification or preparation.

Ligations were performed in 200 µL PCR tubes, using 20 µL of each phosphorylation reaction mixture, alongside 6 µL of a 0.1 mM solution of P2'BB3' (TGTAGACCATGTAGTTGAGGTCA

GCAATATT). To the DNA strands was added 10X T4 DNA ligase buffer (9 µL, 400 mM Tris-HCl, 100 mM $MgCl_2$, 100 mM DTT, 5 mM ATP), water (up to 90 µL) and T4 DNA Ligase (3 µL, 30 Weiss U/µL). Ligations were conducted at 25 °C for 16 hours, followed by heating to 75 °C for 10 minutes. The ligation product was then visualised by gel electrophoresis, showing a band at ca. 100 base pairs in length (expected 94 base pairs), alongside previously unreacted shortmers from earlier stages in the synthesis, before being purified by EtOH precipitation. The resulting pellet was then reconstituted in water (9 µL) for the terminal amide coupling.

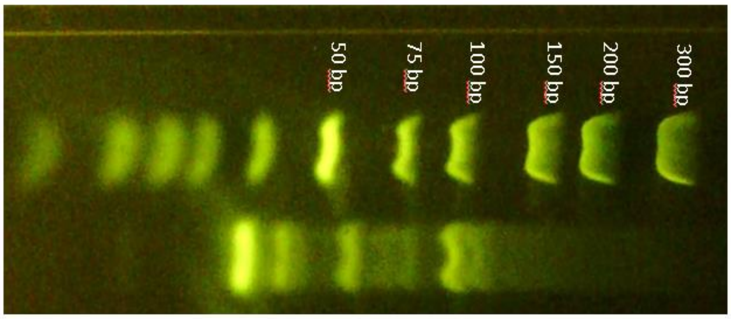

HATU (5.7 mg, 15 µmol) and Benzoic acid (1.83 mg, 15 µmol) were added to a 50 µL glass insert for a Para-dox™ 96-well micro photoredox plate. 5% TPGS-750-M (21 µL) and DNA (9 µL, 0.06 mM in H_2O) were added to the vial, followed by lutidine (6.92 µL, 60 µmol). Samples were vortexed for 30 seconds each, and then heated in a Para-dox™ 96-well micro photoredox plate at 45 °C for 16 hours. Samples were then diluted to 200 µL with H_2O; DCM (2 x 400 µL) was added, and the samples were vortexed. The organic layer was discarded, the sample was filtered through a hydrophilic PTFE filter and then precipitated according to the general ethanol precipitation procedure. The product was redissolved in water and purified further with a 10000 Da MW filter to yield **54** (0.34 nmol).

PCR and Sequencing

PCR amplification was performed in a 50 µL reaction mixture containing AmpliTaq Gold® 360 Master Mix (25 µL, ThermoFisher) and 1 µg (1.7 µl) of **54**. Amplification was conducted using either 0.2 µM (0.1 µL) or 2 µM (1 µL) primers concentration along with a negative control with the primers omitted (replaced with water). Thermal cycling conditions consisted of 10 minutes at 95 °C, followed by 40 cycles of 30 s at 95 °C, 30 s at 55 °C and 1 minutes at 72 °C, with a final extension time of 420 seconds at 72 °C. Analysis by gel electrophoresis on a 4% agarose gel showed that there was a clear band around 140-150 base pairs in length. The expected length of the DNA strand post amplification using the 33-base pair NGS extensions was 148-base pairs. The PCR product was analysed by NGS (Genewiz, South Plainfield, NJ, USA), >70% of 338596 reads corresponded to the expected sequence [TGTAGACCATGTAGTTGAGGTCAGCAATATTCGTAGTACATGCTAGGGCGTGTGCATACCAAATCCGTTCACACCGACCT (substrate strand)].

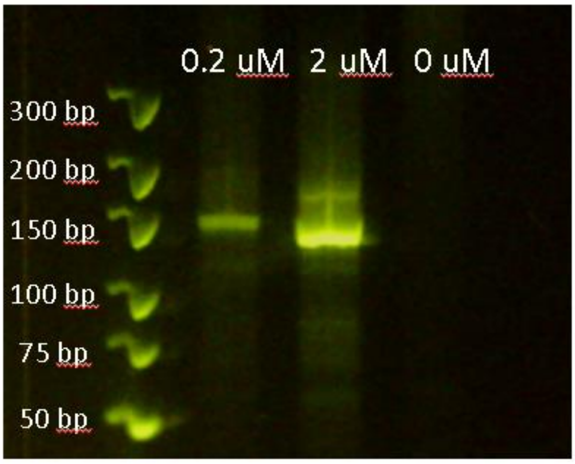

Sequences and frequencies of the top 10 most frequent reads for the substrate strand (total reads 338596):

Sequence	Count	Frequency
TGTAGACCATGTAGTTGAGGTCAGCAATATTCGTAGTACATGCTAGGGCGTGTGCATACCAAATCCGTTCACACCGACCT	243461	0.719
TGTAGACCATGTAGTTGAGGTCAGCAATATTCGTAGTACATGCCGTAGTACATGCTAGGGCGTGTGCATACCAAATCCGTTCACACCGACCT	5509	0.016
TTCCCCAAAGAAAGCTCATGGCCCAACCACACCACAACCGGAGTATCAGCATCATGCTCCCATAATGGGGAAAGCAGTTTTTACAAAAATTTGCTATGGCTGACGGGGAAGAATGGTTTGTACCCAA	2105	0.006
TGTAGACCATGTAGTTGAGGTCAGCAATATTCGTATACATGCTAGGGCGTGTGCATACCAAATCCGTTCACACCGACCT	1547	0.005
CAGCCTGCTGCAGATTGCCGGAGACCTGGAAGACCTAGAGCAAGCCCTGAATAAGACCAGCACCAAGCTTTCCCTGGGCTCCGGTTCCTGCTCCGCTATCATCAATCAAGTTGCTGCCAGGCGCACG	1222	0.004
TACGTCAGCATCCTAGGCTCGGAAGAGAACGAGTTCCCTGCCACAAGGGCGAGCCGCGATCCTCGATCTCAGAAGCCGTATCAAGCAAGGGGCTATAACACAGCCGAAGCTGCCACATTCCCCAAG	1015	0.003
TGTAGACCATGTAGTTGAGGTCAGCAATATTCGTAGACATGCTAGGGCGTGTGCATACCAAATCCGTTCACACCGACCT	874	0.003
TGTAGACCATGTAGTTGAGGTCAGCAATATTCGTAGCACACGCCCTAGTACATGCTAGGGCGTGTGCATACCAAATCCGTTCACACCGACCT	847	0.003
TGTAGACCATGTAGTTGAGGTCAGCAATATTCGTAGCACATGCTAGGGCGTGTGCATACCAAATCCGTTCACACCGACCT	818	0.002
TGTAGACCATGTAGTTGAGGTCAACAATATTCGTAGTACATGCTAGGGCGTGTGCATACCAAATCCGTTCACACCGACCT	788	0.002

Sequences and frequencies of the top 10 most frequent reads for the complementary strand (total reads 338596):

Sequence	Count	Frequency
AGGTCGGTGTGAACGGATTTGGTATGCACACGCCCTAGC ATGTACTACGAATATTGCTGACCTCAACTACATGGTCTAC A	244878	0.723
AGGTCGGTGTGAACGGATTTGGTATGCACACGCCCTAGC ATGTACTACGGCATGTACTACGAATATTGCTGACCTCAAC TACATGGTCTACA	5532	0.016
CGCTATTAGATTTCCATTTGCCTCAAATATTATTGTATCCC CGGGTTCAAGTAGAGTCCAGTAGTAGTTGATTCTTCCTTC TTGATCTCTTACTTTGGGTCTTTTGGCTATTTCTGGGGTGA	1676	0.005
AGGTCGGTGTGAACGGATTTGGTATGCACACGCCCTAGC ATGTATACGAATATTGCTGACCTCAACTACATGGTCTACA	1560	0.005
GATGATGCGTAGCATGTTCTGGTAGGAGTTCCATGTGTTG TGTGCCACCAGGAGGTCACGTGCGCCTGGCAGCAACTTG ATTGATGATAGCGGAGCAGGAACCGGAGCCCAGGGAAAG	1206	0.004
TTGAAAGGGAAACGATTGAAGTCAGACGTGCCTAACCGG GTCCAGCCTTTTGGTGTACCCCCGGTGGCAGGCTAGCAT CGATTTTGGGGGTCGGATAAGGGCTTGGGGAATGTGGCA	958	0.003
AGGTCGGTGTGAACGGATTTGGTATGCACACGCCCTAGC ATGTCTACGAATATTGCTGACCTCAACTACATGGTCTACA	902	0.003
AGGTCGGTGTGAACGGATTTGGTATGCACACGCCCTAGC ATGTACTAGGGCGTGTGCTACGAATATTGCTGACCTCAAC TACATGGTCTACA	854	0.003
AGGTCGGTGTGAACGGATTTGGTATGCACACGCCCTAGC ATGTGCTACGAATATTGCTGACCTCAACTACATGGTCTAC A	820	0.002
AGGTCGGTGTGAACGGATTTGGTATGCACACGCCCTAGC ATGTACTACGAATATTGTTGACCTCAACTACATGGTCTAC A	794	0.002

Quantitation of DNA by qPCR

A three building block headpiece was prepared (see S43-S47). This was divided equally into two and one sample was subjected to the hydrogenation conditions (2% TPGS-750-M, procedure S20). Each sample was resuspended in 100 μL of H_2O. 2 μL of each sample was taken and made up to 200 μL with H_2O. The two samples were subjected to quantitative PCR analysis using triplicates using a Bio-Rad CFX96™ real time system.

For each well contained 10 μL of ThermoFischer SYBR™ green PCR master mix, 0.2 μL of forward primer (10 μM), 0.2 μL of reverse primer (10 μM), 7.6 μL H_2O 7.6 μL and 2 μL sample mixture.

qPCR cycling conditions:

Step	Temperature (°C)	Time	Cycle
1	95	3 min	1
2	95	45 secs	40
3	61	40 secs	40
4	72	40 secs	40

The results were analyzed using BioRad CFX manager™ software #1845000. The percentage of amplifiable DNA was determined by calculating the difference in cycle threshold (Ct) between the two samples and transforming to the power 2.

**Sample**	**Run 1**	**Run 2**	**Run 3**	**Mean Ct**	**SD**	**dCt**	**2^-dCt**	**Percentage**
**SM**	14.22	14.21	14.30	14.24	0.05	0.00	1.00	100
**Hydrog**	14.32	14.34	14.31	14.32	0.015	0.08	0.95	95

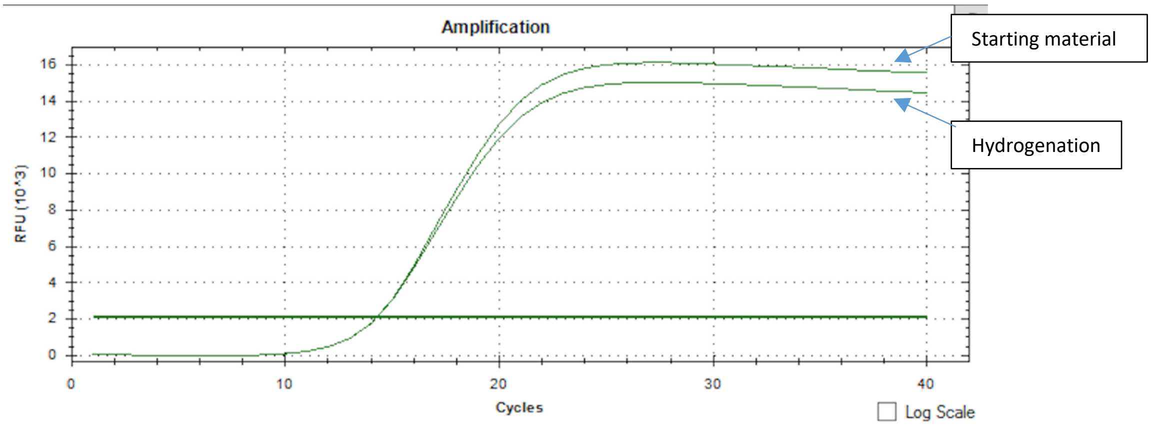

Supplement: Supplementary file 1 — Supporting Information [file ANIE-61-0-s001.pdf]
